# Foxd3 controls heterochromatin‐mediated repression of repeat elements and 2‐cell state transcription

**DOI:** 10.15252/embr.202153180

**Published:** 2021-10-04

**Authors:** Deepika Puri, Birgit Koschorz, Bettina Engist, Megumi Onishi‐Seebacher, Devon Ryan, Mamilla Soujanya, Thomas Montavon

**Affiliations:** ^1^ Department of Epigenetics Max Planck Institute for Immunobiology and Epigenetics Freiburg Germany; ^2^ National Centre for Cell Science Savitribai Phule Pune University Pune India; ^3^ Centre for Cellular and Molecular Biology Habsiguda India; ^4^ Present address: Novartis Institute for Biomedical Research (NIBR) Basel Switzerland; ^5^ Present address: Genedata AG Basel Switzerland

**Keywords:** 2‐cell‐like cells, Foxd3, heterochromatin, MERVL, transcription factor, Chromatin, Transcription & Genomics, Development, Stem Cells & Regenerative Medicine

## Abstract

Repeat element transcription plays a vital role in early embryonic development. The expression of repeats such as MERVL characterises mouse embryos at the 2‐cell stage and defines a 2‐cell‐like cell (2CLC) population in a mouse embryonic stem cell culture. Repeat element sequences contain binding sites for numerous transcription factors. We identify the forkhead domain transcription factor FOXD3 as a regulator of major satellite repeats and MERVL transcription in mouse embryonic stem cells. FOXD3 binds to and recruits the histone methyltransferase SUV39H1 to MERVL and major satellite repeats, consequentially repressing the transcription of these repeats by the establishment of the H3K9me3 heterochromatin modification. Notably, depletion of FOXD3 leads to the de‐repression of MERVL and major satellite repeats as well as a subset of genes expressed in the 2‐cell state, shifting the balance between the stem cell and 2‐cell‐like population in culture. Thus, FOXD3 acts as a negative regulator of repeat transcription, ascribing a novel function to this transcription factor.

## Graphical abstract

The forkhead domain transcription factor FOXD3 is involved in the heterochromatin‐mediated repression of major satellite repeats and MERVL, thereby counteracting the transcription of 2‐cell state promoting genes in mouse embryonic stem cells.

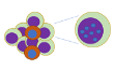

## Introduction

Repetitive elements including tandem repeats and interspersed repeats constitute up to 45% of the mouse genome (Biémont, 2010). In most somatic cells, repeat elements are repressed by a combination of epigenetic modifications such as DNA methylation and histone modifications such as H3K9me3, H4K20me3 and H3K27me3 (Peters *et al*, 2001; Martens *et al*, 2005; Kato *et al*, 2007; Mikkelsen *et al*, 2007). In early embryonic development however, these repeat elements are de‐repressed. The activation of specific repeats such as pericentric major satellite repeats (MSRs) and the endogenous retrovirus MERVL at the 2‐cell embryonic stage along with thousands of zygotic genes, accompanied by the clearance of maternal transcripts, constitutes an essential transcriptional milestone in embryonic development, called zygotic gene activation (ZGA) (Probst *et al*, 2010; Macfarlan *et al*, 2012; Burton & Torres‐Padilla, 2014; Dang‐Nguyen & Torres‐Padilla, 2015; Ishiuchi *et al*, 2015; Jukam *et al*, 2017). Additionally, mouse embryonic stem cells (mESCs), that have the property of self‐renewal and differentiation into all three germ layers, also exist as a heterogeneous population in culture, consisting of a small fraction (1–5%) of cells that resemble the more totipotent 2C‐like cells (2CLCs) (Macfarlan *et al*, 2012). These cells are characterised by an expanded potency and activation of MSRs and MERVL along with a specific set of genes such as the Zscan4 family, Zfp352 and Pramel7 (Macfarlan *et al*, 2012; Genet & Torres‐Padilla, 2020). Studies have shown that MERVL activation is sufficient for the conversion of mESCs to 2CLCs (Yang *et al*, 2020). During embryonic development, downregulation of MSR and MERVL is concomitant with increased LINE1 transcription, which facilitates the exit from the 2‐cell state and contributes to ESC self‐renewal (Percharde *et al*, 2018). The importance of repeat element transcription in early development and stem cell function underscores the need to understand the regulation of repeat transcription. Previous studies have demonstrated the presence of numerous transcription factor (TF)‐binding sites within repeat element sequences (Bourque *et al*, 2008; Bulut‐Karslioglu *et al*, 2012). Transposable elements have emerged as a hub for transcription factor binding and assembly of transcription complexes (Hermant & Torres‐Padilla, 2021). For example, PAX3 and PAX9 bind to and repress MSRs in mouse embryonic fibroblasts, and this repression is essential for maintaining the integrity of heterochromatin (Bulut‐Karslioglu *et al*, 2012). REX1 represses endogenous retroviruses (ERVs) in mESCs and preimplantation embryos through the binding and recruitment of YY1 and YY2 (Guallar *et al*, 2012). In contrast, ZSCAN4 and DUX act as positive regulators of MERVL transcription in mESCs and early embryos (Hendrickson *et al*, 2017; Zhang *et al*, 2019). Human ERVH and ERVK sequences contain binding sites for pluripotency TFs OCT4 and SOX2 which facilitate ERV transcription (Kunarso *et al*, 2010; Fort *et al*, 2014).

This study focuses on the identification and characterisation of novel transcription factors that influence repeat element expression. Our analysis identifies the forkhead domain‐containing transcription factor FOXD3 as a novel regulator of repeat elements. FOXD3 is crucial for maintaining ES cell pluripotency (Hanna *et al*, 2002; Liu & Labosky, 2008). In stem cells, FOXD3 plays a bimodal role as an activator or a repressor in a context‐dependent manner by enhancer decommissioning and recruitment of chromatin modulators such as the histone demethylase LSD1, chromatin remodelling factor BRG1 and histone deacetylases (HDACs) to target sites (Krishnakumar *et al*, 2016a; Respuela *et al*, 2016; Sweet, 2016). We observe that in mESCs, FOXD3 binds to and represses MERVL and to a lesser extent, MSRs. Depletion of FOXD3 leads to a significant de‐repression of MERVL and MSRs and a concomitant increase in a subset of 2CLC genes. Mechanistically, FOXD3 represses MERVL and MSRs by interacting with and recruiting the heterochromatin histone methyltransferase SUV39H1, which establishes the repressive H3K9me3 mark to the target sites. This study is a novel report of FOXD3 as a heterochromatin‐mediated repressor of repeat element transcription and 2CLC gene expression in mouse embryonic stem cells.

## Results and Discussion

### FOXD3 binds to major satellite repeats and MERVL

**Figure EV1:**
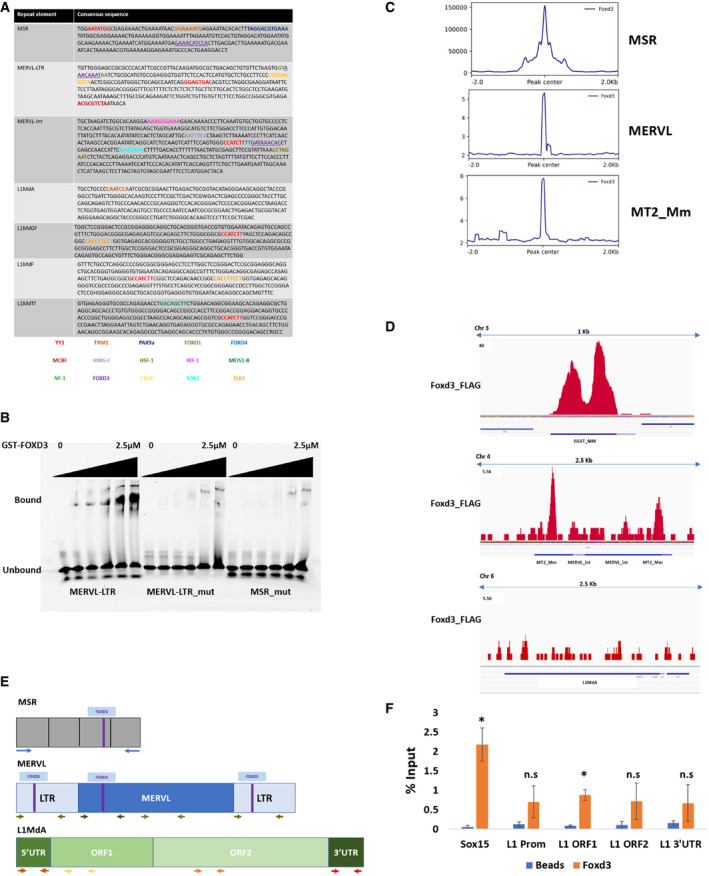
FOXD3 binds to MERVL and MSR elements Consensus sequences for MSR, MERVL‐LTR, MERVL‐int, L1MdA, L1MdGf, L1MdTf and L1MdF marked with TF‐binding sites predicted by PROMO.Electrophoretic mobility shift assay (EMSA) with increasing concentrations (0, 15 nM, 30 nM, 60 nM, 1.25 μM and 2.5 μM) of GST‐FOXD3 and fixed concentration (50 nM) of 5′‐Cy5‐labelled dsDNA oligonucleotides (35 bp each) from MERVL‐LTR, mutated MERVL‐LTR and mutated MSR sequences. The experiment was repeated 3 times.Metadata profile showing enrichment of FOXD3 over the MSR, MERVL and MT2_Mm consensus sequence including ± 2 kb flanking the FOXD3 peak on each repeat. *Y*‐axes represent relative signal intensity.FOXD3 enrichment detected by FLAG‐FOXD3 ChIP‐Seq on representative MSR, MERVL and L1MdA loci.Schematic (not to scale) representing MSR, MERVL and LINE sequences depicting FOXD3‐binding sites and primers used for qPCR.ChIP‐qPCR enrichment of FOXD3 in mESCs using primers specific for LINE sequences. *Sox15* promoter primers are used as positive control. Data are represented as percentage of input, and the average of three biological replicates is plotted. Error bar indicates standard error of the mean (SEM). Asterisks indicate statistically significant differences compared with no antibody control levels (*P < 0.05, paired *t*‐test).

**Figure 1:**
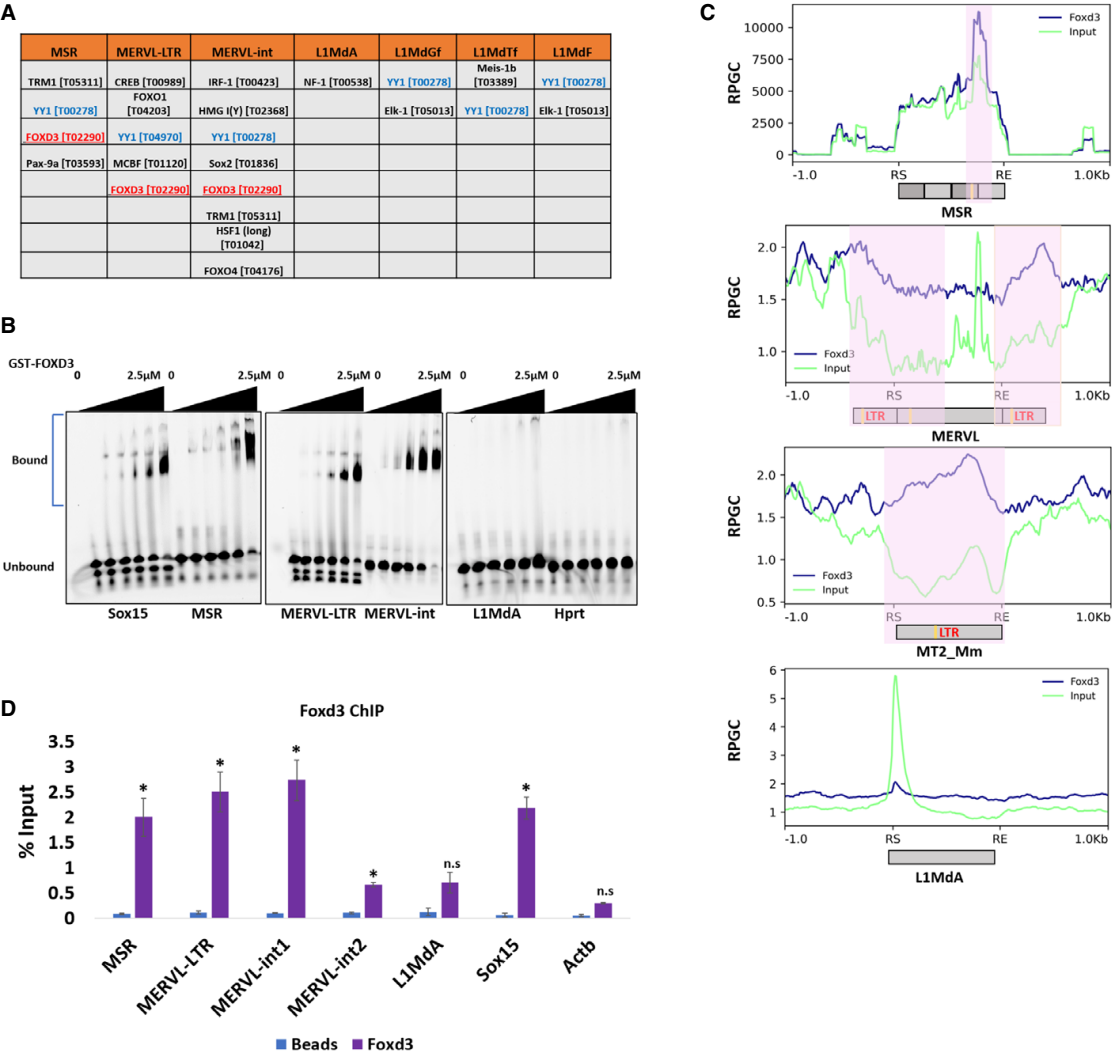
FOXD3 binds to MERVL and MSR elements Transcription factor‐binding sites with Transfac IDs predicted by PROMO in repeat element consensus sequences.Electrophoretic mobility shift assay (EMSA) with increasing concentration (0, 15 nM, 30 nM, 60 nM, 1.25 μM and 2.5 μM) of GST‐FOXD3 and fixed concentration (50nM) of 5′‐Cy5‐labelled dsDNA oligonucleotides (35 bp each) from *Sox15* promoter (positive control), MSR, MERVL‐LTR, MERVL‐int, L1MdA and *Hprt* promoter (negative control). The experiment was repeated three times.Metadata analysis plots of FOXD3 ChIP‐Seq (blue) and input (green) over MSR, MERVL‐int, MT2_Mm and L1MdA consensus sequences including 1 kb flanking the start (RS) and end (RE) of each repeat. *Y*‐axes indicate normalised reads per genome coverage (RPGC). Predicted FOXD3‐binding sites are marked in yellow and enrichment of FOXD3 over input is indicated in red. FOXD3 enrichment is seen in MSR, MERVL‐int, MERVL‐LTR but not in L1MdA.ChIP‐qPCR enrichment of FOXD3 in mESCs using primers specific for MSR, MERVL‐LTR, MERVL‐int (including and excluding the FOXD3 cognate motif), L1MdA promoter sequences. *Sox15* promoter and *Actb* promoter primers are used as positive and negative control, respectively. Data are represented as percentage of input, and the average of three biological replicates is plotted. Error bar indicates standard error of the mean (SEM). Asterisks indicate statistically significant differences compared with no antibody control levels (*P < 0.05, paired *t*‐test).

Mouse embryonic stem cells are an important developmental model to study the genetic and epigenetic mechanisms regulating the pluripotent state and cell fate transitions. We chose to identify putative TF‐binding sites in MSR, MERVL‐LTR, MERVL‐int and LINE 5′UTR sequences as the expression of these elements is critical in early development. We obtained consensus sequences for these repeats from Repbase (Jurka *et al*, 2005) (Fig EV1A) and subjected them to the TF‐binding site prediction tool PROMO (Messeguer *et al*, 2002). The TFs that were predicted with a *P*‐value < 0.05, a dissimilarity percentage < 2 and were expressed in mouse ES cells based on published data (Bulut‐Karslioglu *et al*, 2014a), were considered as significant hits. Binding sites for TFs such as TRM1, YY1, SOX2 and FOXD3 were predicted in the repeat sequences (Figs 1A and EV1A). Interestingly, binding sites for the TF YY1 were predicted in MSR, MERVL as well as promoters of the transcriptionally competent LINE1 sequences. The 5′UTR of L1MdA was largely devoid of TF‐binding sites. The identification of YY1‐binding sites in a majority of repeat elements is consistent with previous reports (He *et al*, 2019) and points to a potential common role of this TF in repeat regulation. The forkhead transcription factor FOXD3 emerged as a promising candidate as reports indicate that FOXD3 plays a crucial role in maintaining ES cell pluripotency (Hanna *et al*, 2002; Krishnakumar *et al*, 2016a; Respuela *et al*, 2016). FOXD3 is expressed in mESCs and has been shown to bind to and recruit chromatin factors such as HDACs, BRG1 and LSD1 to their target sites (Krishnakumar *et al*, 2016a). Our analysis predicted the presence of the FOXD3‐binding site GAATGTTT (Transfac ID T02290) in MSR, MERVL‐LTR and MERVL‐int sequences (Figs 1A and EV1A). We examined the binding of FOXD3 to MSR and MERVL DNA *in vitro*. Increasing concentrations (0–2.5 μM) of the recombinant GST‐FOXD3 fusion protein was incubated with double‐stranded 5′Cy5‐labelled DNA oligonucleotides representing MSR, MERVL‐LTR, MERVL‐int and L1MdA, in addition to *Sox15* (known target for FOXD3 as positive control) (Plank *et al*, 2014) and *Hprt* (negative control) (Table EV2). A concentration‐dependent mobility shift of the DNA–protein complex was seen for *Sox15*, MSR, MERVL‐LTR and MERVL‐int oligonucleotides but not for L1MdA and *Hprt* oligonucleotides (Fig 1B). We confirmed the specificity of binding by incubating recombinant GST‐FOXD3 with DNA oligonucleotides representing MERVL and MSR with a mutated FOXD3‐binding site. The mobility shift of the DNA–protein complex seen in the presence of the intact FOXD3‐binding site was no longer detected with the mutated oligonucleotides (Fig EV1B). To confirm FOXD3 binding to MERVL and MSRs in mESCs, we analysed published FOXD3 chromatin immunoprecipitation (ChIP‐Seq) data (GEO data set GSE58408, Data ref: Krishnakumar *et al*, 2016b) and observed robust enrichment of FOXD3 over the MSR, MERVL‐LTR, MERVL‐int and the solo MERVL‐LTR (MT2_Mm) consensus sequences, but not over L1MdA. (Figs 1C and EV1C and D). While MSR and MERVL‐LTR sequences displayed clear peaks of FOXD3 enrichment around the predicted binding site, MERVL‐int showed a broader enrichment, covering an area beyond the TF‐binding site (Fig 1C). As we used all the annotated MERVL‐int repeats (full‐length as well as truncated) instead of just intact repeats for computing and plotting the FOXD3 enrichment; the data were scaled to fit repeat start and end, irrespective of the repeat's size, which may affect the alignment of the peaks and lead to the appearance of broader enrichment. Indeed, when the data were centred around FOXD3 peaks, a sharp localised FOXD3 enrichment was seen over all three repeats (Fig EV1C). Additionally, while we identified FOXD3‐binding sites based on stringent cut‐offs in our TF prediction algorithm, we do not exclude the possibility of other FOXD3‐binding sites along the repeat sequence which were not predicted by our algorithm, but would still be enriched for FOXD3. Analysis of individual MERVL loci, however (Fig EV1D), indicates that FOXD3 is largely enriched over defined regions of MERVL which presumably correspond to the FOXD3‐binding site. Other reports identifying TF binding over repeat elements also displayed broader peaks not localised to predicted binding sites (Shi *et al*, 2019; Zhang *et al*, 2019; Wolf *et al*, 2020). We further validated this enrichment by performing ChIP with an antibody against FOXD3 in mESCs. In agreement with our previous observations, we detected significant FOXD3 enrichment over MERVL‐LTR, MERVL‐int and MSR, but not over L1MdA using primers described in Table EV2 (Figs 1D and EV1E and F). These results indicate that the transcription factor FOXD3 binds to a subset of repeat elements, especially MERVL and MSRs in mouse embryonic stem cells.

### Foxd3 deletion leads to de‐repression of MERVL and a subset of 2CLC genes

**Figure 2:**
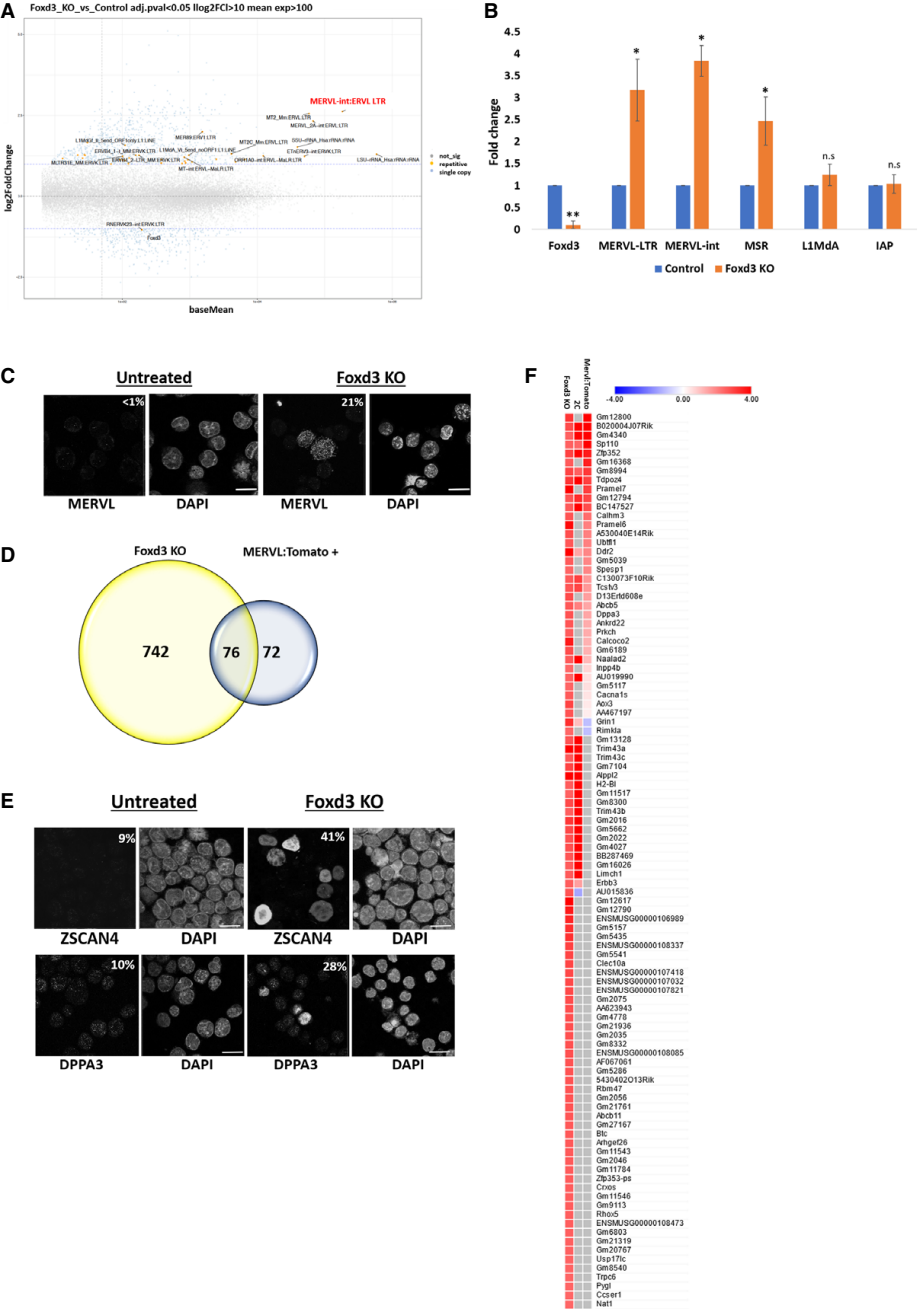
MSR, MERVL and 2CLC‐specific genes are upregulated in Foxd3 KO cells MA plot showing analysis of up‐ and downregulated genes and repeats in total RNA‐Seq preparations of control and Foxd3 KO cells. Significantly dysregulated genes are shown in blue and repeats are shown in yellow. The most highly upregulated repeat is marked in red. Data from two biological replicates are plotted.Validation of select upregulated repeats in Foxd3 KO cells by RT–qPCR. The data are plotted as average fold change relative to control, after normalisation to *Gapdh*. Error bars indicate SEM (*n* = 3 biological replicates). Asterisks indicate statistically significant differences when compared to control (**P < 0.005, *P < 0.05, paired *t*‐test).Immunofluorescence analysis of the MERVL‐gag protein in control and Foxd3 KO cells. Nuclei are labelled with DAPI. The percentage of counted cells (˜ 150 cells per experiment) exhibiting immunofluorescence signal is indicated. Scale bar represents 10 μm, *n* = 3 biological replicates.Venn diagram representing the overlap between genes upregulated in Foxd3 KO cells (yellow) and in MERVL: Tomato+ cells (representing 2CLCs, blue) (Macfarlan *et al*, 2012).Immunofluorescence analysis of 2CLC markers ZSCAN4 and DPPA3 in control and Foxd3 KO cells. Nuclei are labelled with DAPI. The percentage of counted cells (˜ 150 cells per experiment) exhibiting the immunofluorescence signal is indicated. Scale bar represents 10 μm, *n* = 3 biological replicates.Heat map depicting the comparative gene expression profiles of the top 100 upregulated genes in Foxd3 KO cells. The columns indicate relative expression in Foxd3 KO cells, 2‐cell stage embryos and MERVL: tomato+ cells.

**Figure EV2:**
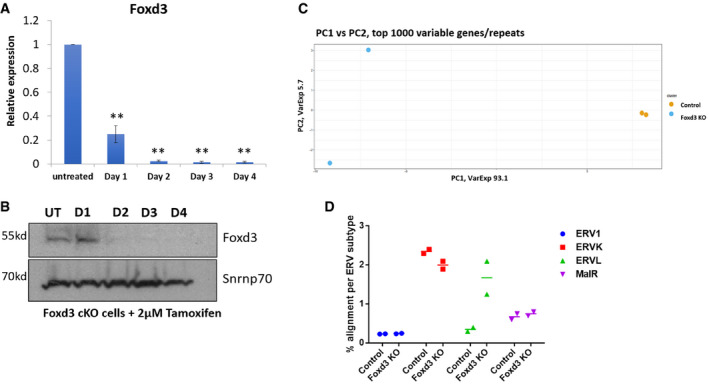
MERVL is upregulated in Foxd3 KO cells RT–qPCR analysis of Foxd3 in control and Foxd3 KO cells. The data are plotted as average fold change relative to control, after normalisation to *Gapdh*. Error bars indicate SEM (*N* = 3 biological replicates). Asterisks indicate statistically significant differences compared with untreated cells (**P < 0.005, paired *t*‐test).Immunoblot of protein lysates prepared from untreated and 4‐OHT treated cells, probed with Foxd3 antibody. Snrnp70 is used as loading control. The experiment was repeated three times using biological replicates.Principal Component Analysis of the RNA‐Seq data from control (Orange) and Foxd3 KO (Blue) indicating percentage variance for two principal components.Expression levels for each ERV subtype plotted for control and Foxd3 KO cells. *Y*‐axis represents percentage alignment for each repeat subtype from RNA‐Seq data. Individual data points from two biological replicates are plotted. Source data are available online for this figure.

*Foxd3* null embryos die after implantation, and ES cell lines cannot be maintained (Hanna *et al*, 2002; Liu & Labosky, 2008). To understand the effect of Foxd3 on repeat elements in mESCs, we used a conditional gene inactivation approach. *Foxd3fl*/*fl;Cre‐ER* ES cells (Liu & Labosky, 2008); hence, forth referred to as Foxd3 cKO cells (A gift from Patricia Labosky) exhibited a tamoxifen‐dependent deletion of *Foxd3*, and an almost complete loss of Foxd3 was seen both at transcript and protein levels by day 2 of 4‐hydroxytamoxifen (4‐OHT) treatment (Fig EV2A and B). As the cells underwent extensive apoptosis after day 4 of 4‐OHT treatment, all the experiments were carried out at day 2. We performed total RNA‐Seq analysis of Foxd3 cKO cells in the absence (control) and presence (Foxd3 KO) of 4‐OHT as described in Materials and Methods. Principal component analysis revealed that control and Foxd3 KO cells exhibited significantly distinguishable expression profiles (Fig EV2C). As this study was aimed at understanding the role of Foxd3 in repeat regulation, we investigated the expression levels of annotated repeat elements. 38 repeat elements were significantly upregulated, and 1 repeat element was significantly downregulated in Foxd3 KO cells (Dataset EV1). The most significantly upregulated repeats belonged to the ERV superfamily (Fig 2A), and among the ERVs, ERVL‐LTR and ERVL‐int were most highly upregulated (Figs 2A and EV2D). We validated these results by RT–qPCR which also showed significant upregulation of MERVL‐LTR, MERVL‐int and MSR but not L1MdA in Foxd3 KO cells (Fig 2B). While the MSR consensus sequence contained the FOXD3‐binding site (Fig 1A) and FOXD3 was found to bind to MSR in our previous experiments (Fig 1), MSR was not significantly upregulated in our RNA‐Seq data analysis. We therefore limited our further analysis to the more robust upregulation of MERVL upon Foxd3 depletion. We addressed the expression of MERVL at protein level by performing immunofluorescence of control and Foxd3 KO cells using an antibody against the MERVL‐gag protein. We observed that while less than 1% cells expressed MERVL in control, this number increased up to 21% in Foxd3 KO cells (Fig 2C). These results demonstrate that in mouse ES cells, FOXD3 represses the MERVL retroviral element and to a lesser extent, major satellite repeats.

**Figure EV3:**
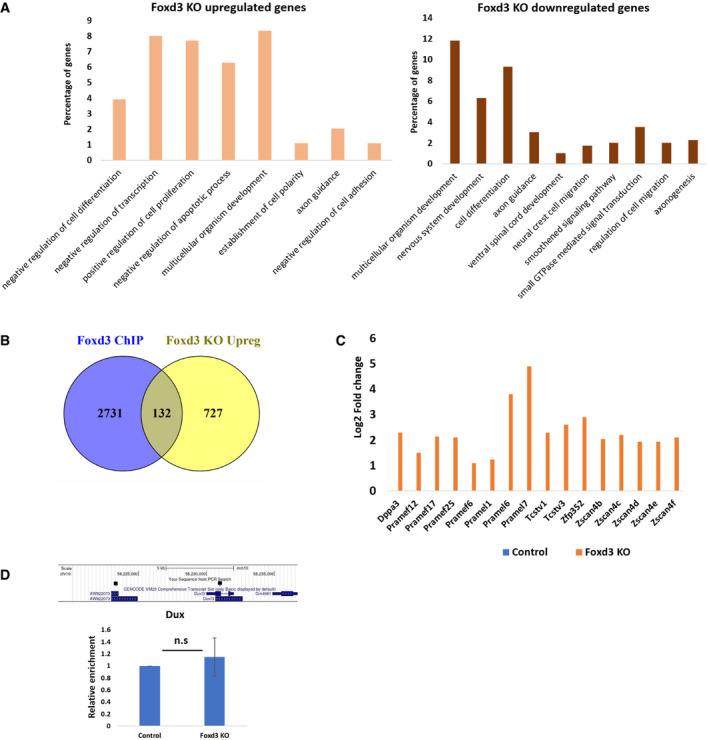
2CLC genes are upregulated in Foxd3 KO cells GO protein classes significantly enriched in genes upregulated (left panel) and downregulated (right panel) in Foxd3 KO cells. The *Y*‐axis represents percentage of genes belonging to each protein class.Venn diagram comparing Foxd3 bound genes (Krishnakumar *et al*, 2016) with genes upregulated in Foxd3 KO cells.Expression of representative 2CLC genes in Foxd3 KO cells. The *Y*‐axis represents log_2_ fold change in Foxd3 KO cells compared with control cells.Schematic for RT–qPCR primers specific for Duxf3 and AW822073 (black squares). RT–qPCR analysis depicting Dux expression in control and Foxd3 KO cells. The data are plotted as average fold change relative to control, after normalisation to *Gapdh*. Error bars indicate SEM (*n* = 3 biological replicates). Statistical test: paired *t*‐test.

In addition to repeat elements, we also conducted comparative gene expression analysis in control and Foxd3 KO cells. Foxd3 KO cells exhibited a significant upregulation of 858 genes and downregulation of 413 genes (Dataset EV2), indicating that in these cells, FOXD3 plays primarily a repressive role, in agreement with previous reports (Krishnakumar *et al*, 2016a; Respuela *et al*, 2016). Functional classification of the differentially expressed genes in Foxd3 KO cells using DAVID (Huang *et al*, 2009) showed that genes involved in the negative regulation of cell differentiation, cell proliferation, transcription and negative regulation of apoptosis were significantly (*P* < 0.001) upregulated in Foxd3 KO cells, while cell differentiation, neural crest migration and nervous system development genes were downregulated in Foxd3 KO cells (Fig EV3A). This is consistent with the role of Foxd3 in stem cell maintenance and neural crest development (Liu & Labosky, 2008; Lukoseviciute *et al*, 2018). We also compared Foxd3 KO upregulated genes with Foxd3 target genes identified by ChIP‐Seq (GEO data set GSE58408, Data ref: Krishnakumar *et al*, 2016b) and found that of the 858 genes upregulated in Foxd3 KO, 132 genes were also targets of FOXD3 (Fig EV3B, Table EV1) indicating that their regulation may be a result of direct binding and repression by FOXD3. FOXD3 may control the transcription of other genes indirectly by binding to and sequestering activator proteins, acting as a cofactor in repressive complexes or repressing enhancers that control gene expression (Lam *et al*, 2013; Krishnakumar *et al*, 2016a; Respuela *et al*, 2016). This is also evident from the data reported by Krishnakumar *et al* (2016a) where of the 7,000 peaks detected as FOXD3 targets, only 2,863 peaks were mapped to genes. FOXD3 is known to bind to and regulate gene expression by long range enhancer decommissioning (Krishnakumar *et al*, 2016a; Respuela *et al*, 2016), and our results demonstrating the role of FOXD3 in the regulation of MSR and MERVL repeats reinforce the indirect mode of FOXD3‐mediated regulation of gene expression.

As MERVL was the most upregulated repeat element in Foxd3 KO cells, and MERVL activation is a hallmark of the 2‐cell embryonic stage, we compared the genes upregulated in Foxd3 KO with genes upregulated in 2CLCs. Macfarlan *et al* (2012) described MERVL:tomato‐positive cells that represent the population of 2CLCs in an ES cell culture. Comparative analysis showed that 51% of genes upregulated in MERVL: tomato‐positive cells were also significantly upregulated in Foxd3 KO cells (Fig 2D). These included 2C‐specific genes of the Zscan4 family, Prame family, Dppa3, Zfp352, as well as Tcstv1 and Tcstv3 (Figs 2E and 2F, and EV3C) (Macfarlan *et al*, 2012; Hendrickson *et al*, 2017). Immunofluorescence analysis of ZSCAN4 and DPPA3 revealed that these proteins were barely detectable in control cells, but expressed at high levels in 41% (ZSCAN4) and 28% (DPPA3) of Foxd3 KO cells (Fig 2E), suggesting that the balance between ES and 2CLC is shifted towards 2CLC in a Foxd3 KO cell population. We further compared the top 100 upregulated genes in Foxd3 KO cells with genes expressed in the embryo at the 2‐cell stage (Macfarlan *et al*, 2012) and observed that 56% of the top Foxd3 KO upregulated genes overlapped with either the 2C embryo or MERVL:tomato‐positive cell expression data (Fig 2F). This indicates that the most highly upregulated genes in Foxd3 KO cells are 2CLC genes, underscoring the contribution of Foxd3 in repressing the conversion of mESCs to 2CLC.

DUX has been described as a positive regulator of MERVL and the 2CLC state (Hendrickson *et al*, 2017). The murine *Dux* tandem repeat encodes two main transcripts, full‐length *Dux* (or *Duxf3*), a variant *Gm4981,* lacking the first homeodomain, and an additional EST, AW822073 (Iaco *et al*, 2020). Our data depict a modest upregulation of the EST AW822073 in Foxd3 KO cells (Dataset EV2) and not the major *Dux* transcripts, indicating that the effect of Foxd3 on MERVL may not be Dux‐dependent. To confirm this, we designed primers common to *Duxf3* and AW822073 (Fig EV3D). RT–qPCR analysis revealed that Dux expression is unchanged in Foxd3 KO cells (Fig EV3D). FOXD3 therefore seems to function in a Dux‐independent pathway to repress MERVL, which is consistent with recent reports indicating that Dux expression is dispensable for zygotic gene activation (Chen & Zhang, 2019; Guo *et al*, 2019; Iaco *et al*, 2020). Further investigation of the effect of FOXD3 depletion in Dux knockout conditions would help validate the Dux‐independent regulation of MERVL by FOXD3. In addition to MERVL, studies have shown that transient activation of LINE1 RNA is required for the normal developmental progression of a 2C embryo (Jachowicz *et al*, 2017). LINE1 acts to repress Dux in mouse ES cells via a KAP1/nucleolin pathway (Percharde *et al*, 2018). While our RNA‐Seq analysis showed a modest upregulation of the 5′ UTR of L1MdA and L1MdGf in Foxd3 KO cells (Fig 2A), FOXD3 was not enriched over these regions (Fig 1) and RT–qPCR analysis showed that LINE element expression remains unchanged in Foxd3 KO cells (Fig 2B). This may maintain the repression of Dux and hence prevent a complete recapitulation of the 2C transcription profile, as 49% of the genes upregulated in the MERVL‐tomato‐positive cells did not appear to be Foxd3‐dependent (Fig 2D). Repeat element transcription, therefore, seems to be governed by a collaborative action of multiple modes of regulation and warrants comprehensive studies aimed at understanding the combinatorial regulation of repeats and in turn, the balance between mESCs and 2CLCS.

### MERVL de‐repression depends on direct binding of FOXD3

**Figure EV4:**
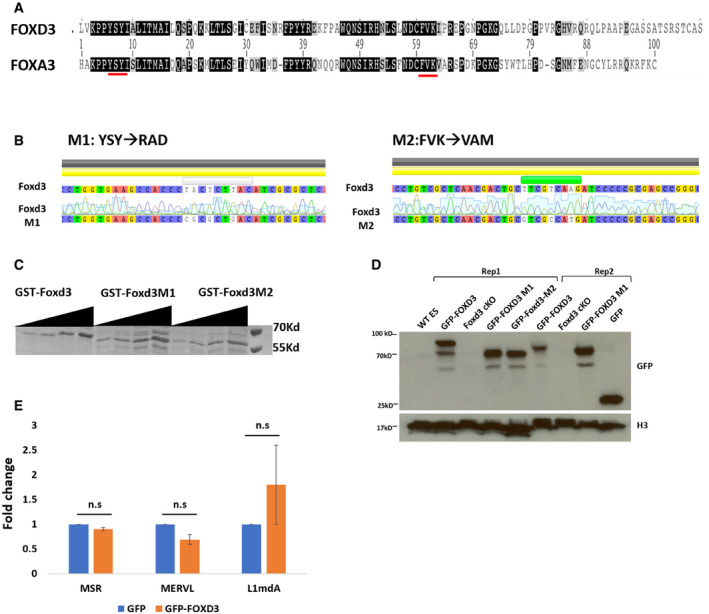
Establishment of mutant FOXD3 constructs and cell lines Conservation of YSY and FVK amino acids (underlined) between mouse FOXA3 and FOXD3 DNA‐binding domain sequences.DNA sequencing results indicating generation of Foxd3 M1 and M2 mutants.Coomassie staining indicating expression of recombinant GST‐FOXD3, GST‐FOXD3 M1 and GST‐FOXD3 M2. The experiment was repeated three times using biological replicates.Immunoblot (α GFP top panel and α H3 bottom panel) for lysates from Foxd3 cKO cells expressing GFP‐FOXD3, GFP‐FOXD3 M1 and GFP‐FOXD3 M2. Histone H3 levels are used as loading control. The experiment was repeated three times using biological replicates.RT–qPCR analysis depicting MSR, MERVL and L1MdA expression in control and GFP‐FOXD3 overexpressing Foxd3 cKO cells. The data are plotted as average fold change relative to control, after normalisation to *Gapdh*. Error bars indicate SEM (*n* = 3 biological replicates). Statistical test: paired *t*‐test. Source data are available online for this figure.

**Figure 3:**
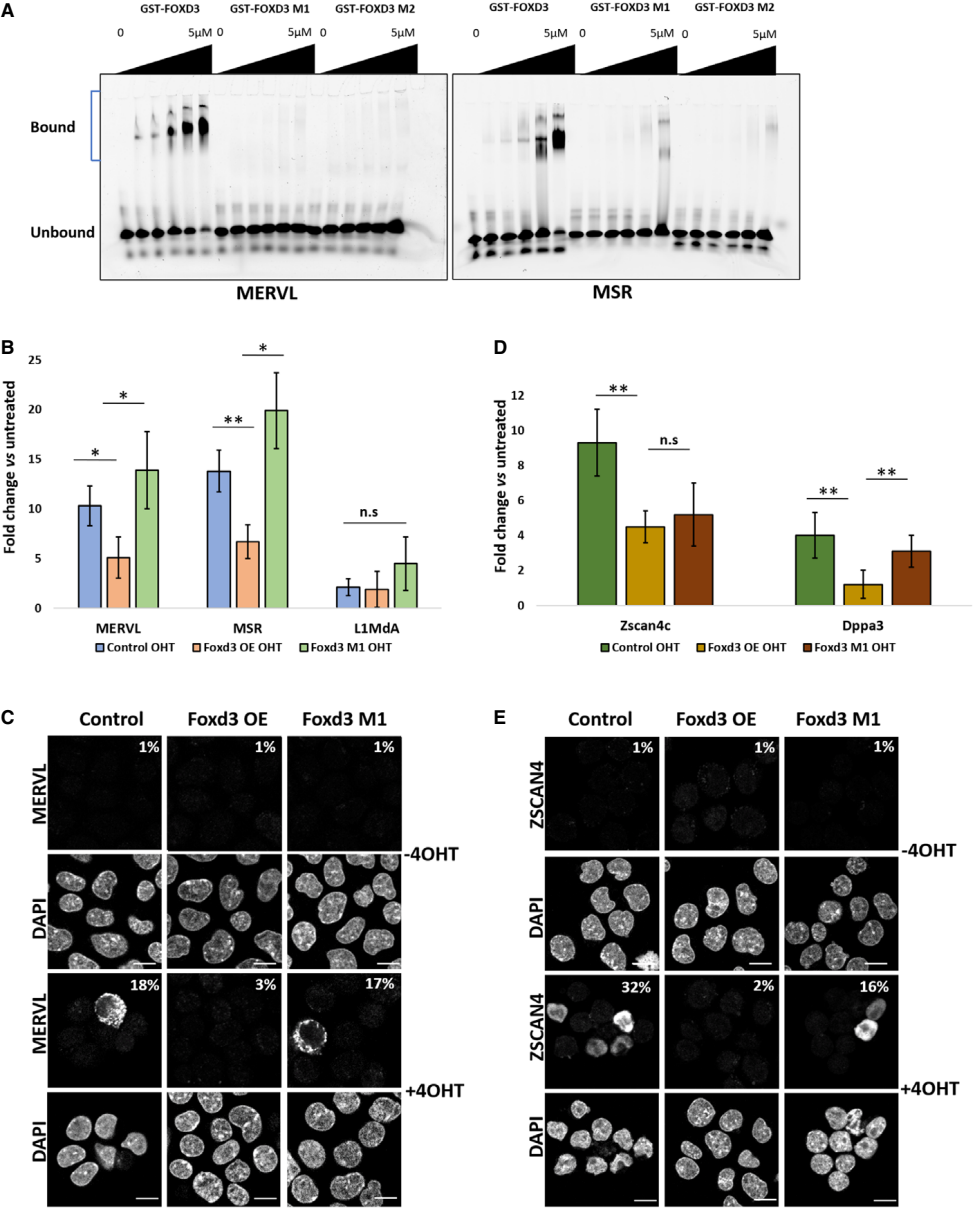
FOXD3 DNA‐binding domain is essential for regulation of MERVL and 2CLC genes Electrophoretic mobility shift assay (EMSA) with increasing concentrations (0, 30 nM, 60 nM, 1.25 μM, 2.5 μM and 5 μM) of recombinant GST‐FOXD3, DNA‐binding mutants GST‐FOXD3 M1 and GST‐FOXD3 M2 and fixed concentration (50 nM) of 5′‐Cy5‐labelled dsDNA oligonucleotides (35 bp each) representing MERVL and MSR. The experiment was repeated three times.RT–qPCR analysis of MERVL, MSR and L1MdA in Foxd3 cKO cells, Foxd3 cKO cells overexpressing full‐length FOXD3 or FOXD3 M1 without and with 4‐OHT. Data are represented as fold change relative to respective untreated cells after normalising to *Gapdh*. The average data for three biological replicates are plotted, and error bars represent standard error mean (SEM). Asterisks indicate statistically significant differences. (**P < 0.005, *P < 0.05 paired *t*‐test).Immunofluorescence analysis of MERVL‐gag protein in Foxd3 cKO cells, Foxd3 cKO cells overexpressing full‐length FOXD3 or FOXD3 M1 without and with 4‐OHT. Nuclei are labelled with DAPI. The percentage of counted cells (˜ 150 cells per experiment) exhibiting immunofluorescence signal is indicated. Scale bar represents 10 μm *n* = 3 biological replicates.RT–qPCR analysis of, 2C genes *Zscan4c* and *Dppa3* in Foxd3 cKO cells, Foxd3 cKO cells overexpressing full‐length FOXD3 or FOXD3 M1 without and with 4‐OHT. Data are represented as fold change relative to respective untreated cells after normalising to *Gapdh*. The average data for three biological replicates are plotted, and error bars represent standard error mean (SEM). Asterisks indicate statistically significant differences. (**P < 0.005, *P < 0.05 paired *t*‐test).Immunofluorescence analysis of the ZSCAN4 protein in Foxd3 cKO cells, Foxd3 cKO cells overexpressing full‐length FOXD3 or FOXD3 M1 without and with 4‐OHT. Nuclei are labelled with DAPI. The percentage of counted cells (˜ 150 cells per experiment) exhibiting fluorescence signal is indicated. Scale bar represents 10 μm *n* = 3 biological replicates.

To determine whether the upregulation of MERVL is a result of direct occupancy of FOXD3 on the repeat sequences, we generated two DNA‐binding mutants of FOXD3 based on previous reports identifying the amino acids YSY and FVK in the DNA‐binding domain of FOXA3 as being essential for the DNA‐binding function of the protein (Clevidence *et al*, 1993). These amino acids were also conserved in mouse FOXD3 (Fig EV4A). We generated recombinant GST‐tagged wild‐type FOXD3 as well as FOXD3 mutant proteins carrying YSY→RAD (M1) or FVK→VAM (M2) mutations (Fig EV4B and C). Gel shift assays with FOXD3 M1 and M2 proteins indicated that both mutants failed to bind labelled MERVL and MSR oligonucleotides (Fig 3A). To determine whether a DNA‐binding deficient FOXD3 could affect repeat element expression and gene transcription, we established Foxd3 cKO mESC lines overexpressing GFP‐tagged wild‐type FOXD3, FOXD3 M1 and FOXD3 M2 (Fig EV4D). Overexpression of FOXD3 in Foxd3 cKO cells did not significantly alter MERVL or MSR expression (Fig EV4E), and Foxd3 depletion by tamoxifen led to an expected de‐repression of MERVL in non‐rescued cells. This de‐repression of MERVL was significantly weaker in cells overexpressing wild‐type GFP‐FOXD3, but not in GFP‐FOXD3 mutant (M1), which showed a similar upregulation as Foxd3 KO. A similar effect was seen for MSRs, but the LINE1 promoter sequence, which was devoid of the FOXD3‐binding site, was not upregulated in any of the conditions (Fig 3B). In the absence of the rescue construct, FOXD3 depletion resulted in MERVL‐gag protein expression in 18% of the cells. In contrast, in cells overexpressing wild‐type GFP‐FOXD3, MERVL‐gag was detected in less than 3% of the cells. This rescue was not apparent in GFP‐FOXD3 M1 expressing cells (Fig 3C). These results indicate that the DNA‐binding domain of FOXD3 is required for the repression of MERVL and MSR, and suggest a direct regulation of these repeat elements by FOXD3.

We investigated whether the differences in MERVL were reflected in the expression of 2C genes. Upon tamoxifen treatment, 2C‐specific genes such as *Zscan4c* and *Dppa3* showed an upregulation in Foxd3 cKO cells, reduced expression in wild‐type GFP‐FOXD3 overexpressing cells and an upregulation in GFP‐FOXD3 M1 cells (Fig 3D). We also observed a similar result at the protein level where the percentage of FOXD3‐depleted cells expressing ZSCAN4 was 32% in the absence of the rescue construct, reduced to 2% in the presence of wild‐type GFP‐FOXD3 and increased to 16% in the presence of GFP‐FOXD3 M1 (Fig 3E), emphasising the requirement of an intact DNA‐binding domain of FOXD3 in repressing MERVL and 2C genes.

Interestingly, Foxd3 KO led to a higher percentage of ZSCAN4 expressing cells than MERVL expressing cells (Figs 2C and E, and 3C and E). There are conflicting reports regarding the sequence of activation of MERVL and ZSCAN4 proteins during the conversion of mESCs to 2CLCs. Reports indicate that ZSCAN4 activates MERVL, and ZSCAN4‐positive cells act as an intermediate state between ES cells and 2CLCs (Zhang *et al*, 2019; Fu *et al*, 2020). In contrast, analysis of transcription dynamics across a pseudo‐time trajectory reveals that MERVL expression precedes Zscan4 family gene expression during ESC to 2CLC transition (Eckersley‐Maslin *et al*, 2016). Our comparative analysis between FOXD3‐binding genes and genes upregulated in Foxd3 KO cells (Fig EV3B and Table EV1) indicates that Zscan4 family genes are not targets for FOXD3 binding. Activation of Zscan4 in Foxd3 KO cells, therefore, maybe dependent on MERVL expression. Our study leaves open the possibility of a feedback loop between MERVL and Zscan4 family genes that are activated upon MERVL expression in Foxd3 KO cells. Notably, upon 4‐OHT treatment, FOXD3 M1 cells showed lower expression of 2C genes compared with Foxd3 KO cells (Fig 3D). It is possible that in addition to the regulation mediated by MERVL, these genes may be regulated by FOXD3 in a binding‐independent manner. Our studies demonstrate that FOXD3 represses MSR and MERVL in mESCs. Thus, it may stand to reason that overexpression of FOXD3 in WT cells would affect these repeats. However, we observed that the levels of MSR, MERVL and 2CLC genes were largely unchanged in untreated Foxd3 cKO cells overexpressing GFP‐FOXD3 (Figs 3C and E, and EV4E). This may indicate that the available FOXD3‐binding sites on MERVL and MSR have already been occupied by the endogenous protein leading to a balanced regulation of their transcription, rendering the introduction of the additional protein ineffective. FOXD3 has also been shown to bind mutually exclusive enhancer sequences in naïve and primed ES cells (Krishnakumar *et al*, 2016a; Respuela *et al*, 2016). FOXD3 overexpression may lead to the occupancy of the loci which were unbound in mESCs. Further comprehensive understanding of the transcriptional dynamics may shed more light on the temporal activation and the combinatorial action of gene‐repeat expression in the transition from ES to the 2CLC state.

### FOXD3 binds to and recruits SUV39H1 to MERVL and MSR

**Figure EV5:**
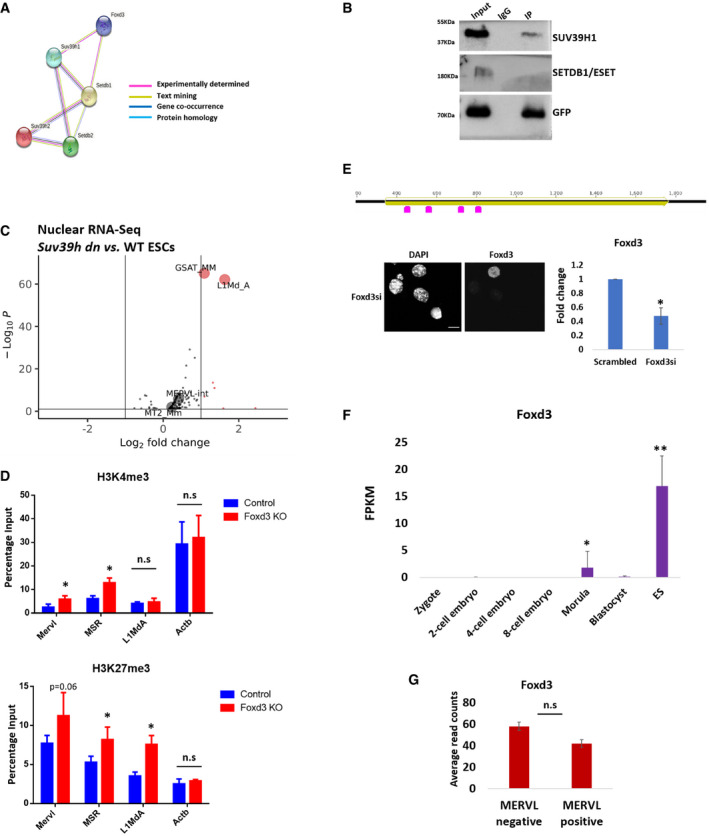
Role of FOXD3 in SUV39H1‐mediated regulation of MSR and MERVL STRING analysis to predict putative interaction between FOXD3, SUV39H1, SUV39H2, SETDB1 and SETDB2. FOXD3 was significantly predicted to interact with SUV39H1 and SETDB1 (*P*‐value < 0.01). The criteria used for predicting interaction are depicted.GFP immunoprecipitation analysis using Foxd3 cKO mESCs expressing GFP‐FOXD3. Immunoblot results using antibodies against SUV39H1, SETDB1 and GFP are depicted. The experiment was repeated three times using biological replicates.Volcano plot depicting significantly upregulated and downregulated repeats in *Suv39h dn* ES cells compared with wild‐type ES cells determined by RNA‐Seq analysis of nuclear RNA. MSR (GSAT_MM), MERVL‐int, L1MdA and MT2_Mm are labelled.ChIP‐qPCR depicting enrichment of H3K4me3 (top panel) and H3K27me3 (bottom panel) over MERVL, MSR and L1MdA in control and Foxd3 KO cells. *Actb* promoter is used as negative control. Data are represented as average percentage input of 3 biological replicates. Error bars represent SEM (*P < 0.05 paired *t*‐test).Schematic depicting the location of Foxd3 siRNAs (pink) along the Foxd3 locus (upper panel). Immunofluorescence detected by FOXD3 antibody in Foxd3i cells. Nuclei are stained with DAPI. Scale bar = 10 μm (lower left). RT–qPCR analysis of Foxd3 expression in control and Foxd3i cells. Data are represented as fold change compared with control (scrambled) after normalisation to *Gapdh*. *N* = 3 biological replicates (lower right). Error bars represent SEM. (*P < 0.05 paired *t*‐test).Bar graph depicting Foxd3 expression at different stages of embryonic development. *Y*‐axis depicts average FPKM from 4 independent single cell RNA‐Seq data sets. Error bar represents standard deviation. (*P < 0.05, **P < 0.005, unpaired *t*‐test).Bar graph depicting relative Foxd3 expression in MERVL‐positive and MERVL‐negative cells. The data are represented as the average read counts obtained from RNA‐Seq data from three biological replicates. Error bars represent SEM. Statistical test: unpaired *t*‐test. Source data are available online for this figure.

**Figure 4:**
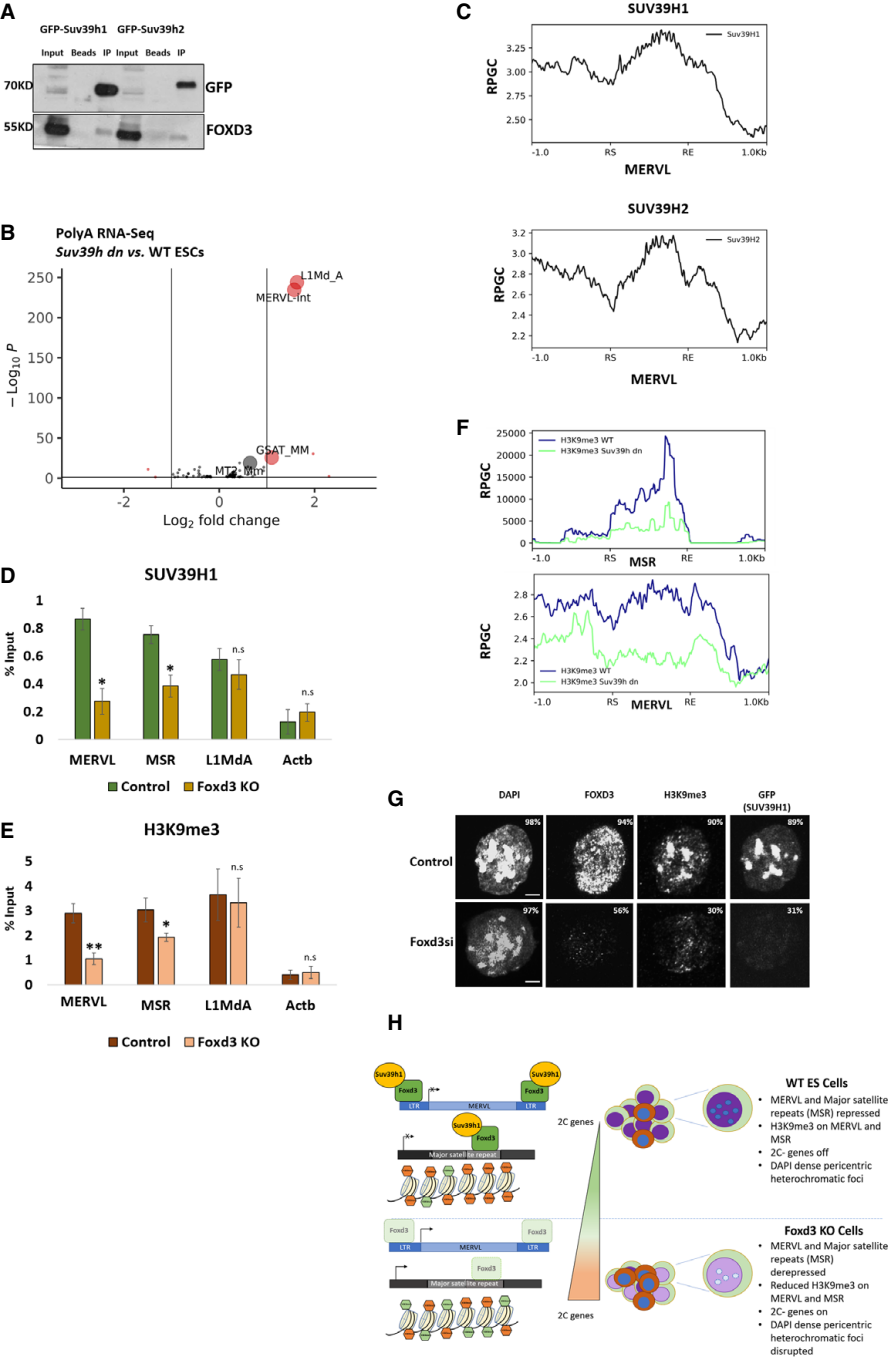
FOXD3 recruits SUV39H1 to repeat elements GFP immunoprecipitation analysis using GFP‐SUV39H1 and GFP‐SUV39H2‐expressing *Suv39h dn* mESCs. The top panel represents immunoblot using GFP antibody, and the bottom panel represents immunoblot using FOXD3 antibody. The experiment was repeated three times using biological replicates.Volcano plot depicting significantly upregulated and downregulated repeats in *Suv39h dn* ES cells compared to wild‐type ES cells determined by RNA‐Seq analysis. MSR (GSAT_MM), MERVL‐int, L1MdA and MT2_Mm are labelled.Metadata profile showing enrichment of SUV39H1 and H2 over the MERVL consensus sequence including 1 kb sequences flanking the start (RS) and end (RE) of the repeat. *Y*‐axes represent reads per genome coverage (RPGC).ChIP‐qPCR analysis of endogenous SUV39H1 enrichment over MERVL, MSR and L1MdA in control and Foxd3 KO cells. *Actb* promoter is used as negative control. Data are represented as average percentage input from three biological replicates. Error bar indicates SEM (*P < 0.05 paired *t*‐test).ChIP‐qPCR analysis revealing H3K9me3 enrichment over MERVL, MSR and L1MdA in control and Foxd3 KO cells. *Actb* promoter is used as negative control. Data are represented as average percentage input from three biological replicates. Error bar indicates SEM (**P < 0.005, *P < 0.05 paired *t*‐test).Metadata analysis of H3K9me3 enrichment over MSR and MERVL consensus sequences in WT mESCs (blue) and *Suv39h dn* mESCs (green). Relative enrichment is plotted along the repeat start (RS) and repeat end (RE) including ± 1 kb flanking sequences. *Y*‐axis represents reads per genome coverage (RPGC).Immunofluorescence analysis of control and Foxd3i GFP‐SUV39H1 expressing *Suv39h dn* mESCs stained with DAPI, FOXD3 and H3K9me3. The percentage of counted cells (˜ 150 cells per experiment) exhibiting fluorescence signal is indicated. Scale bar = 2 μm. *n* = 3 biological replicates.FOXD3 represses MERVL and MSR in mouse ES cells: FOXD3 (green) binds to MERVL and MSR and recruits SUV39H1 (yellow) to the repeats. SUV39H1 facilitates repression of the repeats by establishing H3K9me3 (orange hexagons). Foxd3 depletion disrupts the recruitment of SUV39H1 and leads to a reduction in H3K9me3 over MERVL and MSR. This in addition to an increase in H3K4me3 (green hexagons) on the region results in the activation of MSR and MERVL and a concomitant increase in 2CLC‐specific genes, shifting the balance between mESCs (Green and purple) and 2CLCs (Brown and blue). This is accompanied by a perturbation of the classical condensed heterochromatic foci in the nucleus. Foxd3 thus helps maintain ES cells in a pluripotent state by regulating MERVL and MSR. Source data are available online for this figure.

Our data show that FOXD3 represses MERVL and MSR in mouse ES cells. ERVs and MSRs are largely regulated by the activity of heterochromatin histone methyltransferases such as SETDB1, SETDB2, SUV39H1 and SUV39H2 that establish H3K9me3 to repress transcription (Karimi *et al*, 2011; Bulut‐Karslioglu *et al*, 2014a; Groh & Schotta, 2017). FOXD3 has been shown to interact with and recruit chromatin‐modifying proteins to target sites (Krishnakumar *et al*, 2016a; Respuela *et al*, 2016). To determine whether FOXD3 could interact with SETDB1/2 or SUV39H1/H2, we performed STRING analysis (Szklarczyk *et al*, 2019), which predicted a putative interaction of FOXD3 with SETDB1 and SUV39H1 (Fig EV5A). To validate the FOXD3‐SUV39H1 interaction in mESCs, we used mESCs devoid of both SUV39H1 and SUV39H2 (*Suv39h dn*) expressing either exogenous GFP‐SUV39H1 or GFP‐SUV39H2 under the control of a β‐actin promoter (Velazquez Camacho *et al*, 2017) as well as Foxd3 cKO cells overexpressing GFP‐FOXD3. GFP immunoprecipitation followed by immunoblot demonstrated a sub‐stoichiometric interaction of FOXD3 with SUV39H1 and SUV39H2 (Figs 4A and EV5B), but none with SETDB1 (Fig EV5B).

Bioinformatic analysis of RNA‐Seq of Poly‐A selected RNA from WT and *Suv39h dn* mESCs (GEO data set GSE57092, Data ref: Bulut‐Karslioglu *et al*, 2014b) identified MERVL as one of the most highly upregulated repeats in *Suv39h dn* cells similar to Foxd3 KO cells, while MSR was the among the most highly upregulated repeats in *Suv39h dn* cells when nuclear RNA was used for RNA‐Seq (Figs 4B and EV5C), indicating that MERVL and MSR repeats are regulated by the SUV39H family proteins. L1MdA was robustly upregulated in *Suv39h dn* cells in both data sets. To determine the functional relevance of FOXD3 interaction with SUV39H1/2, we analysed published ChIP‐Seq data for HA‐tagged SUV39H1 and H2 (GEO data set GSE57092, Data ref: Bulut‐Karslioglu *et al*, 2014b) and observed the enrichment of SUV39H1 and SUV39H2 at MERVL in mESCs (Fig 4C). While the role of SUV39H enzymes in the repression of MSRs and LINE 1 repeats is known (Peters *et al*, 2001; Bulut‐Karslioglu *et al*, 2014a), we report MERVL as a novel target for SUV39H‐mediated repression in mESCs. To understand the role of FOXD3 in the SUV39H‐mediated repression of repeats, we performed ChIP in control and Foxd3 KO cells using an antibody specific for SUV39H1. In control cells, we observed an enrichment of SUV39H1 over MERVL, MSR and L1MdA. This enrichment was significantly reduced for MERVL and MSR in Foxd3 KO cells, but not for L1MdA, which is not a target for FOXD3 binding, indicating that FOXD3 participates in recruiting SUV39H1 to MSR and MERVL (Fig 4D).

Finally, to determine the histone modification profile over MERVL and MSR in Foxd3 KO cells, we performed ChIP for select activating and repressive histone modifications. Our experiments revealed that consistent with the increase in transcription and reduced recruitment of SUV39H1, the H3K9me3 mark decreased over MERVL and MSR repeats in Foxd3 KO cells (Fig 4E). This was also accompanied by a modest increase in H3K4me3 as well as H3K27me3 (Fig EV5D). LSD1/KDM1A, a histone demethylase with catalytic activity towards H3K4, has been shown to repress MERVL in mESCs (Macfarlan *et al*, 2011a). Interestingly, LSD1 interacts with FOXD3 in mESCs (Respuela *et al*, 2016). The enrichment of H3K4me3 over MERVL in Foxd3 KO cells may be a consequence of reduced FOXD3‐mediated LSD1 occupancy over these repeat regions. We also see an enrichment of H3K27me3 over MSR and MERVL in Foxd3 KO, which is consistent with previous reports indicating that an increase in H3K27me3 can follow a reduction in H3K9me3 (Peters *et al*, 2003), underscoring the plasticity between these systems. Interestingly, when we analysed published ChIP‐Seq data for H3K9me3 in WT and *Suv39h dn* ES cells (GEO data set GSE57092, Data ref: Bulut‐Karslioglu *et al*, 2014b), we observed that while MSRs show a drastic reduction in H3K9me3 enrichment in *Suv39h dn* cells, this reduction is less robust for MERVL (Fig 4F). This is consistent with the fact that while MSR transcription is primarily regulated by SUV39H enzymes (Peters *et al*, 2001; Bulut‐Karslioglu *et al*, 2014a; Velazquez Camacho *et al*, 2017), MERVL regulation depends on redundant functions of other HMTs such as SETDB1 (Karimi *et al*, 2011; Wu *et al*, 2020), which remain unperturbed in *Suv39h dn* cells. This also explains the persistence of low levels of H3K9me3 over MERVL and the incomplete recapitulation of the 2CLC transcription profile in FOXD3 KO cells (Figs 2F and 4E). Combinatorial depletion of FOXD3 along with SETDB1 may shed light on the redundant functions of heterochromatin‐mediated regulation of MERVL. Our data indicate that H3K9me3 is reduced over pericentric MSRs in Foxd3 KO cells. We confirmed this by performing RNAi‐based knock‐down of FOXD3 in *Suv39h dn* mESCs expressing GFP‐SUV39H1 (Fig EV5E). Immunofluorescence analysis revealed that in cells lacking FOXD3, the pericentric localisation of H3K9me3 as well as SUV39H1 is severely disrupted (Fig 4G). Interestingly, the organisation of heterochromatin as depicted by DAPI dense foci remained largely unchanged, in agreement with previous reports (Peters *et al*, 2001). We also noted that a not all Foxd3i cells lose the pericentric H3K9me3 and SUV39H1 localisation (Fig 4D and G). This might point to alternate modes of recruitment of SUV39H1 to the pericentric regions. Reports have shown that MSR RNA associates with chromatin and forms RNA:DNA hybrids that stabilise the association of SUV39H enzymes to pericentric regions (Velazquez Camacho *et al*, 2017). We propose that establishment of stable heterochromatin at MSRs may be attributed to the redundant roles of TFs such as FOXD3, or PAX3 and PAX9 (Bulut‐Karslioglu *et al*, 2012) as well as non‐coding repeat RNA in the recruitment of HMTs. Our data also demonstrate that while MSRs, intact ERVs and LINE1 elements, are regulated by SUV39H methyltransferases (Bulut‐Karslioglu *et al*, 2012; Bulut‐Karslioglu *et al*, 2014a), they may be recruited to these loci using different modes as the SUV39H1 enrichment as well as H3K9me3 levels remain unchanged over the LINE1 promoter in Foxd3 KO cells.

MERVL transcription is known to be regulated by numerous mechanisms. Our data show that depleting FOXD3 results in a robust upregulation of MERVL in a subset of ES cells and an incomplete recapitulation of the 2CLC transcription profile. Other known activating and repressive modulators of MERVL such as DUX or SETDB1 are unchanged in Foxd3 KO cells. We analysed published data (Fan *et al*, 2015a; GEO data set GSE53386, Data ref: Fan *et al*, 2015b) to determine the expression of Foxd3 through different stages of embryonic development and found that Foxd3 expression begins at the morula stage and is the highest in ES cells (Fig EV5F). This suggests that while Foxd3 may not be crucial for the exit from the 2‐cell stage, it could facilitate the maintenance of the stem cell state and prevent reversion to the 2CLC state. Indeed, a modest reduction in Foxd3 expression is observed in a MERVL‐positive population of mESCs as compared to MERVL‐negative cells (GEO data set GSE33923, Data ref: Macfarlan *et al*, 2011b) (Fig EV5G). FOXD3 has been identified as a pioneer TF with an ability to bind condensed chromatin and precede the binding of other TFs (Zaret & Carroll, 2011; Krishnakumar *et al*, 2016a; Lukoseviciute *et al*, 2018). FOXD3 has also been shown to bind to mutually exclusive sites in different stages of development, providing a temporal context to FOXD3 regulation in embryonic development. Our study reveals a crucial layer to this tapestry by including pericentric MSRs and intergenic MERVL regions as targets of FOXD3‐mediated regulation, in addition to previously identified enhancers. The next logical step would be combinatorial perturbation of FOXD3 along with other regulatory modules that may facilitate a more robust acquisition of the 2CLC transcriptional repertoire and also may reveal potential connections with known (DUX, REX1) or novel regulatory molecules that work in concert to ultimately regulate cell fate. In conclusion, our data demonstrate that in mESCs, FOXD3 binds to and represses MERVL and MSR repeat elements by recruiting the heterochromatin histone methyltransferase SUV39H1 that establishes the H3K9me3 mark to these sites. In the absence of FOXD3, MERVL and MSR repeat elements are de‐repressed, and 2CLC genes are activated, skewing the mESC‐2CLC balance towards 2CLC (Fig 4H). As repeat element sequences contain numerous other TF‐binding sites, it is tempting to hypothesise a scenario where a combination of DNA‐binding proteins act in a context‐specific manner to recruit activating or repressive proteins to repeat elements and regulate their expression in ES cells. This study identifies a novel heterochromatin function for transcription factors and opens up further avenues into the investigation of transcription factor‐mediated control of repeat elements in regulating stem cell pluripotency and lineage commitment.

## Materials and Methods

### Cell culture

Foxd3 cKO cells obtained from Patricia Labosky lab were cultured using standard protocols on 0.2% gelatin‐coated plates. To induce Foxd3 knockout, cells were treated with 2 μM 4‐hydroxytamoxifen (Sigma‐T5648) changed daily. Cells were harvested for experiments on day 2 when Foxd3 was depleted at RNA and protein levels.

### Generation of recombinant GST‐FOXD3 and GFP‐FOXD3 fusion proteins

Wild‐type full‐length Foxd3 cDNA was obtained from OriGene (Catalog number MR222218) and amplified using Foxd3‐specific primers to be subcloned into the pGEX‐6P1 plasmid and verified by sequencing. M1 (YSYRAD) and M2 (FVKVAM) mutants were generated by site‐directed mutagenesis using specific primers. GST‐fusion proteins were expressed using protocols described in Velazquez Camacho *et al* (2017). Sequences encoding full‐length Foxd3, M1 and M2 mutants were subcloned into the pCAGGS‐EGFP vector as described in Velazquez Camacho *et al* (2017). These plasmids were transfected into Foxd3 cKO cells using Xfect transfection reagent (Clontech). The cells were kept under puromycin selection to obtain polyclonal cell lines. All cell lines were checked periodically for mycoplasma contamination.

### EMSA

35 nucleotide 5′‐Cy5‐labelled and HPLC‐purified DNA oligonucleotides were purchased from Sigma. To generate dsDNA, equimolar amounts of forward and reverse ssDNA oligonucleotides were mixed in 1xSSC buffer (150 mM NaCl, 15 mM sodium citrate) and incubated for 2 min at 90°C in a Thermomixer. The temperature was reduced to 60°C for 5 min and then further reduced to 20°C for 30 min. For EMSA, 50 nM of nucleic acids was mixed with increasing concentrations of recombinant proteins in a buffer containing 20 mM Tris–HCl pH 8.0, 100 mM KCl, 3mM MgCl2, 1 mM EDTA pH 8.0, 5% glycerol, 0.05% NP‐40, 2 mM DTT, 50 ng/ml yeast tRNA (Thermo Fisher) and 2.5 ng/ml BSA (NEB). Samples were incubated at 4°C with rotation for 1 h and resolved on a 4% polyacrylamide (60:1) gel (25 mM Tris–HCl, 200 mM glycine, 5% glycerol, 0.075% APS, 0.05% TEMED) in 12.5 mM Tris–HCl and 100 mM glycine. The Cy5 signal was scanned on a Typhoon FLA 9500 fluorescence scanner.

### Chromatin immunoprecipitation

Chromatin immunoprecipitation was performed as described in Bulut‐Karslioglu *et al* (2014a) with antibody‐specific optimisations. For histone modification as well as FOXD3 ChIP, trypsinised cells were fixed by incubating with 1% formaldehyde for 10 min. For SUV39H1 ChIP, trypsinised cells were incubated in DSG (Di (*N*‐succinimidyl glutarate)) (Synchem OHG) at a final concentration of 2 mM for 45 min at room temperature, washed twice with PBS and then incubated in 1% formaldehyde for 20 min at room temperature. Subsequent steps were performed as described in Bulut‐Karslioglu *et al* (2014a). The purified DNA was analysed by qPCR using specific primers as described in Table EV2. The antibodies used for ChIP are as follows: Foxd3: Merck Millipore, Catalog number AB5687, 4 μg. H3K9me3: crude serum, Antibody no 4861 (Jenuwein lab), 5 μl. H3K4me3: Diagenode, Catalog number C15410003‐50, 2 μg. H3K27me3: Antibody no 6523 (Jenuwein lab) 4 μg, Suv39h1: Sigma‐Aldrich (Merck), Catalog number 05‐615, 10 μl.

### ChIP‐Seq analysis

Fastq files were acquired from published data sets (Bulut‐Karslioglu *et al*, 2014a; Krishnakumar *et al*, 2016a). Fastp version 0.20.1 (Chen *et al*, 2018) was used to analyse the quality of the fastq reads and trim adapters from both ends. The processed.fastq files were mapped to mm10 using Bowtie 2 version 2.4.4 (Langmead & Salzberg, 2012) using “‐‐very‐sensitive” mode. To analyse repetitive sequences, reads aligning to multiple locations of a distinct repeat type were distributed randomly to these positions (Treangen & Salzberg, 2012; Bulut‐Karslioglu *et al*, 2014a). Reads aligning to mitochondrial DNA or to unassigned sequences were discarded. Reads mapping to the same location were not removed, to avoid biased representation between repetitive and unique sequences. Peak calling was done using ChIP and input reads with MACS2 version 2.2.7.1 to detect broad peak (H3K9me3, Suv39h1&2) or narrow peak (Foxd3) distributions without removing duplicate reads with a q‐value of 0.01 and an effective genome size of 2,654,910,000 bp. Alignment files for ChIP and input were normalised to 1X coverage using reads per genome coverage (RPGC) and converted into BigWig files with 50 bp bin size, using DeepTools version 3.5.1. (Ramírez *et al*, 2014). DeepTools “scale regions” was used to calculate ChIP or input signal over repeat start and repeat end of all annotated repeats of each repeat family (MERVL‐int, GSAT_Mm, MT2_Mm and L1MdA), and “reference‐point” was used to compute ChIP‐seq signal from peak centre with flanking ± 1 kb (Fig EV1C). DeepTools sub‐commands plotProfile were used to generate the plots of RPGC normalised data in input and ChIP over repeat elements (Figs 1C and 4C and F).

### RNA‐Seq

Total RNA was extracted from control and Foxd3 KO cells with TRIzol (Invitrogen), and DNA was digested with Turbo DNase (Ambion), followed by clean‐up with RNeasy MinElute Cleanup kit (Qiagen, 74204). Libraries were prepared using the TruSeq Stranded Total RNA Library Prep Gold (Illumina, 20020598) following Illumina protocols. The libraries were sequenced on a HiSeq2500 Illumina platform using a 100 bp paired‐end approach. Two biological replicates were sequenced per cell line.

### RNA‐Seq analysis

The sequencing reads were aligned to the mouse genome build mm10 using TopHat2 (Kim *et al*, 2013) with default parameters. Repeats and genes were quantified using TEtranscripts (Jin *et al*, 2015), and DEseq2 (Love *et al*, 2014) was used to determine differentially expressed repeat elements and genes. deepTools was used to construct BigWig files. Volcano plots (Figs 4B and EV5C) were generated using R package “EnhancedVolcano” with *P*‐value cut‐off of 0.01. Coverage tracks were visualised using IGV (Robinson *et al*, 2011). Detailed parameters are mentioned in Velazquez Camacho *et al* (2017). Heat map represented in Fig 2F was plotted using Morpheus: https://software.broadinstitute.org/morpheus

### Immunofluorescence

5 × 10^4^ ES cells were attached to gelatin‐coated glass slides by using Cytospin (Thermo Scientific). Cells were fixed in 4% para‐formaldehyde for 10 min at room temperature, washed three times with 1X PBS and permeabilised in a 0.05% Triton‐X solution for 5 min. Permeabilised cells were washed twice with 1X PBS and incubated in blocking solution (PBS/0.25% BSA/0.1% Tween‐20% and 10% normal goat serum) for 1 h at room temperature. The slides were incubated with primary antibodies overnight at 4°C. Slides were then washed with 1X PBS and incubated for 1 h at room temperature with appropriate fluorescently labelled secondary antibody. After washing with 1XPBS, slides were mounted with VECTASHIELD mounting medium containing DAPI. Cells were observed using a Confocal microscope (Zeiss Observer Z1) to detect the fluorescent signal. Images were captured at 63X magnification, analysed with Zen software 2011 SP3 (Black version) and depicted as “maximum intensity” projections from Z stacks of representative ES cells (*n* = 150). Final assembly of images was done using ImageJ. The primary antibodies used are as follows: ZSCAN4, Millipore, Catalog number AB4340, 1:250. MERVL‐gag, Hangzhou HuaAn Biotechnology, Catalog number R1501‐2, 1:250. DPPA3, Abcam, Catalog number ab19878, 1:250. FOXD3: Merck Millipore, Catalog number AB5687, 1:500. H3K9me3: crude serum, Antibody no 4861 (Jenuwein lab), 1:1,000.

### Immunoprecipitation

To determine the interaction between FOXD3 and SUV39H1/H2 and SETDB1, GFP‐SUV39H1 and GFP‐SUV39H2 expressing *Suv39h dn* ES cells and Foxd3 cKO cells expressing GFP‐FOXD3 expressing Foxd3 cKO cells were used. Cells were subjected to immunoprecipitation with GFP‐trap Dynabeads (Chromotek gtdk‐20) following the manufacturers' protocol. The eluted IP samples were immunoblotted with specific antibodies using established protocols (Bulut‐Karslioglu *et al*, 2012). The antibodies used for immunoblot are as follows: Foxd3: Merck Millipore, Catalog number AB5687, 1:500. GFP: Invitrogen, Catalog number A11122, 1:500. Setdb1: Thermo Fisher, Catalog number MA5‐15722, 1:500. Suv39h1: Sigma‐Aldrich (Merck), Catalog number 05‐615, 1:250.

### Foxd3 RNAi

Foxd3 siRNA (Catalog number L‐043570‐01‐0005) and scrambled non‐target siRNA (Catalog number D‐001810‐10‐05) were obtained from Dharmacon. *Suv39h dn* mESCs expressing GFP‐Suv39h1 cells were transfected with 25 nM siRNA (scrambled or Foxd3 si) with the DharmaFECT transfection reagent (Catalog number T‐2001‐01) using standard protocols. Cells were harvested 60 h after transfection for further experiments.

### STRING analysis

To determine the interaction profile of FOXD2, SUV39H1, SUV39H2, SETDB1 and SETDB2, these proteins were used as input in the “multiple proteins” window of the string.db algorithm (https://string‐db.org/) and *Mus musculus* was selected as the model organism. The interaction score was set to 0.150, and all the active interaction sources were selected to determine putative protein interactions based on text mining, co‐occurrence, experiments, databases, gene‐fusion, co‐expression or neighbourhood as depicted in Fig EV5A.

## Author contributions

DP conceptualised, designed and performed the experiments of the project. BK contributed to the experiments depicted in Figs 1B and 3A. BE and TM contributed to the experiments depicted in Fig 3C and E. MO‐S, DR and MS performed bioinformatics analysis. DP wrote the original draft.

## Conflict of interest

The authors declare that they have no conflict of interest.

## Supporting information



Expanded View Figures PDF

Table EV1

Table EV2

Dataset EV1

Dataset EV2

Source Data for Expanded View

Source Data for Figure 4

## Data Availability

The sequencing data generated and reported in this paper can be accessed in GEO using the accession number GSE173602. Link: https://www.ncbi.nlm.nih.gov/geo/query/acc.cgi?acc=GSE173602.

## References

[embr202153180-bib-0001] Biémont C (2010) A brief history of the status of transposable elements: from junk DNA to major players in evolution. Genetics 186: 1085–1093 21156958 10.1534/genetics.110.124180 PMC2998295

[embr202153180-bib-0002] Bourque G , Leong B , Vega VB , Chen X , Lee YL , Srinivasan KG , Chew J‐L , Ruan Y , Wei C‐L , Ng HH *et al* (2008) Evolution of the mammalian transcription factor binding repertoire via transposable elements. Genome Res 18: 1752–1762 18682548 10.1101/gr.080663.108 PMC2577865

[embr202153180-bib-0003] Bulut‐Karslioglu A , Perrera V , Scaranaro M , de la Rosa‐Velazquez IA , van de Nobelen S Shukeir N , Popow J , Gerle B , Opravil S , Pagani M *et al* (2012) A transcription factor‐based mechanism for mouse heterochromatin formation. Nat Struct Mol Biol 19: 1023–1030 22983563 10.1038/nsmb.2382

[embr202153180-bib-0004] Bulut‐Karslioglu A , De La Rosa‐Velázquez I , Ramirez F , Barenboim M , Onishi‐Seebacher M , Arand J , Galán C , Winter G , Engist B , Gerle B *et al* (2014a) Suv39h‐dependent H3K9me3 marks intact retrotransposons and silences LINE elements in mouse embryonic stem cells. Mol Cell 55: 277–290 24981170 10.1016/j.molcel.2014.05.029

[embr202153180-bib-0005] Bulut‐Karslioglu A , De La Rosa‐Velázquez IA , Ramirez F , Barenboim M , Onishi‐Seebacher M , Arand J , Galán C , Winter GE , Engist B , Gerle B , *et al* (2014b) Gene Expression Omnibus GSE57092 (https://www.ncbi.nlm.nih.gov/geo/query/acc.cgi?acc=GSE57092). [DATASET] 10.1016/j.molcel.2014.05.029 24981170

[embr202153180-bib-0006] Burton A , Torres‐Padilla M‐E (2014) Chromatin dynamics in the regulation of cell fate allocation during early embryogenesis. Nat Rev Mol Cell Biol 15: 723–734 25303116 10.1038/nrm3885

[embr202153180-bib-0007] Chen S , Zhou Y , Chen Y , Gu J (2018) fastp: an ultra‐fast all‐in‐one FASTQ preprocessor. Bioinformatics 34: i884–i890 30423086 10.1093/bioinformatics/bty560 PMC6129281

[embr202153180-bib-0008] Chen Z , Zhang Y (2019) Loss of DUX causes minor defects in zygotic genome activation and is compatible with mouse development. Nat Genet 51: 947–951 31133747 10.1038/s41588-019-0418-7 PMC6545155

[embr202153180-bib-0009] Clevidence DE , Overdier DG , Tao W , Qian X , Pani L , Lai E , Costa RH (1993) Identification of nine tissue‐specific transcription factors of the hepatocyte nuclear factor 3/forkhead DNA‐binding‐domain family. Proc Natl Acad Sci USA 90: 3948–3952 7683413 10.1073/pnas.90.9.3948 PMC46423

[embr202153180-bib-0010] Dang‐Nguyen TQ , Torres‐Padilla M‐E (2015) How cells build totipotency and pluripotency: nuclear, chromatin and transcriptional architecture. Curr Opin Cell Biol 34: 9–15 25935759 10.1016/j.ceb.2015.04.006

[embr202153180-bib-0011] Eckersley‐Maslin MA , Svensson V , Krueger C , Stubbs TM , Giehr P , Krueger F , Miragaia R , Kyriakopoulos C , Berrens R , Milagre I *et al* (2016) MERVL/Zscan4 network activation results in transient genome‐wide DNA demethylation of mESCs. Cell Rep 17: 179–192 27681430 10.1016/j.celrep.2016.08.087 PMC5055476

[embr202153180-bib-0012] Fan X , Zhang X , Wu X , Guo H , Hu Y , Tang F , Huang Y (2015a) Single‐cell RNA‐seq transcriptome analysis of linear and circular RNAs in mouse preimplantation embryos. Genome Biol 16: 148 26201400 10.1186/s13059-015-0706-1 PMC4511241

[embr202153180-bib-0013] Fan X , Zhang X , Wu X , Guo H , Hu Y , Tang F , Huang Y (2015b) Gene Expression Omnibus GSE53386 (https://www.ncbi.nlm.nih.gov/geo/query/acc.cgi?acc=GSE53386). [DATASET]

[embr202153180-bib-0014] Fort A , Hashimoto K , Yamada D , Salimullah MD , Keya CA , Saxena A , Bonetti A , Voineagu I , Bertin N , Kratz A *et al* (2014) Deep transcriptome profiling of mammalian stem cells supports a regulatory role for retrotransposons in pluripotency maintenance. Nat Genet 46: 558–566 24777452 10.1038/ng.2965

[embr202153180-bib-0015] Fu X , Djekidel MN , Zhang Y (2020) A transcriptional roadmap for 2C‐like–to–pluripotent state transition. Sci Adv 6: eaay5181 32523982 10.1126/sciadv.aay5181 PMC7259939

[embr202153180-bib-0016] Genet M , Torres‐Padilla M‐E (2020) The molecular and cellular features of 2‐cell‐like cells: a reference guide. Development 147: dev189688 32847823 10.1242/dev.189688

[embr202153180-bib-0017] Groh S , Schotta G (2017) Silencing of endogenous retroviruses by heterochromatin. Cell Mol Life Sci 74: 2055–2065 28160052 10.1007/s00018-017-2454-8 PMC11107624

[embr202153180-bib-0018] Guallar D , Pérez‐Palacios R , Climent M , Martínez‐Abadía I , Larraga A , Fernández‐Juan M , Vallejo C , Muniesa P , Schoorlemmer J (2012) Expression of endogenous retroviruses is negatively regulated by the pluripotency marker Rex1/Zfp42. Nucleic Acids Res 40: 8993–9007 22844087 10.1093/nar/gks686 PMC3467079

[embr202153180-bib-0019] Guo M , Zhang Y , Zhou J , Bi Y , Xu J , Xu CE , Kou X , Zhao Y , Li Y , Tu Z *et al* (2019) Precise temporal regulation of Dux is important for embryo development. Cell Res 29: 956–959 31591446 10.1038/s41422-019-0238-4 PMC6889123

[embr202153180-bib-0020] Hanna LA , Foreman RK , Tarasenko IA , Kessler DS , Labosky PA (2002) Requirement for Foxd3 in maintaining pluripotent cells of the early mouse embryo. Genes Dev 16: 2650–2661 12381664 10.1101/gad.1020502 PMC187464

[embr202153180-bib-0021] He J , Fu X , Zhang M , He F , Li W , Abdul MM , Zhou J , Sun LI , Chang C , Li Y *et al* (2019) Transposable elements are regulated by context‐specific patterns of chromatin marks in mouse embryonic stem cells. Nat Commun 10: 34 30604769 10.1038/s41467-018-08006-y PMC6318327

[embr202153180-bib-0022] Hendrickson PG , Doráis JA , Grow EJ , Whiddon JL , Lim J‐W , Wike CL , Weaver BD , Pflueger C , Emery BR , Wilcox AL *et al* (2017) Conserved roles of mouse DUX and human DUX4 in activating cleavage‐stage genes and MERVL/HERVL retrotransposons. Nat Genet 49: 925–934 28459457 10.1038/ng.3844 PMC5703070

[embr202153180-bib-0023] Hermant C , Torres‐Padilla M‐E (2021) TFs for TEs: the transcription factor repertoire of mammalian transposable elements. Genes Dev 35: 22–39 33397727 10.1101/gad.344473.120 PMC7778262

[embr202153180-bib-0024] Huang DW , Sherman BT , Lempicki RA (2009) Systematic and integrative analysis of large gene lists using DAVID bioinformatics resources. Nat Protoc 4: 44–57 19131956 10.1038/nprot.2008.211

[embr202153180-bib-0025] Iaco AD , Verp S , Offner S , Grun D , Trono D (2020) DUX is a non‐essential synchronizer of zygotic genome activation. Development 147: dev177725 31806660 10.1242/dev.177725 PMC7099940

[embr202153180-bib-0026] Ishiuchi T , Enriquez‐Gasca R , Mizutani E , Bošković A , Ziegler‐Birling C , Rodriguez‐Terrones D , Wakayama T , Vaquerizas JM , Torres‐Padilla M‐E (2015) Early embryonic‐like cells are induced by downregulating replication‐dependent chromatin assembly. Nat Struct Mol Biol 22: 662–671 26237512 10.1038/nsmb.3066

[embr202153180-bib-0027] Jachowicz JW , Bing X , Pontabry J , Bošković A , Rando OJ , Torres‐Padilla M‐E (2017) LINE‐1 activation after fertilization regulates global chromatin accessibility in the early mouse embryo. Nat Genet 49: 1502–1510 28846101 10.1038/ng.3945

[embr202153180-bib-0028] Jin Y , Tam OH , Paniagua E , Hammell M (2015) TEtranscripts: a package for including transposable elements in differential expression analysis of RNA‐seq datasets. Bioinformatics 31: 3593–3599 26206304 10.1093/bioinformatics/btv422 PMC4757950

[embr202153180-bib-0029] Jukam D , Shariati SAM , Skotheim JM (2017) Zygotic genome activation in vertebrates. Dev Cell 42: 316–332 28829942 10.1016/j.devcel.2017.07.026 PMC5714289

[embr202153180-bib-0030] Jurka J , Kapitonov VV , Pavlicek A , Klonowski P , Kohany O , Walichiewicz J (2005) Repbase update, a database of eukaryotic repetitive elements. Cytogenet Genome Res 110: 462–467 16093699 10.1159/000084979

[embr202153180-bib-0031] Karimi MM , Goyal P , Maksakova IA , Bilenky M , Leung D , Tang JX , Shinkai Y , Mager DL , Jones S , Hirst M *et al* (2011) DNA methylation and SETDB1/H3K9me3 regulate predominantly distinct sets of genes, retroelements and chimaeric transcripts in mouse ES cells. Cell Stem Cell 8: 676–687 21624812 10.1016/j.stem.2011.04.004 PMC3857791

[embr202153180-bib-0032] Kato Y , Kaneda M , Hata K , Kumaki K , Hisano M , Kohara Y , Okano M , Li E , Nozaki M , Sasaki H (2007) Role of the Dnmt3 family in de novo methylation of imprinted and repetitive sequences during male germ cell development in the mouse. Hum Mol Genet 16: 2272–2280 17616512 10.1093/hmg/ddm179

[embr202153180-bib-0033] Kim D , Pertea G , Trapnell C , Pimentel H , Kelley R , Salzberg SL (2013) TopHat2: accurate alignment of transcriptomes in the presence of insertions, deletions and gene fusions. Genome Biol 14: R36 23618408 10.1186/gb-2013-14-4-r36 PMC4053844

[embr202153180-bib-0034] Krishnakumar R , Chen AF , Pantovich MG , Danial M , Parchem RJ , Labosky PA , Blelloch R (2016a) FOXD3 regulates pluripotent stem cell potential by simultaneously initiating and repressing enhancer activity. Cell Stem Cell 18: 104–117 26748757 10.1016/j.stem.2015.10.003 PMC4775235

[embr202153180-bib-0035] Krishnakumar R , Chen AF , Pantovich MG , Danial M , Parchem RJ , Labosky PA , Blelloch R (2016b) Gene Expression Omnibus GSE58408 (https://www.ncbi.nlm.nih.gov/geo/query/acc.cgi?acc=GSE58408). [DATASET]

[embr202153180-bib-0036] Kunarso G , Chia N‐Y , Jeyakani J , Hwang C , Lu X , Chan Y‐S , Ng H‐H , Bourque G (2010) Transposable elements have rewired the core regulatory network of human embryonic stem cells. Nat Genet 42: 631–634 20526341 10.1038/ng.600

[embr202153180-bib-0037] Lam EW‐F , Brosens JJ , Gomes AR , Koo C‐Y (2013) Forkhead box proteins: tuning forks for transcriptional harmony. Nat Rev Cancer 13: 482–495 23792361 10.1038/nrc3539

[embr202153180-bib-0038] Langmead B , Salzberg SL (2012) Fast gapped‐read alignment with Bowtie 2. Nat Methods 9: 357–359 22388286 10.1038/nmeth.1923 PMC3322381

[embr202153180-bib-0039] Liu Y , Labosky PA (2008) Regulation of embryonic stem cell self‐renewal and pluripotency by Foxd3. Stem Cells 26: 2475–2484 18653770 10.1634/stemcells.2008-0269 PMC2658636

[embr202153180-bib-0040] Love MI , Huber W , Anders S (2014) Moderated estimation of fold change and dispersion for RNA‐seq data with DESeq2. Genome Biol 15: 550 25516281 10.1186/s13059-014-0550-8 PMC4302049

[embr202153180-bib-0041] Lukoseviciute M , Gavriouchkina D , Williams RM , Hochgreb‐Hagele T , Senanayake U , Chong‐Morrison V , Thongjuea S , Repapi E , Mead A , Sauka‐Spengler T (2018) From pioneer to repressor: bimodal foxd3 activity dynamically remodels neural crest regulatory landscape *in vivo* . Dev Cell 47: 608–628.e6 30513303 10.1016/j.devcel.2018.11.009 PMC6286384

[embr202153180-bib-0042] Macfarlan TS , Gifford WD , Agarwal S , Driscoll S , Lettieri K , Wang J , Andrews SE , Franco L , Rosenfeld MG , Ren B *et al* (2011a) Endogenous retroviruses and neighboring genes are coordinately repressed by LSD1/KDM1A. Genes Dev 25: 594–607 21357675 10.1101/gad.2008511 PMC3059833

[embr202153180-bib-0043] Macfarlan TS , Gifford WD , Agarwal S , Driscoll S , Lettieri K , Wang J , Andrews SE , Franco L , Rosenfeld MG , Ren B , *et al* (2011b) Gene Expression Omnibus GSE33923 (https://www.ncbi.nlm.nih.gov/geo/query/acc.cgi?acc=GSE33923). [DATASET] 10.1101/gad.2008511 PMC3059833 21357675

[embr202153180-bib-0044] Macfarlan TS , Gifford WD , Driscoll S , Lettieri K , Rowe HM , Bonanomi D , Firth A , Singer O , Trono D , Pfaff SL (2012) ES cell potency fluctuates with endogenous retrovirus activity. Nature 487: 57–63 22722858 10.1038/nature11244 PMC3395470

[embr202153180-bib-0045] Martens JH , O’Sullivan RJ , Braunschweig U , Opravil S , Radolf M , Steinlein P , Jenuwein T (2005) The profile of repeat‐associated histone lysine methylation states in the mouse epigenome. EMBO J 24: 800–812 15678104 10.1038/sj.emboj.7600545 PMC549616

[embr202153180-bib-0046] Messeguer X , Escudero R , Farré D , Núñez O , Martínez J , Albà MM (2002) PROMO: detection of known transcription regulatory elements using species‐tailored searches. Bioinformatics 18: 333–334 11847087 10.1093/bioinformatics/18.2.333

[embr202153180-bib-0047] Mikkelsen TS , Ku M , Jaffe DB , Issac B , Lieberman E , Giannoukos G , Alvarez P , Brockman W , Kim T‐K , Koche RP *et al* (2007) Genome‐wide maps of chromatin state in pluripotent and lineage‐committed cells. Nature 448: 553–560 17603471 10.1038/nature06008 PMC2921165

[embr202153180-bib-0048] Percharde M , Lin C‐J , Yin Y , Guan J , Peixoto GA , Bulut‐Karslioglu A , Biechele S , Huang B , Shen X , Ramalho‐Santos M (2018) A LINE1‐nucleolin partnership regulates early development and ESC identity. Cell 174: 391–405.e19 29937225 10.1016/j.cell.2018.05.043 PMC6046266

[embr202153180-bib-0049] Peters AHFM , O'Carroll D , Scherthan H , Mechtler K , Sauer S , Schöfer C , Weipoltshammer K , Pagani M , Lachner M , Kohlmaier A *et al* (2001) Loss of the Suv39h histone methyltransferases impairs mammalian heterochromatin and genome stability. Cell 107: 323–337 11701123 10.1016/s0092-8674(01)00542-6

[embr202153180-bib-0050] Peters AHFM , Kubicek S , Mechtler K , O'Sullivan RJ , Derijck AAHA , Perez‐Burgos L , Kohlmaier A , Opravil S , Tachibana M , Shinkai Y *et al* (2003) Partitioning and plasticity of repressive histone methylation states in mammalian chromatin. Mol Cell 12: 1577–1589 14690609 10.1016/s1097-2765(03)00477-5

[embr202153180-bib-0051] Plank JL , Suflita MT , Galindo CL , Labosky PA (2014) Transcriptional targets of Foxd3 in murine ES cells. Stem Cell Res 12: 233–240 24270162 10.1016/j.scr.2013.10.008 PMC3934354

[embr202153180-bib-0052] Probst AV , Okamoto I , Casanova M , El Marjou F , Le Baccon P , Almouzni G (2010) A strand‐specific burst in transcription of pericentric satellites is required for chromocenter formation and early mouse development. Dev Cell 19: 625–638 20951352 10.1016/j.devcel.2010.09.002

[embr202153180-bib-0053] Ramírez F , Dündar F , Diehl S , Grüning BA , Manke T (2014) deepTools: a flexible platform for exploring deep‐sequencing data. Nucleic Acids Res 42: W187–W191 24799436 10.1093/nar/gku365 PMC4086134

[embr202153180-bib-0054] Respuela P , Nikolić M , Tan M , Frommolt P , Zhao Y , Wysocka J , Rada‐Iglesias A (2016) Foxd3 promotes exit from naive pluripotency through enhancer decommissioning and inhibits germline specification. Cell Stem Cell 18: 118–133 26748758 10.1016/j.stem.2015.09.010 PMC5048917

[embr202153180-bib-0055] Robinson JT , Thorvaldsdóttir H , Winckler W , Guttman M , Lander ES , Getz G , Mesirov JP (2011) Integrative genomics viewer. Nat Biotechnol 29: 24–26 21221095 10.1038/nbt.1754 PMC3346182

[embr202153180-bib-0056] Shi H , Strogantsev R , Takahashi N , Kazachenka A , Lorincz MC , Hemberger M , Ferguson‐Smith AC (2019) ZFP57 regulation of transposable elements and gene expression within and beyond imprinted domains. Epigenetics Chromatin 12: 49 31399135 10.1186/s13072-019-0295-4 PMC6688207

[embr202153180-bib-0057] Sweet DJ (2016) Foxd3: a repressor, an activator, or both? Cell Stem Cell 18: 1–2 26748746 10.1016/j.stem.2015.12.009

[embr202153180-bib-0058] Szklarczyk D , Gable AL , Lyon D , Junge A , Wyder S , Huerta‐Cepas J , Simonovic M , Doncheva NT , Morris JH , Bork P *et al* (2019) STRING v11: protein‐protein association networks with increased coverage, supporting functional discovery in genome‐wide experimental datasets. Nucleic Acids Res 47: D607–D613 30476243 10.1093/nar/gky1131 PMC6323986

[embr202153180-bib-0059] Treangen TJ , Salzberg SL (2012) Repetitive DNA and next‐generation sequencing: computational challenges and solutions. Nat Rev Genet 13: 36–46 10.1038/nrg3117 PMC3324860 22124482

[embr202153180-bib-0060] Velazquez Camacho O , Galan C , Swist‐Rosowska K , Ching R , Gamalinda M , Karabiber F , De La Rosa‐Velazquez I , Engist B , Koschorz B , Shukeir N *et al* (2017) Major satellite repeat RNA stabilize heterochromatin retention of Suv39h enzymes by RNA‐nucleosome association and RNA:DNA hybrid formation. eLife 6: e25293 28760199 10.7554/eLife.25293 PMC5538826

[embr202153180-bib-0061] Wolf G , de Iaco A , Sun M‐A , Bruno M , Tinkham M , Hoang D , Mitra A , Ralls S , Trono D , Macfarlan TS (2020) KRAB‐zinc finger protein gene expansion in response to active retrotransposons in the murine lineage. eLife 9: e56337 32479262 10.7554/eLife.56337 PMC7289599

[embr202153180-bib-0062] Wu K , Liu HE , Wang Y , He J , Xu S , Chen Y , Kuang J , Liu J , Guo L , Li D *et al* (2020) SETDB1‐mediated cell fate transition between 2C‐like and pluripotent states. Cell Rep 30: 25–36.e6 31914391 10.1016/j.celrep.2019.12.010

[embr202153180-bib-0063] Yang F , Huang X , Zang R , Chen J , Fidalgo M , Sanchez‐Priego C , Yang J , Caichen A , Ma F , Macfarlan T *et al* (2020) DUX‐miR‐344‐ZMYM2‐mediated activation of MERVL LTRs induces a totipotent 2C‐like state. Cell Stem Cell 26: 234–250.e7 32032525 10.1016/j.stem.2020.01.004 PMC8074926

[embr202153180-bib-0064] Zaret KS , Carroll JS (2011) Pioneer transcription factors: establishing competence for gene expression. Genes Dev 25: 2227–2241 22056668 10.1101/gad.176826.111 PMC3219227

[embr202153180-bib-0065] Zhang W , Chen F , Chen R , Xie D , Yang J , Zhao X , Guo R , Zhang Y , Shen Y , Göke J *et al* (2019) Zscan4c activates endogenous retrovirus MERVL and cleavage embryo genes. Nucleic Acids Res 47: 8485–8501 31304534 10.1093/nar/gkz594 PMC7145578

