# Supplementary material for: Foxd3 controls heterochromatin‐mediated repression of repeat elements and 2‐cell state transcription
Source: EMBO Rep. 2021 Oct 4;22(12):e53180. doi: 10.15252/embr.202153180 (PMC8647145; doi:10.15252/embr.202153180)
Supplement: Supplementary file 1 — Expanded View Figures PDF [file EMBR-22-e53180-s003.pdf]

Expanded View Figures

Figure EV1. FOXD3 binds to MERVL and MSR elements.

A Consensus sequences for MSR, MERVL-LTR, MERVL-int, L1MdA, L1MdGf, L1MdTf and L1MdF marked with TF-binding sites predicted by PROMO.

B Electrophoretic mobility shift assay (EMSA) with increasing concentrations (0, 15 nM, 30 nM, 60 nM, 1.25 μM and 2.5 μM) of GST-FOXD3 and fixed concentration (50 nM) of 5′-Cy5-labelled dsDNA oligonucleotides (35 bp each) from MERVL-LTR, mutated MERVL-LTR and mutated MSR sequences. The experiment was repeated 3 times.

C Metadata profile showing enrichment of FOXD3 over the MSR, MERVL and MT2_Mm consensus sequence including ± 2 kb flanking the FOXD3 peak on each repeat. *Y*-axes represent relative signal intensity.

D FOXD3 enrichment detected by FLAG-FOXD3 ChIP-Seq on representative MSR, MERVL and L1MdA loci.

E Schematic (not to scale) representing MSR, MERVL and LINE sequences depicting FOXD3-binding sites and primers used for qPCR.

F ChIP-qPCR enrichment of FOXD3 in mESCs using primers specific for LINE sequences. *Sox15* promoter primers are used as positive control. Data are represented as percentage of input, and the average of three biological replicates is plotted. Error bar indicates standard error of the mean (SEM). Asterisks indicate statistically significant differences compared with no antibody control levels (*$P < 0.05$, paired *t*-test).

© 2021 The Authors

© 2021 The Authors

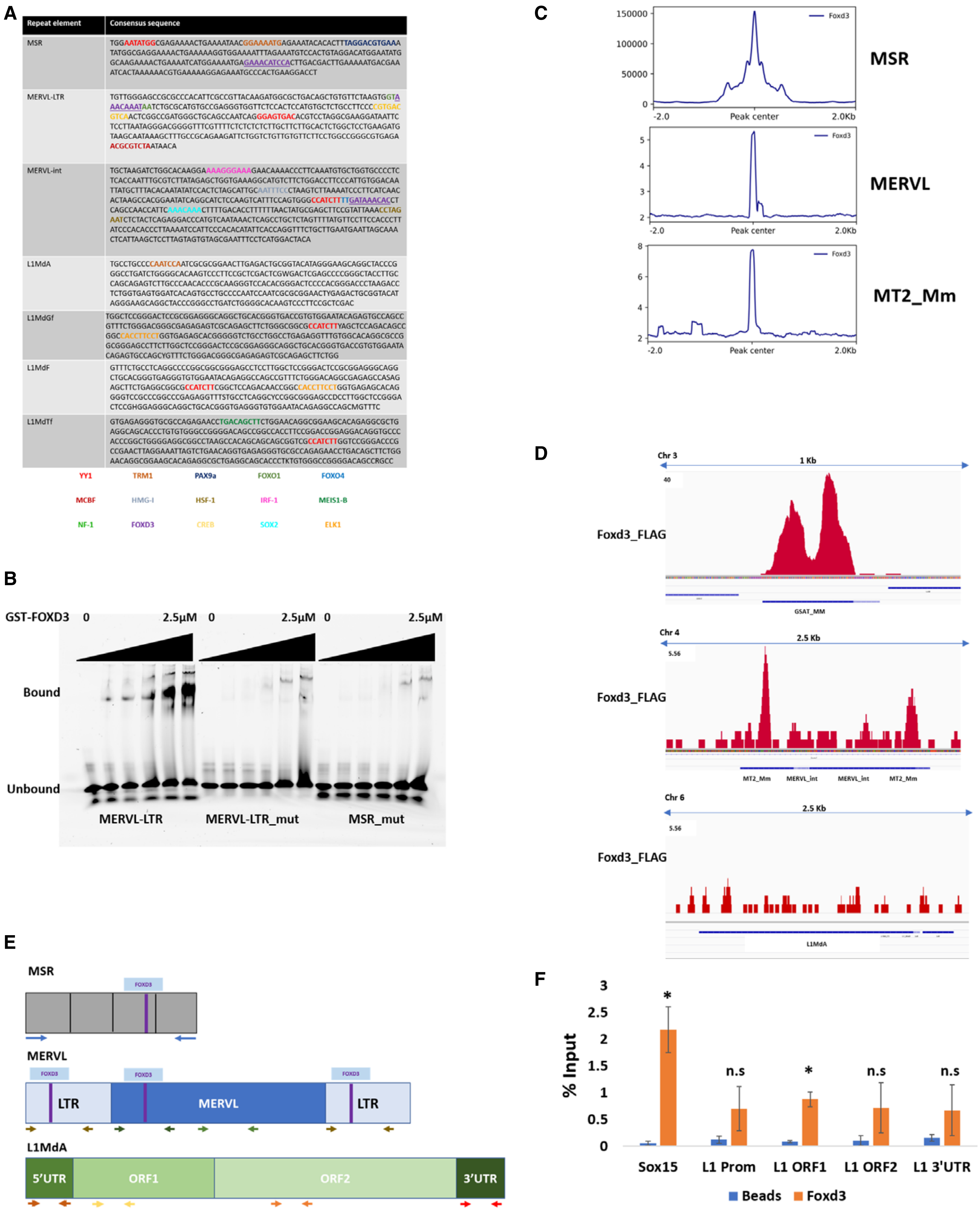

Figure EV1.

© 2021 The Authors
16 of 14 EMBO reports 22: e53180 | 2021 © 2021 The Authors

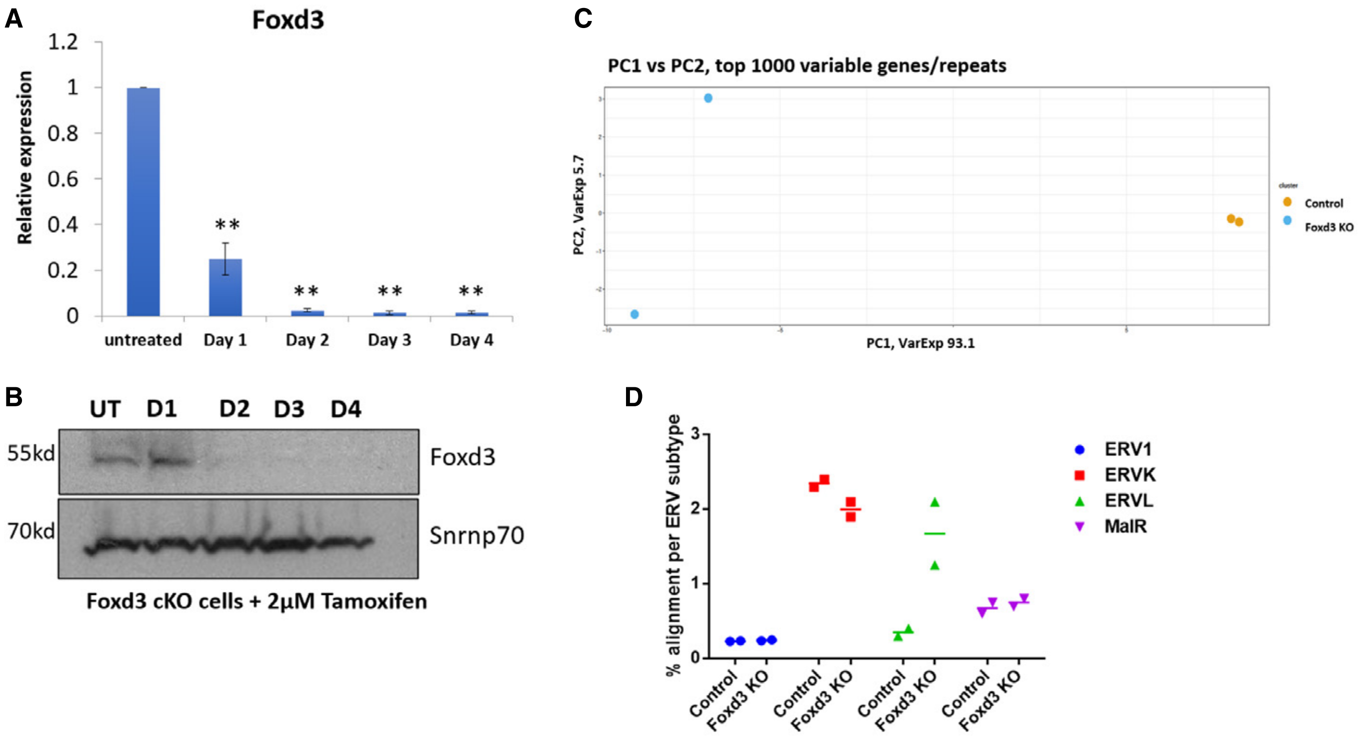

Figure EV2. MERVL is upregulated in Foxd3 KO cells.

A RT–qPCR analysis of Foxd3 in control and Foxd3 KO cells. The data are plotted as average fold change relative to control, after normalisation to *Gapdh*. Error bars indicate SEM ($N = 3$ biological replicates). Asterisks indicate statistically significant differences compared with untreated cells (**$P < 0.005$, paired t-test).

B Immunoblot of protein lysates prepared from untreated and 4-OHT treated cells, probed with Foxd3 antibody. Snrnp70 is used as loading control. The experiment was repeated three times using biological replicates.

C Principal Component Analysis of the RNA-Seq data from control (Orange) and Foxd3 KO (Blue) indicating percentage variance for two principal components.

D Expression levels for each ERV subtype plotted for control and Foxd3 KO cells. *Y*-axis represents percentage alignment for each repeat subtype from RNA-Seq data. Individual data points from two biological replicates are plotted.

Source data are available online for this figure.

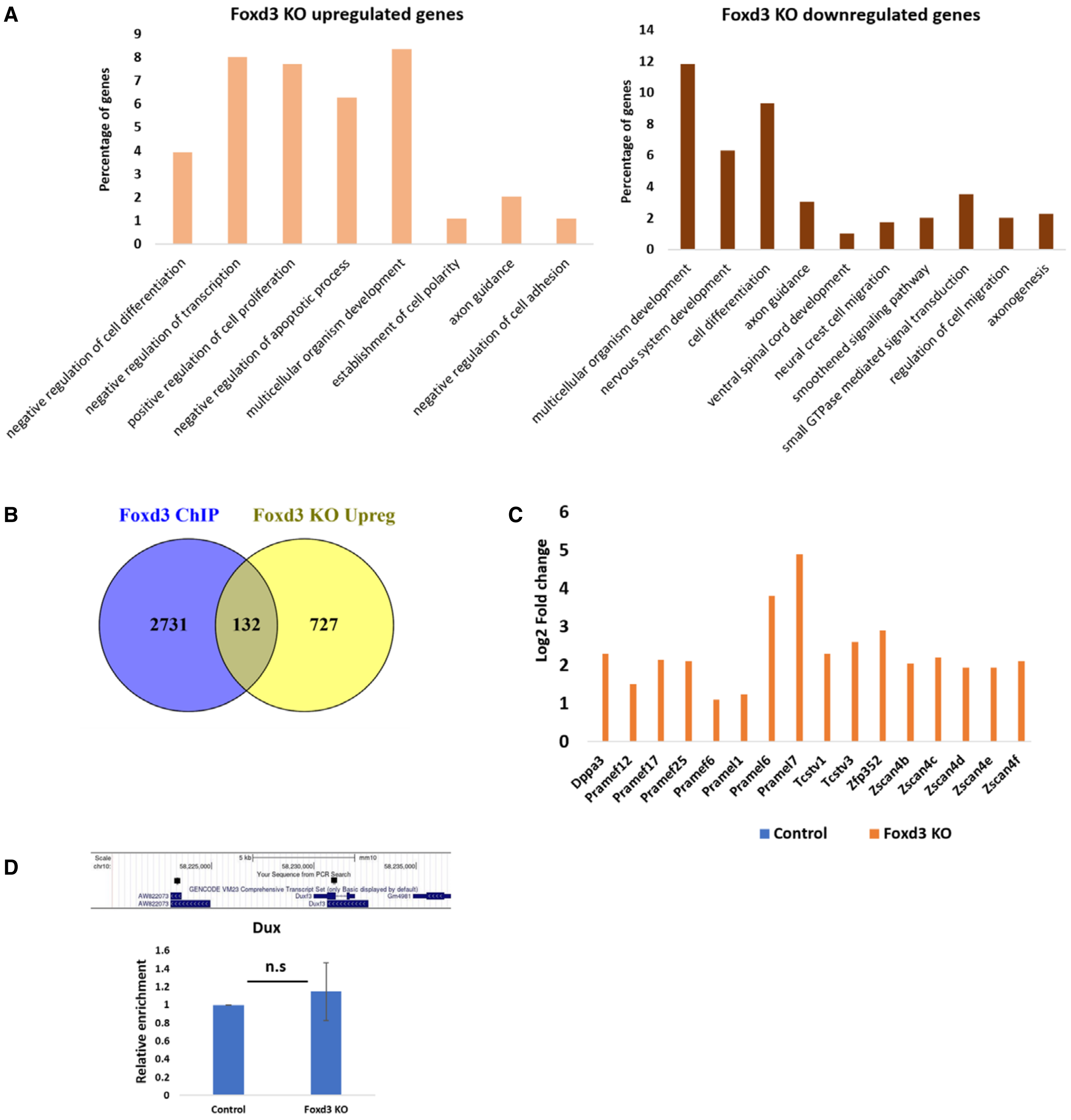

Figure EV3. 2CLC genes are upregulated in Foxd3 KO cells.

A GO protein classes significantly enriched in genes upregulated (left panel) and downregulated (right panel) in Foxd3 KO cells. The *Y*-axis represents percentage of genes belonging to each protein class.

B Venn diagram comparing Foxd3 bound genes (Krishnakumar *et al*, 2016) with genes upregulated in Foxd3 KO cells.

C Expression of representative 2CLC genes in Foxd3 KO cells. The *Y*-axis represents $\log_2$ fold change in Foxd3 KO cells compared with control cells.

D Schematic for RT–qPCR primers specific for Duxf3 and AW822073 (black squares). RT–qPCR analysis depicting Dux expression in control and Foxd3 KO cells. The data are plotted as average fold change relative to control, after normalisation to *Gapdh*. Error bars indicate SEM (n = 3 biological replicates). Statistical test: paired t-test.

 © 2021 The Authors

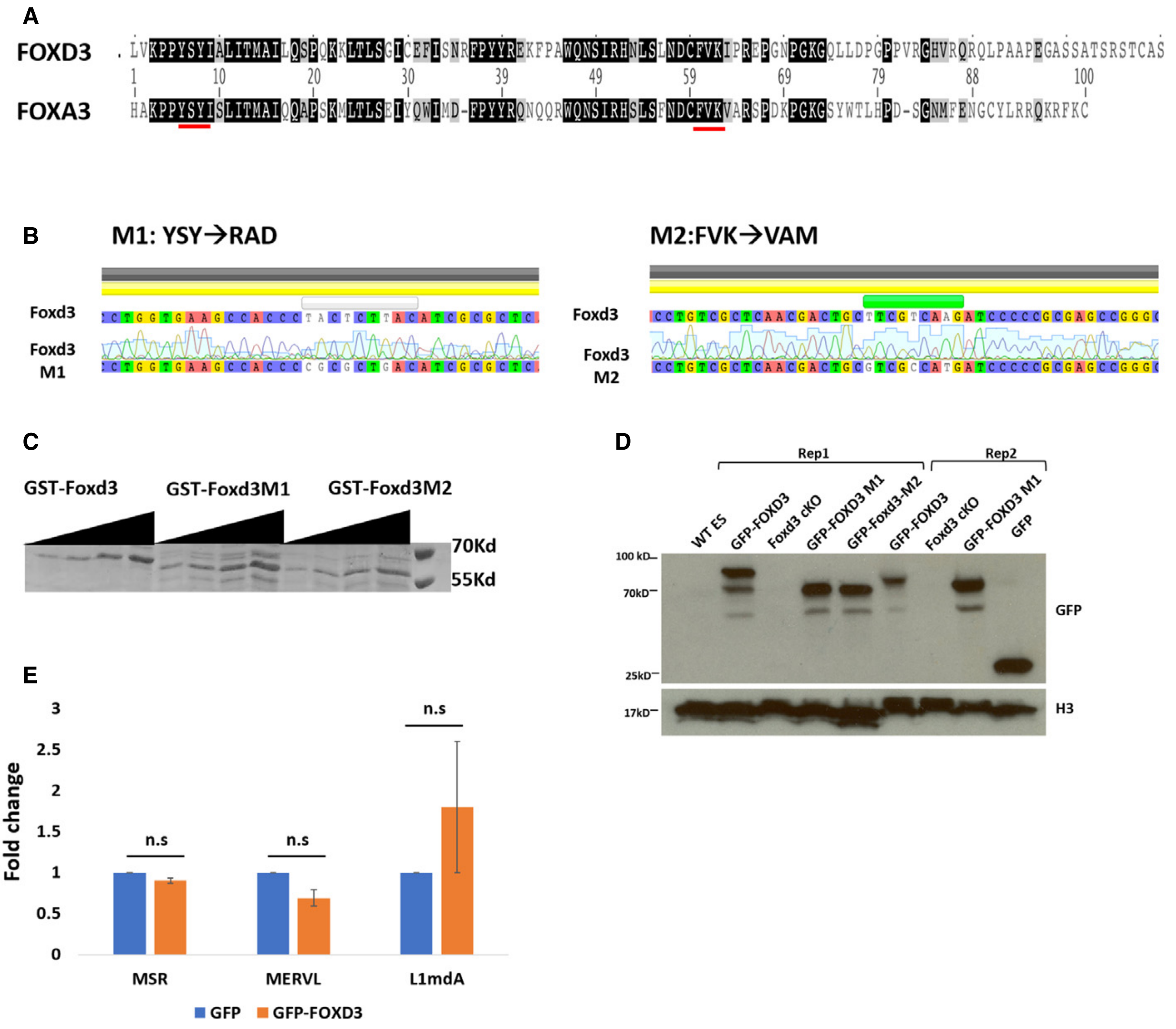

Figure EV4. Establishment of mutant FOXD3 constructs and cell lines.

A Conservation of YSY and FVK amino acids (underlined) between mouse FOXA3 and FOXD3 DNA-binding domain sequences.

B DNA sequencing results indicating generation of Foxd3 M1 and M2 mutants.

C Coomassie staining indicating expression of recombinant GST-FOXD3, GST-FOXD3 M1 and GST-FOXD3 M2. The experiment was repeated three times using biological replicates.

D Immunoblot (α GFP top panel and α H3 bottom panel) for lysates from Foxd3 cKO cells expressing GFP-FOXD3, GFP-FOXD3 M1 and GFP-FOXD3 M2. Histone H3 levels are used as loading control. The experiment was repeated three times using biological replicates.

E RT–qPCR analysis depicting MSR, MERVL and L1MdA expression in control and GFP-FOXD3 overexpressing Foxd3 cKO cells. The data are plotted as average fold change relative to control, after normalisation to *Gapdh*. Error bars indicate SEM (n = 3 biological replicates). Statistical test: paired t-test.

Source data are available online for this figure.

© 2021 The Authors

Figure EV5. Role of FOXD3 in SUV39H1-mediated regulation of MSR and MERVL.

A STRING analysis to predict putative interaction between FOXD3, SUV39H1, SUV39H2, SETDB1 and SETDB2. FOXD3 was significantly predicted to interact with SUV39H1 and SETDB1 (P-value < 0.01). The criteria used for predicting interaction are depicted.

B GFP immunoprecipitation analysis using Foxd3 cKO mESCs expressing GFP-FOXD3. Immunoblot results using antibodies against SUV39H1, SETDB1 and GFP are depicted. The experiment was repeated three times using biological replicates.

C Volcano plot depicting significantly upregulated and downregulated repeats in *Suv39h dn* ES cells compared with wild-type ES cells determined by RNA-Seq analysis of nuclear RNA. MSR (GSAT_MM), MERVL-int, L1MdA and MT2_Mm are labelled.

D ChIP-qPCR depicting enrichment of H3K4me3 (top panel) and H3K27me3 (bottom panel) over MERVL, MSR and L1MdA in control and Foxd3 KO cells. *Actb* promoter is used as negative control. Data are represented as average percentage input of 3 biological replicates. Error bars represent SEM (*P < 0.05 paired t-test).

E Schematic depicting the location of Foxd3 siRNAs (pink) along the Foxd3 locus (upper panel). Immunofluorescence detected by FOXD3 antibody in Foxd3i cells. Nuclei are stained with DAPI. Scale bar = 10 μm (lower left). RT–qPCR analysis of Foxd3 expression in control and Foxd3i cells. Data are represented as fold change compared with control (scrambled) after normalisation to *Gapdh*. N = 3 biological replicates (lower right). Error bars represent SEM. (*P < 0.05 paired t-test).

F Bar graph depicting Foxd3 expression at different stages of embryonic development. Y-axis depicts average FPKM from 4 independent single cell RNA-Seq data sets. Error bar represents standard deviation. (*P < 0.05, **P < 0.005, unpaired t-test).

G Bar graph depicting relative Foxd3 expression in MERVL-positive and MERVL-negative cells. The data are represented as the average read counts obtained from RNA-Seq data from three biological replicates. Error bars represent SEM. Statistical test: unpaired t-test.

Source data are available online for this figure.

 © 2021 The Authors

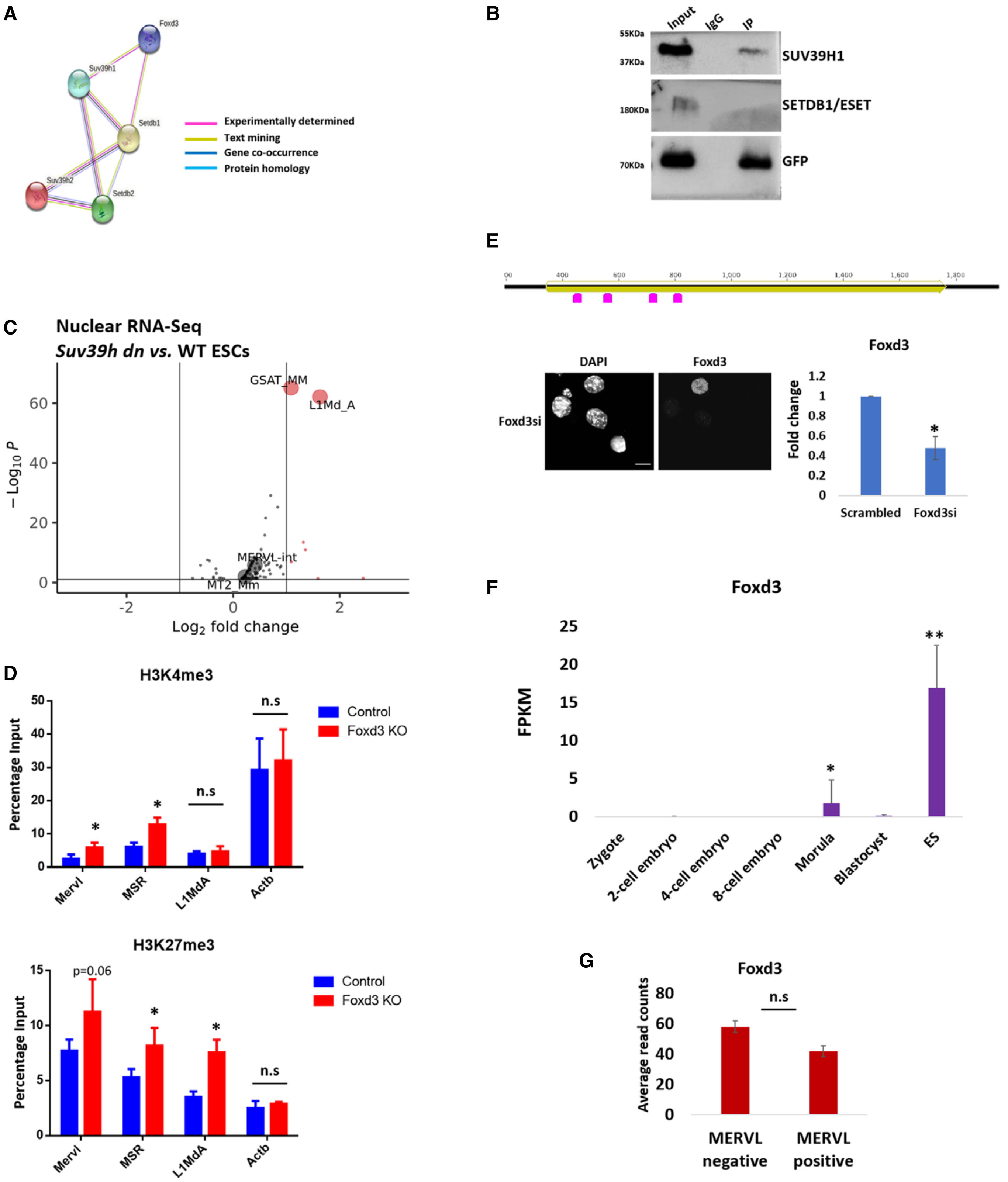
A
Foxd3
Suv39h1
Setdb1
Suv39h2
Setdb2
Experimentally determined
Text mining
Gene co-occurrence
Protein homology
B
Input
IgG
IP
55KDa
37KDa
SUV39H1
180KDa
SETDB1/ESET
70KDa
GFP
E
Foxd3
DAPI
Foxd3
Foxd3si
Fold change
Scrambled
Foxd3si
C
Nuclear RNA-Seq
Suv39h dn vs. WT ESCs
GSAT_MM
L1Md_A
MERVL-int
MT2_Mm
− Log10 P
Log2 fold change
D
H3K4me3
Percentage Input
Control
Foxd3 KO
n.s
Mervl
MSR
L1MdA
Actb
H3K27me3
p=0.06
F
Foxd3
FPKM
Zygote
2-cell embryo
4-cell embryo
8-cell embryo
Morula
Blastocyst
ES
G
Foxd3
Average read counts
MERVL negative
MERVL positive

Figure EV5.
