# Supplementary material for: Foxd3 controls heterochromatin‐mediated repression of repeat elements and 2‐cell state transcription
Source: EMBO Rep. 2021 Oct 4;22(12):e53180. doi: 10.15252/embr.202153180 (PMC8647145; doi:10.15252/embr.202153180)
Supplement: Supplementary file 2 — Table EV1 [file EMBR-22-e53180-s006.docx]

| 0610040J01Rik | Fam81a | Lnx1 | Rapgefl1 |
| --- | --- | --- | --- |
| 2010107G12Rik | Fbxo15 | Lonrf1 | Rbms3 |
| 4930506M07Rik | Fgfbp1 | Lpar1 | Rcor2 |
| 6430573F11Rik | Flnb | Lrpap1 | Rcsd1 |
| Abca4 | Frmpd1 | Lxn | Rimkla |
| Abcb5 | Fundc1 | Mfge8 | Robo3 |
| Adamts7 | Gabra1 | Mobp | Ror1 |
| Adamts8 | Gad2 | Mpped2 | Rragd |
| Aebp2 | Galnt3 | Mreg | S1pr1 |
| Alpl | Gata6 | Nabp1 | Satb2 |
| Alppl2 | Gjb3 | Nanos3 | Sh2d4b |
| Ankrd45 | Glis3 | Nat1 | Slc25a4 |
| Aox3 | Gpd1 | Nelfa | Slc7a9 |
| Ap3b2 | Gprc5c | Ngfr | Slco2a1 |
| Aqp3 | Gprin3 | Nptx2 | Sorbs1 |
| B020004J07Rik | Gsta3 | Nrp2 | Sox21 |
| Bcl2l14 | Hmga2 | Nt5e | Spesp1 |
| Cadps2 | Hmgn5 | Nudt4 | Spic |
| Cbln1 | Id2 | Oasl2 | Tbc1d8 |
| Ccdc17 | Id3 | Otud1 | Tbx3 |
| Ccdc60 | Id4 | Otx2 | Tfap2c |
| Clcn5 | Igf2bp2 | Pcdh19 | Ticrr |
| Col5a2 | Il6ra | Peg12 | Tmcc3 |
| Cpsf4l | Inpp4b | Phf13 | Tmem132c |
| Csf3r | Ipmk | Pkd1l1 | Trpc6 |
| Ctnnal1 | Kcnn2 | Plekhg1 | Ulk1 |
| Dlgap2 | Kifc3 | Podxl | Urgcp |
| Dppa2 | Klf2 | Popdc3 | Veph1 |
| Dppa3 | Lax1 | Pou6f2 | Wtap |
| Dysf | Lbh | Pramel6 | Yes1 |
| Egfl6 | Lmo7 | Prep | Zbtb10 |
| Eomes | Lmx1a | Prickle1 | Zdhhc23 |
| Eps8l2 | Lmx1b | Rab11fip4 | Zfp560 |

**Table EV1: Foxd3 targets upregulated in Foxd3 KO cells**
