# Supplementary material for: Foxd3 controls heterochromatin‐mediated repression of repeat elements and 2‐cell state transcription
Source: EMBO Rep. 2021 Oct 4;22(12):e53180. doi: 10.15252/embr.202153180 (PMC8647145; doi:10.15252/embr.202153180)
Supplement: Supplementary file 3 — Table EV2 [file EMBR-22-e53180-s005.docx]

**Table EV2: Primers used in the study**

| **NAME** | **SEQUENCE** | **EXPERIMENT** |
| --- | --- | --- |
| MERVL-LTR | CAGCTGTGTTCTAAGTGGTAAACAAATAATCTGCG | EMSA |
| MERVL-LTR_MUT | CAGCTGTGTTCTAAGTGGTACACGCCTAATCTGCG | EMSA |
| MERVL-INT | CCATCTTTTGATAAACACCTCAGCCAACCATTCAA | EMSA |
| MSR | CTGAAAATCATGGAAAATGAGAAACATCCACTTGA | EMSA |
| MSR_MUT | CTGAAAATCATGGAAAATGCGTGACGGCGACTTGA | EMSA |
| SOX15 | CAGAGGCTACTCTGAAACAAATAAAGAGATATAAA | EMSA |
| L1MDA | GTACATAGGGAAGCAGGCTACCCGGGCCTGATCTG | EMSA |
| HPRT | AGGGCGGGCCGAGGGGCGGAGCCTGGCCGGCAGCG | EMSA |
|  |  |  |
|  |  |  |
| MAJOR SAT - F | TGGAATATGGCGAGAAAACTG | RT-QPCR, CHIP |
| MAJOR SAT - R | AGGTCCTTCAGTGGGCATTT | RT-QPCR, CHIP |
| MERVL-F | TTTCTCAAGGCCCACCAATAGT | RT-QPCR, CHIP |
| MERVL-R | GACACCTTTTTTAACTATGCGAGC | RT-QPCR, CHIP |
| MERVL-INTF1 | TGCTAAGATCTGGCACAAGG | RT-QPCR, CHIP |
| MERVL-INTR1 | CTGGACCTTCCCATTGTGGA | RT-QPCR, CHIP |
| MERVL-INTF2 | GATAAACACCTCAGCCAACC | RT-QPCR, CHIP |
| MERVL-INTR2 | CTCAGAGGACCCATGTCAAT | RT-QPCR, CHIP |
| L1_PROMOTER - F | ACTGCGGTACATAGGGAAGC | RT-QPCR, CHIP |
| L1_PROMOTER - R | TGTGATCCACTCACCAGAGG | RT-QPCR, CHIP |
| L1_ORF1 - F | CACTCCCACCCCACCTAGT | RT-QPCR, CHIP |
| L1_ORF1 - R | TAACTCTTTAGCAGTGCTCTCCTGT | RT-QPCR, CHIP |
| L1_ORF2 - F | ACCTGGACGAAATGGACAAA | RT-QPCR, CHIP |
| L1_ORF2 - R | CATCTGGTCCTGGGCTTTT | RT-QPCR, CHIP |
| L1_3'UTR - F | CCAGCAAACACAGAAGTGGA | RT-QPCR, CHIP |
| L1_3'UTR - R | CCGACTAGGCCATCTTTTGA | RT-QPCR, CHIP |
| SOX15_PROMOTER-F | GGTGCTTGAGAATTGAGACA | CHIP |
| SOX15_PROMOTER-R | GTCTTGTTCTTCCCAGTCCT | CHIP |
| ACTB-F | AGCCAACTTTACGCCTAGCGT | CHIP |
| ACTB-R | TCTCAAGATGGACCTAATACGGC | CHIP |
| FOXD3-F | GTCCGCTGGGAATAACTTTCCGTA | RT-QPCR |
| FOXD3-R | ATGTACAAAGAATGTCCCTCCCACCC | RT-QPCR |
| IAP-F | GCACCCTCAAAGCCTATCTTA | RT-QPCR |
| IAP-R | TCCCTTGGTCAGTCTGGATTT | RT-QPCR |
| ZSCAN4-F | CCTCCCTGGGCTTCTTGGCAT | RT-QPCR |
| ZSCAN4-R | AGCTGCCAACCAGAAAGACACTGT | RT-QPCR |
| DPPA3-F | CGGGGTTTAGGGTTAGCTTT | RT-QPCR |
| DPPA3-R | GGACCCTGAAACTCCTCAGA | RT-QPCR |
| DUX_FWD | AAAGGAAGAGCATGTGCCAGC | RT-QPCR |
| DUX_REV | GCAGTAAGCTGTCCTGGGAAC | RT-QPCR |
