# Supplementary figures and images for: Foxd3 controls heterochromatin‐mediated repression of repeat elements and 2‐cell state transcription
Source: EMBO Rep. 2021 Oct 4;22(12):e53180. doi: 10.15252/embr.202153180 (PMC8647145; doi:10.15252/embr.202153180)

Figure EV5 b

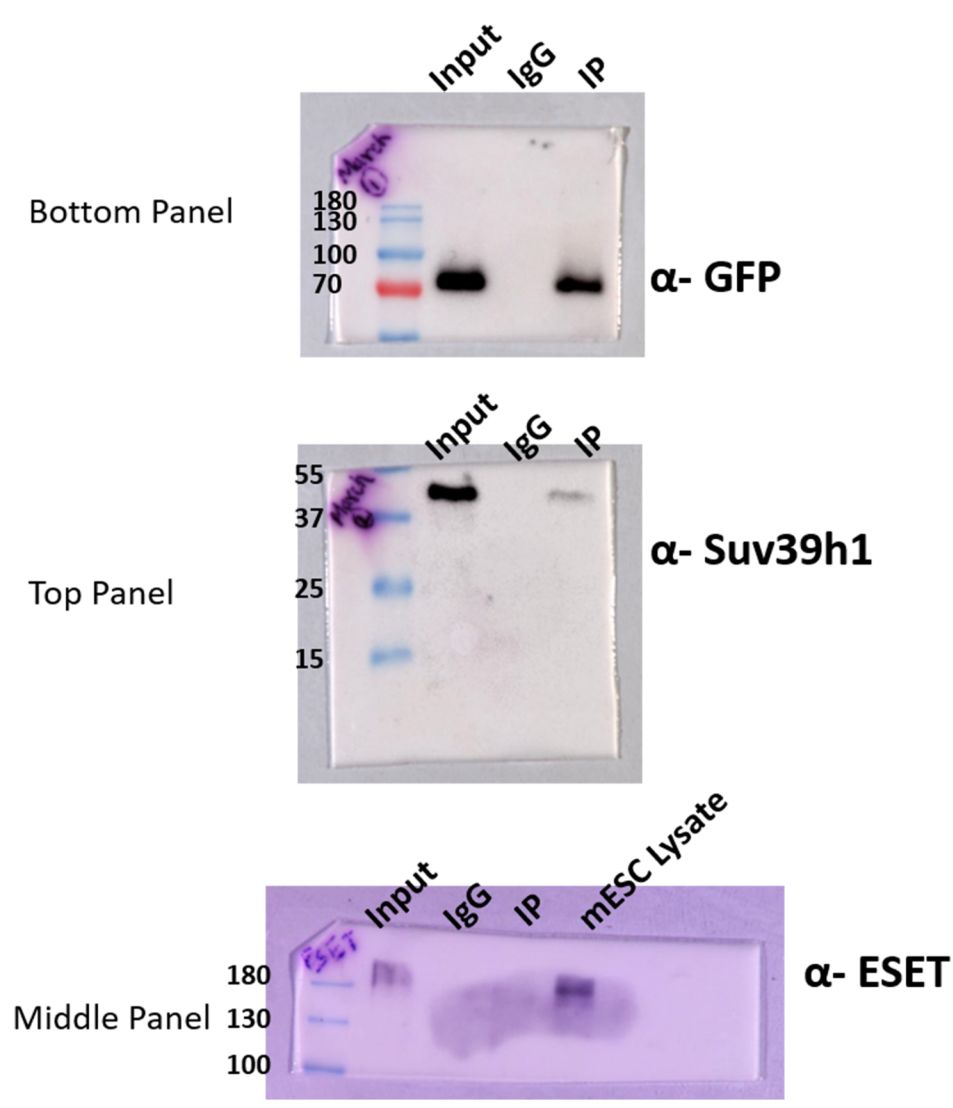

Supplement: Supplementary file 6 — Source Data for Expanded View [file EMBR-22-e53180-s004.zip › EV_Figure_Source_Data/Figure_Source_Data_EV5B-sd.pdf]

Figure EV4 c

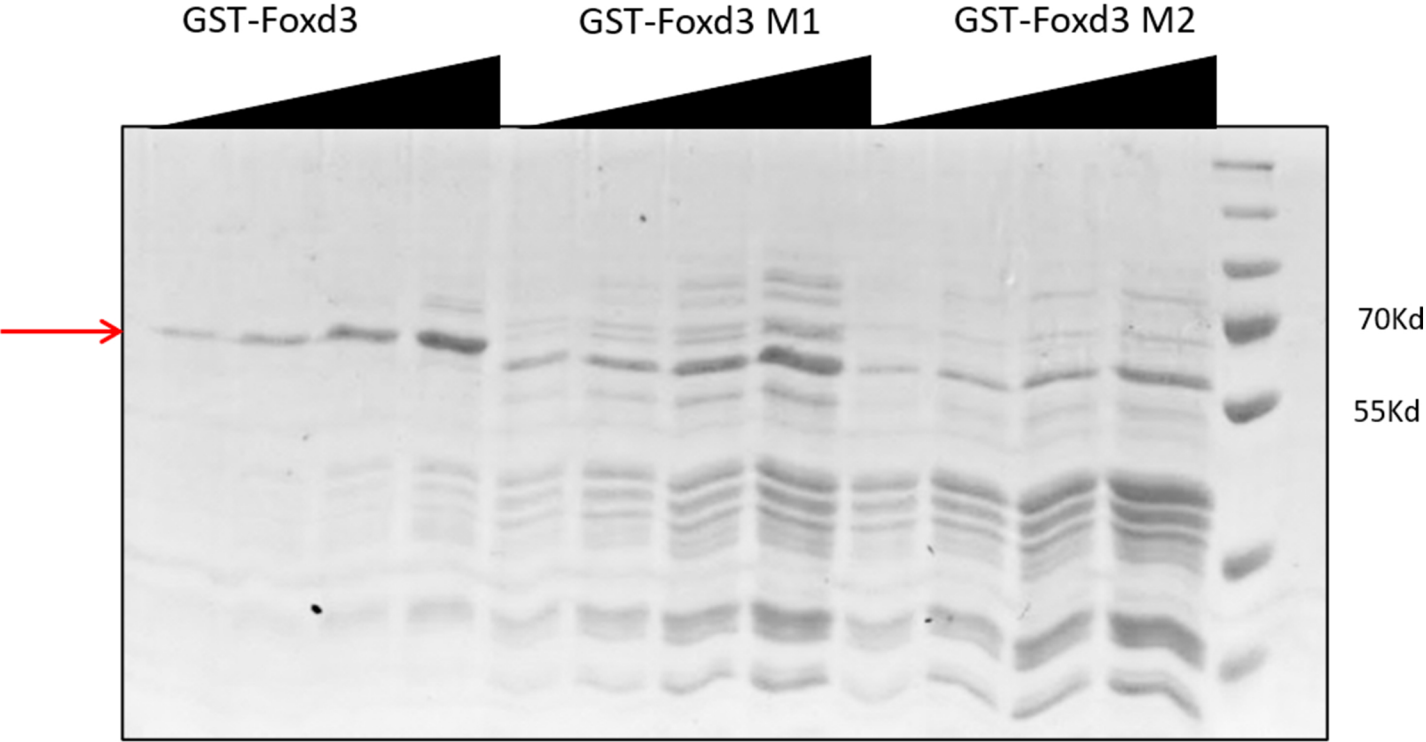

Supplement: Supplementary file 6 — Source Data for Expanded View [file EMBR-22-e53180-s004.zip › EV_Figure_Source_Data/Figure_Source_Data_Figure_EV4C-sd.pdf]

Figure EV2 b

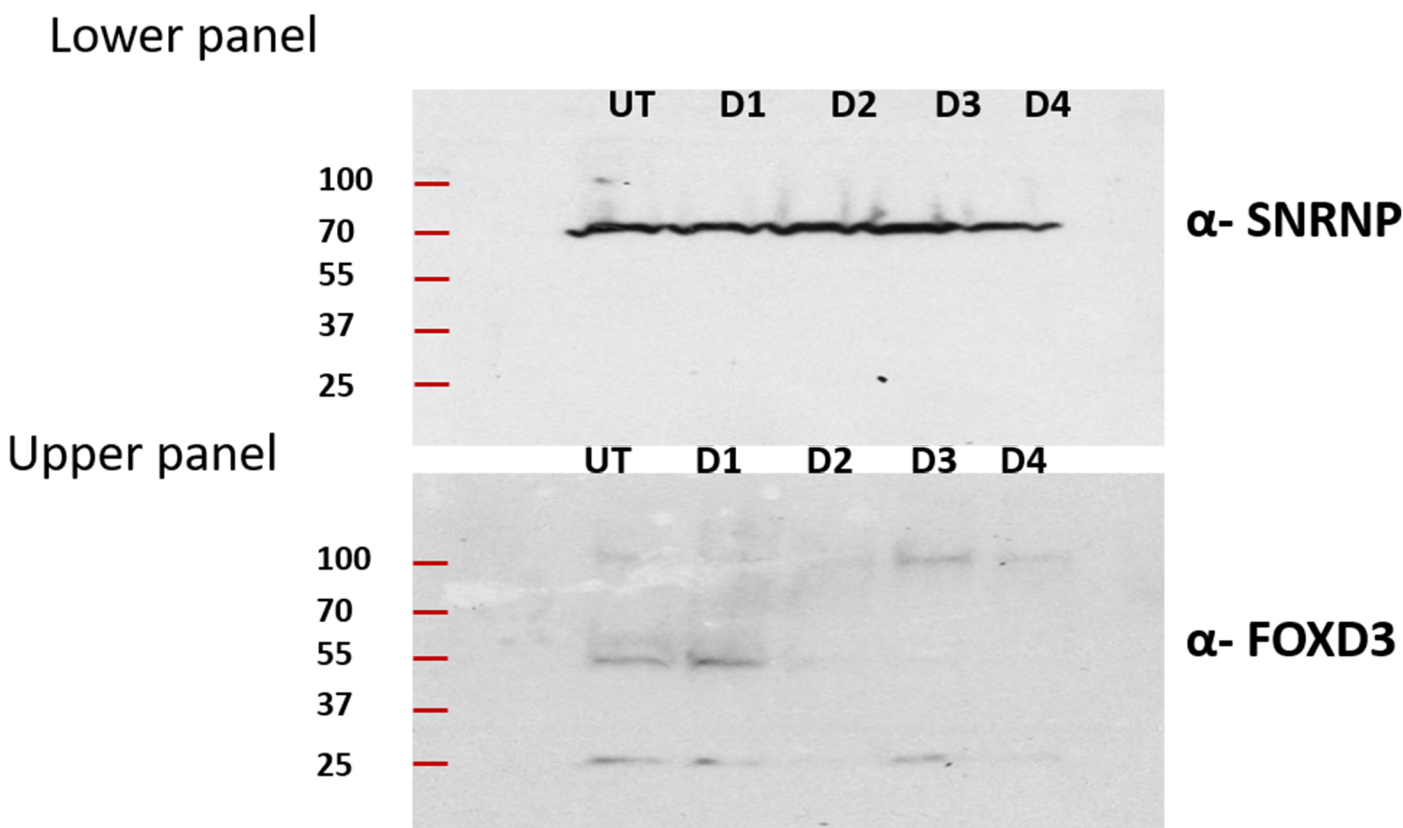

Supplement: Supplementary file 6 — Source Data for Expanded View [file EMBR-22-e53180-s004.zip › EV_Figure_Source_Data/Figure_Source_Data_EV2B-sd.pdf]

Figure 4a

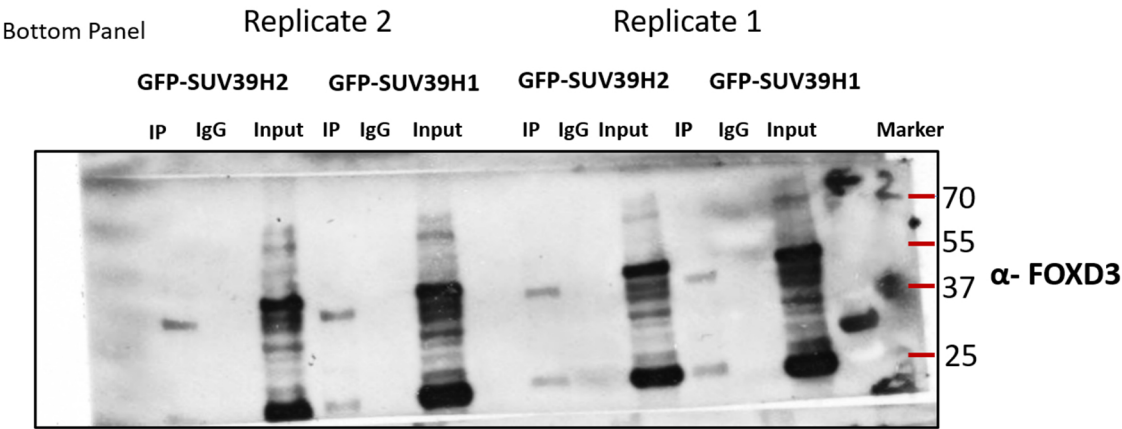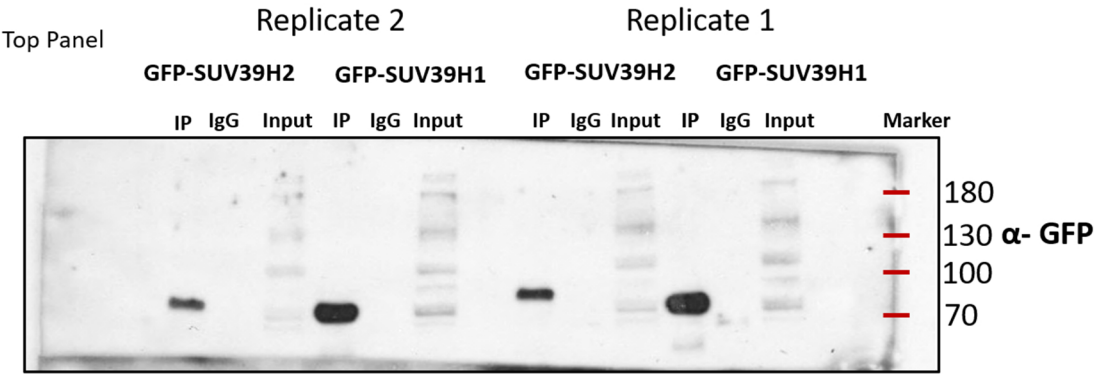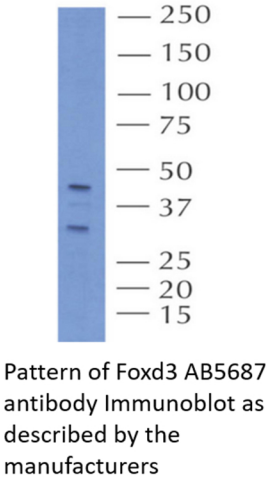

Supplement: Supplementary file 7 — Source Data for Figure 4 [file EMBR-22-e53180-s007.pdf]
