# Supplementary material for: Foxd3 controls heterochromatin‐mediated repression of repeat elements and 2‐cell state transcription
Source: EMBO Rep. 2021 Oct 4;22(12):e53180. doi: 10.15252/embr.202153180 (PMC8647145; doi:10.15252/embr.202153180)
Supplement: Supplementary file 8 [file EMBR-22-e53180-s001.pdf]

EMBO reports

Foxd3 controls heterochromatin-mediated repression of repeat elements and 2-cell state transcription

Deepika Puri, Birgit Koschorz, Bettina Engist, Megumi Onishi-seebacher, Devon Ryan, Mamilla Soujanya, and Thomas Montavon
DOI: 10.15252/embr.202153180

Corresponding author(s): Deepika Puri (deepikapuri@nccs.res.in) , Deepika Puri (deepikapuri@nccs.res.in)

Review Timeline:

Submission Date:	3rd May 21
Editorial Decision:	2nd Jun 21
Revision Received:	8th Jul 21
Editorial Decision:	4th Aug 21
Revision Received:	18th Aug 21
Accepted:	14th Sep 21

Editor: Achim Breiling

Transaction Report:

(Note: With the exception of the correction of typographical or spelling errors that could be a source of ambiguity, letters and reports are not edited. Depending on transfer agreements, referee reports obtained elsewhere may or may not be included in this compilation. Referee reports are anonymous unless the Referee chooses to sign their reports.)

1st Editorial Decision **2nd Jun 2021**

Dear Dr. Puri,

Thank you for the submission of your research manuscript to EMBO reports. We have now received the reports from the three referees that were asked to evaluate your study, which can be found at the end of this email.

As you will see, the referees think that these findings are of interest. However, they have several comments, concerns and suggestions, indicating that a major revision of the manuscript is necessary to allow publication of the study in EMBO reports. As the reports are below, and all their points need to be addressed, I will not detail them here.

Given the constructive referee comments, we would like to invite you to revise your manuscript with the understanding that all referee concerns must be addressed in the revised manuscript or in the detailed point-by-point response. Acceptance of your manuscript will depend on a positive outcome of a second round of review. It is EMBO reports policy to allow a single round of revision only and acceptance of the manuscript will therefore depend on the completeness of your responses included in the next, final version of the manuscript.

Revised manuscripts should be submitted within three months of a request for revision. We are aware that many laboratories cannot function at full efficiency during the current COVID-19/SARS-CoV-2 pandemic and we have therefore extended our 'scooping protection policy' to cover the period required for full revision. Please contact me to discuss the revision should you need additional time, and also if you see a paper with related content published elsewhere.

When submitting your revised manuscript, please also carefully review the instructions that follow below.

PLEASE NOTE THAT upon resubmission revised manuscripts are subjected to an initial quality control prior to exposition to re-review. Upon failure in the initial quality control, the manuscripts are sent back to the authors, which may lead to delays. Frequent reasons for such a failure are the lack of the data availability section (please see below) and the presence of statistics based on n=2 (the authors are then asked to present scatter plots or provide more data points).

When submitting your revised manuscript, we will require:

1) a .docx formatted version of the final manuscript text (including legends for main figures, EV figures and tables), but without the figures included. Please make sure that changes are highlighted to be clearly visible. Figure legends should be compiled at the end of the manuscript text.

2) individual production quality figure files as .eps, .tif, .jpg (one file per figure), of main figures and EV figures. Please upload these as separate, individual files upon re-submission.

The Expanded View format, which will be displayed in the main HTML of the paper in a collapsible format, has replaced the Supplementary information. You can submit up to 5 images as Expanded View. Please follow the nomenclature Figure EV1, Figure EV2 etc. The figure legend for these should be included in the main manuscript document file in a section called Expanded View Figure Legends after the main Figure Legends section. Additional Supplementary material should be supplied as a single pdf file labeled Appendix. The Appendix should have page numbers and needs

to include a table of content on the first page (with page numbers) and legends for all content. Please follow the nomenclature Appendix Figure Sx, Appendix Table Sx etc. throughout the text, and also label the figures and tables according to this nomenclature.

For more details, please refer to our guide to authors:
http://www.embopress.org/page/journal/14693178/authorguide#manuscriptpreparation

See also our guide for figure preparation:
http://wol-prod-cdn.literatumonline.com/pb-assets/embo-site/EMBOPress_Figure_Guidelines_061115-1561436025777.pdf

3) a .docx formatted letter INCLUDING the reviewers' reports and your detailed point-by-point responses to their comments. As part of the EMBO Press transparent editorial process, the point-by-point response is part of the Review Process File (RPF), which will be published alongside your paper.

4) a complete author checklist, which you can download from our author guidelines (https://www.embopress.org/page/journal/14693178/authorguide). Please insert page numbers in the checklist to indicate where the requested information can be found in the manuscript. The completed author checklist will also be part of the RPF.

Please also follow our guidelines for the use of living organisms, and the respective reporting guidelines: http://www.embopress.org/page/journal/14693178/authorguide#livingorganisms

5) that primary datasets produced in this study (e.g. RNA-seq, ChIP-seq, structural and array data) are deposited in an appropriate public database. If no primary datasets have been deposited, please also state this a dedicated section (e.g. 'No primary datasets have been generated and deposited'), see below.

See also: http://embor.embopress.org/authorguide#datadeposition

Please remember to provide a reviewer password if the datasets are not yet public.

The accession numbers and database should be listed in a formal "Data Availability " section (placed after Materials & Methods) that follows the model below. This is now mandatory (like the COI statement). Please note that the Data Availability Section is restricted to new primary data that are part of this study.

Data availability

The datasets produced in this study are available in the following databases:

- RNA-Seq data: Gene Expression Omnibus GSE46843 (https://www.ncbi.nlm.nih.gov/geo/query/acc.cgi?acc=GSE46843)
- [data type]: [name of the resource] [accession number/identifier/doi] ([URL or identifiers.org/DATABASE:ACCESSION])

*** Note - All links should resolve to a page where the data can be accessed. ***

Moreover, I have these editorial requests:

6) We strongly encourage the publication of original source data with the aim of making primary data more accessible and transparent to the reader. The source data will be published in a separate source data file online along with the accepted manuscript and will be linked to the relevant figure. If you would like to use this opportunity, please submit the source data (for example scans of entire gels or blots, data points of graphs in an excel sheet, additional images, etc.) of your key experiments together with the revised manuscript. If you want to provide source data, please include size markers for scans of entire gels, label the scans with figure and panel number, and send one PDF file per figure.

7) Our journal encourages inclusion of *data citations in the reference list* to directly cite datasets that were re-used and obtained from public databases. Data citations in the article text are distinct from normal bibliographical citations and should directly link to the database records from which the data can be accessed. In the main text, data citations are formatted as follows: "Data ref: Smith et al, 2001" or "Data ref: NCBI Sequence Read Archive PRJNA342805, 2017". In the Reference list, data citations must be labeled with "[DATASET]". A data reference must provide the database name, accession number/identifiers and a resolvable link to the landing page from which the data can be accessed at the end of the reference. Further instructions are available at:
http://www.embopress.org/page/journal/14693178/authorguide#referencesformat

8) Regarding data quantification and statistics, can you please specify, where applicable, the number "n" for how many independent experiments (biological replicates) were performed, the bars and error bars (e.g. SEM, SD) and the test used to calculate p-values in the respective figure legends. Please provide statistical testing where applicable, and also add a paragraph detailing this to the methods section. See:
http://www.embopress.org/page/journal/14693178/authorguide#statisticalanalysis

9) Please also note our new reference format:
http://www.embopress.org/page/journal/14693178/authorguide#referencesformat

10) For microscopic images, please add scale bars of similar style and thickness to all the microscopic images, using clearly visible black or white bars (depending on the background). Please place these in the lower right corner of the images. Please do not write on or near the bars in the image but define the size in the respective figure legend.

I look forward to seeing a revised version of your manuscript when it is ready. Please let me know if you have questions or comments regarding the revision.

Yours sincerely,

Achim Breiling
Editor
EMBO Reports

Referee #1:

Puri et al. study a potential role of Foxd3 in repressing the expression of repeat elements such as MERVL that are characteristic of the mouse 2-cell stage. They convincingly show that Foxd3 binds to MERVL and MSR using gel shift assays. Conditional Foxd3 knockout in mouse ES cells

reactivates repeat element expression and some genes associated with the 2 cell (2C) state. They identify that Foxd3 interacts with the histone methyltransferase Suv39h1 to regulate H3K9me3 levels at MERVL loci.

Identifying novel regulators of the 2C state would be of general interest. The main limitation of the study is that both the physiological relevance and the relative importance of Foxd3-mediated repression of MERVL expression compared to other mechanisms are not addressed. Another major concern is that Foxd3 is essential for mouse ES cell maintenance. Indeed the authors' Foxd3 cKO cells die four days after tamoxifen treatment and it questions how healthy and biologically relevant cells are after two days of treatment. The authors should also demonstrate that their proposed mechanism is Dux-independent (by using Dux KO ES cells).

Major concerns:

- To better characterize the contribution of Foxd3 to MERVL expression regulation, the authors should derive a double reporter line using the MERVL regulatory sequence (one wild type and one with a deletion of the Foxd3 binding site) driving fluorescent protein expression. Such reporters have been very valuable to study how sensitive the 2C state is to external perturbations. Assessing the fluorescence of the two reporters in individual cells will establish the relative contribution of Foxd3. The Delta-Foxd3 reporter should be in theory insensitive to acute perturbations of Foxd3 levels while the wild type one should respond.

- The role of Foxd3 in the spontaneous reactivation of the 2C state in mouse ES cells has to be characterized. How do Foxd3 protein levels in MERVL-positive cells compare to MERVL-negative cells? Does Foxd3 overexpression prevent the spontaneous appearance of the 2C state in ES cells?

- The authors overlooked that there is an upregulation of Dux (also known as AW822073 and listed as such in supplemental sheet 2) in their dataset upon Foxd3 cKO. This upregulation might be sufficient to induce the 2C state, Dux being such an important regulator of MERVL expression. The activation status of MERVL upon Foxd3 cKO should be repeated in a Dux KO background.

Minor concerns:

- In figure S3, GFP-Foxd3 has a different molecular weight compared to the two mutants. The three proteins have only amino acid replacements and should in theory migrate at the same size. This questions the integrity of the three constructs.

- There is only minimal overlap between genes bound by Foxd3 and the ones upregulated in Foxd3 KO cells (Figure S2b). This suggests that the bulk of the transcriptional changes in Foxd3 KO cells are not directly due to Foxd3 DNA binding activity.

Referee #2:

D. Puri and colleagues studied FOXD3 function in mouse embryonic stem cells. The authors found FOXD3 predicted binding sites at a few repeats including MERVL and major satellites. Foxd3 conditional knockout in embryonic stem cells promotes upregulation of MERVL retrotransposons, major satellites repeats and a subset of 2C-like cells marker genes. Mechanistically, the authors claimed that FOXD3 binds MERVL and major satellite repeats to trigger silencing by recruiting

SUV39H HMTases. The findings are not in line with previous Suv39h papers, and it is not discussed. Moreover, the experiments are not yet convincing enough.

Major issues:
1. concerning the ChIP of FOXD3:
The ChIP-qPCR is not convincing, since the authors use only one primer pair per repeat element type. TFs should not bind across the entire element but only to their binding site. For MERVL-Int, the sequence is several Kb long and there are two putative binding sites. The authors should do ChIP-seq so we can assess whether there is specific binding to the FoxD3 cognate motif, or at minimum show multiple qPCRs at primers that walk along each repeat class. The authors should also show a schematic of each repeat type, indicating the position of the primers used as well as the positions of the putative binding sites.
2. concerning the protein interaction between FOXD3 and SUV39H.
STRING analysis Fig 4a: could the authors explain how this figure was generated? For each connection what is the color legend? Does it show a demonstrated protein:protein interaction, and if so via what method? Those networks seem to show 'interaction' between SUV39H and SETDB1, that have never been shown to our knowledge.
IP Fig4b: very weak FOXD3 presence in SUV39H IP. Did the authors perform the reverse experiment, did they retrieve SUV39H in FOXD3 IP? Did they use mass spectrometry to be accurate?
3. concerning the recruitment of SUV39H to MERVL
ChIP Fig 4d: Bulut Karslioglu et al, published a careful HA tagged SUV39H1/2 ChIPseq. The authors must also analyze SUV39H binding at MERVL in this dataset.
RNAseq Fig 4c: it is inadequate to show a bigwig IGV snapshot showing two isolated genomic elements and claim that MERVL are upregulated in Suv39hdn ESC. The authors should carefully analyze the RNAseq dataset, with unique and multimapper strategeies and generate conventional figures such as volcano plots and heatmaps and show that only specific repeat types are affected. Alternatively a northern-blot or a RT qPCR in Suv39hdn cells and parental cells.
4. loss of SUV39H recruitment at MERVL and at pericentromeric regions.
The authors should perform an IF of FOXD3 that should be enriched at DAPI rich region corresponding to chromocenters.
In the ChIP Fig4d and 4e the authors show loss of SUV39H1 and H3K9me3 at pericentromeric regions. The authors should confirm the loss of H3K9me3 at pericentromeric regions in Foxd3 KO by alternative methods such as ChIP slot-blot...this is much more accurate for quantification at highly repetitive regions such as pericentromeres and telomeres.
5. Finally, this work is not discussed in the context of the published reports by the Jenuwein and Shinkai groups that propose an RNA-based model for SUV39H stable association with PCH in 2017. FOXD3 deletion promotes increase in MSR transcription associated with loss of SUV39H1 and H3K9me3. Unexpectedly, the authors did not comment.

Minor comments:
-percentage of immunofluorescence signal is very confusing
-Fig2c: MERVL gag staining should be cytoplasmic and not nuclear.
-There is a lot of overuse of the word repeats when the authors mean a specific repeat (like MERVL).
-The authors use "etc." instead of accurately describing what they mean.

Referee #3:

Puri et al. have identified FOXD3 as a repressor of MERVL elements in mouse ESCs (enacted at least in part by recruitment of SUV39H1/2), with downstream effects on the expression of 2-cell genes.

The key conclusions of the manuscript are well supported, and as far as can tell, novel. The work adds another seemingly important factor in the complex network that regulate the 2-cell state, although the in vivo relevance remains open for future studies. I have a few questions/suggestions for the consideration of the authors:

1. It seemed strange to focus solely on L1A elements as a representative L1 family, when the mouse genome harbours another two active families (L1Tf and L1Gf). This is particularly relevant because findings on the role of L1s in early development predominantly pertain L1Tf. Also note that L1Gf comes up in the supplementary data as regulated by FOXD3. Lastly, I would expect L1Tf to reveal YY1 binding motifs (and likely others), which makes an interesting contrast to L1A, and would place YY1 as another common factor amongst the different repeat families tested.

2. Seems like a subset of L1A elements (L1MdA_VI subfamily) are regulated by FOXD3, according to the supplementary data. It may be worth checking this specific consensus for FOXD3 binding motifs. I should be clear that it is not my intention to detract the attention of the paper from MERVL, which I agree should be its main focus.

3. It would add to the ChIP data if publicly available ChIP-seq data on FOXD3 was analysed, to gauge the binding profile along full-length elements, and to assess whether MERVL is particularly prominent in the amount of ChIP-seq signal when compared with other repeats.

4. The fact that mutant FOXD3 can still largely silence Zscan4 and Dppa3 suggests roles independent of FOXD3 DNA binding activity. This possibility is touched upon by the authors at one point, but it feels that this could be more directly tied to the results in Fig. 3d.

5. Given that FOXD3 may also recruit SETDB1, the authors could add a discussion point on the possibility that SETDB1 may add to the role of SUV39H1/2, especially as, judging from Fig. 4c, the effect of Suv39h1/2 KO is milder than that of Foxd3 KO.

6. The data on Fig. 4c does not look to be uniquely aligned, which gives a misleading view of of the data. Perhaps it is best to show an alignment to the consensus sequence.

7. I could not find Figures S2-4 in the files I downloaded. I did find them in the preprint version of the manuscript, so I am assuming that these match the submitted version.

8. Page 4 - "Whether other transcription factors contribute to repeat regulation remains unknown". This is inaccurate, given the extent of research in the TE regulation field. Also feels unnecessary, so my suggestion is to remove this sentence.

9. There are some inconsistencies in nomenclature, e.g., L1Mda (L1MdA), Mervl (MERVL).

Miguel Branco

Major changes in the revised manuscript:

- The manuscript has been restructured and the revised version now consists of 4 main figures and 5 extended version figures.
- The revised manuscript has an additional author (Mamilla Soujanya) who performed bioinformatics analysis that has been added to the manuscript
- All the reviewers' comments have been addressed in the revised manuscript as shown below

#1:

Puri et al. study a potential role of Foxd3 in repressing the expression of repeat elements such as MERVL that are characteristic of the mouse 2-cell stage. They convincingly show that Foxd3 binds to MERVL and MSR using gel shift assays. Conditional Foxd3 knockout in mouse ES cells reactivates repeat element expression and some genes associated with the 2 cell (2C) state. They identify that Foxd3 interacts with the histone methyltransferase Suv39h1 to regulate H3K9me3 levels at MERVL loci.

Identifying novel regulators of the 2C state would be of general interest. The main limitation of the study is that both the physiological relevance and the relative importance of Foxd3-mediated repression of MERVL expression compared to other mechanisms are not addressed.

We appreciate the reviewer's comment. We have addressed the physiological relevance of our findings in the revised manuscript (Page 14, line 18 -Page 15, line 9)

Another major concern is that Foxd3 is essential for mouse ES cell maintenance. Indeed, the authors' Foxd3 cKO cells die four days after tamoxifen treatment and it questions how healthy and biologically relevant cells are after two days of treatment.

We thank the reviewer for this comment. Foxd3 cKO cells die four days after tamoxifen treatment, but are healthy at the two-day time point at which they are harvested as shown in the bright field images below. We would also like to point out that previous publications determining the function of FOXD3 in mouse ES cells have used Foxd3 cKO cells after 48-72 hours post tamoxifen (Liu & Labosky, 2008; Krishnakumar *et al*, 2016) indicating that the data obtained from these cells is valid and biologically relevant.

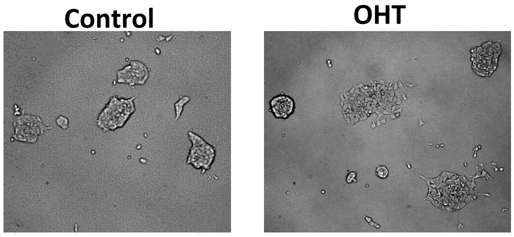

The authors should also demonstrate that their proposed mechanism is Dux-independent (by using Dux KO ES cells).

This point has been addressed below.

Major concerns

To better characterize the contribution of Foxd3 to MERVL expression regulation, the authors should derive a double reporter line using the MERVL regulatory sequence (one wild type and one with a deletion of the Foxd3 binding site) driving fluorescent protein expression. Such reporters have been very valuable to study how sensitive the 2C state is to external perturbations. Assessing the fluorescence of the two reporters in individual cells will establish the relative contribution of Foxd3. The Delta-Foxd3 reporter should be in theory insensitive to acute perturbations of Foxd3 levels while the wild type one should respond.

We thank the reviewer for this suggestion, however as India is facing an unprecedented wave of the Covid-19 pandemic, this has led to significant challenges to the functioning of labs. We are still in a lockdown and are not functioning at full capacity. Additionally, we face a severe shortage of resources and manpower. This, compounded with delays in funding as well as delays in shipping orders poses a grave challenge to the process of experiment planning and execution. Therefore, we are currently limited in resources and man power required for the establishment of the reporter cell lines. Even if the MERVL: td tomato reporter construct is bought or received as a gift, the unprecedented delays in shipping of materials that we face makes it difficult to predict the timeframe within which this can be achieved.

That said, it should be noted that we have clearly shown that FOXD3 binds to MERVL, mutant FOXD3 fails to bind MERVL in-vitro as well and in ES cells (Figure 1 and 3) and overexpression of full length FOXD3 and not mutant FOXD3 leads to rescue of MERVL expression and well as expression of 2C genes both at transcript and protein level, directly in Foxd3 KO ES cells (Figure 3). This indicates clearly that FOXD3 significantly contributes to MERVL regulation. We have reinforced this point by analysing FOXD3 ChIP-Seq data that identifies MERVL as a target for FOXD3 (Figure 1d). We also demonstrate by gel shift assay that FOXD3 binding to the MERVL and MSR sequences is abrogated when the binding site in the sequence is mutated (EV1b).

The role of Foxd3 in the spontaneous reactivation of the 2C state in mouse ES cells has to be characterized. How do Foxd3 protein levels in MERVL-positive cells compare to MERVL-negative cells? Does Foxd3 overexpression prevent the spontaneous appearance of the 2C state in ES cells?

We appreciate this question. Determining FOXD3 protein levels by isolating MERVL-positive and negative cells is beyond the scope of our ability in the current situation because of the difficulties in obtaining the MERVL-reporter construct. However, analysis of published RNA-Seq data from MERVL+ and MERVL- mESCs (Macfarlan *et al*, 2012) showed a modest reduction of Foxd3 transcription in MERVL positive cells compared to MERVL negative cells (Figure EV5 g). We have also addressed the effect of Foxd3 overexpression in ES cells in the revised manuscript (Figure EV4e) (Page 11, line 11-20; Page 14, line 18-22)

The authors overlooked that there is an upregulation of Dux (also known as AW822073 and listed as such in supplemental sheet 2) in their dataset upon Foxd3 cKO. This upregulation might be sufficient to induce the 2C state, Dux being such an important regulator of MERVL expression. The activation status of MERVL upon Foxd3 cKO should be repeated in a Dux KO background.

The murine *Dux* tandem repeat encodes two main transcripts, full-length *Dux* (or *Duxf3*) and a variant named *Gm4981* lacking the first homeodomain, along with an additional EST, AW822073. Our data only detects the upregulation of the EST and not the major transcripts indicating that the

effect of Foxd3 is not dependent on expression of Dux. We do not discount the role played by Dux in the conversion of ESC to 2CLCs and acknowledge the possibility of the small level of Dux expression may contribute to establishment of the 2C state. The scope of our manuscript however is limited to establishing a novel regulatory mechanism for repeat element transcription via direct binding and repression by Foxd3. We have discussed this point in the revised manuscript (Page 8 line 24-page 9 line 8). We have also added a figure to the supplementary data (Figure EV3e) confirming that the expression of the major Dux transcripts is unchanged in Foxd3 KO cells.

Minor concerns:

In figure S3, GFP-Foxd3 has a different molecular weight compared to the two mutants. The three proteins have only amino acid replacements and should in theory migrate at the same size. This questions the integrity of the three constructs.

We understand the reviewer's concern, however, we would like to point out that it is not uncommon that proteins migrate in gels at different speeds depending on numerous factors. The expected band size for the Foxd3+GFP protein is 70 KDa and this band is observed in all three samples. The additional higher molecular weight band in the GFP-FOXD3 overexpressing cells may be a result of post-translational modifications such as glycosylation or ubiquitylation which are known to add to the molecular weight of the protein. We have replaced figure EV4d with a more comprehensive immunoblot that includes two separate biological replicates used in our experiments with similar results, indicating that the upper band seen in the first data set is a result of technical variation.

There is only minimal overlap between genes bound by Foxd3 and the ones upregulated in Foxd3 KO cells (Figure S2b). This suggests that the bulk of the transcriptional changes in Foxd3 KO cells are not directly due to Foxd3 DNA binding activity.

We have discussed the DNA binding -independent role of FOXD3 in the revised manuscript (Page 8, line 1-8, Page 11 line 9-12)

Referee #2:

D. Puri and colleagues studied FOXD3 function in mouse embryonic stem cells. The authors found FOXD3 predicted binding sites at a few repeats including MERVL and major satellites. Foxd3 conditional knockout in embryonic stem cells promotes upregulation of MERVL retrotransposons, major satellites repeats and a subset of 2C-like cells marker genes. Mechanistically, the authors claimed that FOXD3 binds MERVL and major satellite repeats to trigger silencing by recruiting SUV39H HMTases. The findings are not in line with previous Suv39h papers, and it is not discussed.

We thank the reviewer for this observation, however, we would like to point out that transcription factor based regulation of repeat elements has now been established by reports from various labs (Eckersley-Maslin *et al*, 2019; Guallar *et al*, 2012), in particular PAX3 and PAX9 have been shown to bind to and repress major satellite repeats by regulating the establishment of the H3K9me3 mark in MEF cells (Bulut-Karslioglu *et al*, 2012). The role of SUV39H enzymes in regulating MSRs and ERVs has also been reported (Bulut-Karslioglu *et al*, 2014). In the revised manuscript we have conducted

detailed analysis of published Chip-Seq data sets to validate the enrichment of SUV39H1 and H3K9me3 over MERVL and MSRs as well as RNA Seq data that determine that MERVL transcription is upregulated in the absence of SUV39H1 and SUV39H2. This is in addition to the previously established role of SUV39H enzymes in regulating MSRs. These findings clearly establish the role of FOXD3 in the regulation of MERVL and MSR by recruiting heterochromatin HMTs.

Moreover, the experiments are not yet convincing enough.

Major issues:

1. concerning the ChIP of FOXD3:

The ChIP-qPCR is not convincing, since the authors use only one primer pair per repeat element type. TFs should not bind across the entire element but only to their binding site. For MERVL-Int, the sequence is several Kb long and there are two putative binding sites. The authors should do ChIP-Seq so we can assess whether there is specific binding to the FoxD3 cognate motif, or at minimum show multiple qPCRs at primers that walk along each repeat class. The authors should also show a schematic of each repeat type, indicating the position of the primers used as well as the positions of the putative binding sites.

We have analysed published FOXD3 Chip-Seq data and have added three figures (Figure 1c and EV1c and d) to the revised manuscript to validate the enrichment of FOXD3 on MERVL. We have also performed ChIP-qPCR using additional primers for MERVL-int and LINE-1 and added a schematic to represent each repeat type, including the FOXD3 binding site and primers used for qPCR analysis (Figure 1d and EV1e and f) to confirm specific binding of FOXD3 to target sites and not to other regions within the repeat element.

2. Concerning the protein interaction between FOXD3 and SUV39H.

STRING analysis Fig 4a: could the authors explain how this figure was generated? For each connection what is the color legend? Does it show a demonstrated protein:protein interaction, and if so via what method? Those networks seem to show 'interaction' between SUV39H and SETDB1, that have never been shown to our knowledge.

We have added a detailed legend to the figure depicting STRING analysis (Figure EV5a). It should be noted however, that the interaction between SUV39H and SETDB1 has been reported by previous studies (Fritsch *et al*, 2010) which indicate that a subset of H3K9me3 HMTs including SUV39H1, SETDB1, G9a and GLP interact with each other to form a H3K9 methylation multimeric complex.

IP Fig4b: very weak FOXD3 presence in SUV39H IP. Did the authors perform the reverse experiment, did they retrieve SUV39H in FOXD3 IP? Did they use mass spectrometry to be accurate?

We thank the reviewer for this valuable suggestion. We have now performed a reverse IP and are able to detect an enrichment of SUV39H1 in FOXD3 IP (Figure EV5b). We have also mentioned in our manuscript that the FOXD3-SUV39H1 interaction is sub stochiometric (Pag2 12, line 13-15)

3. concerning the recruitment of SUV39H to MERVL

ChIP Fig 4d: Bulut Karslioglu et al, published a careful HA tagged SUV39H1/2 ChIPseq. The authors must also analyze SUV39H binding at MERVL in this dataset.

We have analysed the HA SUV39H1/H2 ChIP-Seq data published in (Bulut-Karslioglu *et al*, 2014) and have observed SUV39H enrichment over MERVL. We have included this analysis in Figure 4c of the revised manuscript. We have also reinforced our findings by performing the analysis of H3K9me3 ChIP-Seq data in WT and *Suv39h dn* ES cells (Bulut-Karslioglu *et al*, 2014) and show an reduction in this repressive mark over MERVL in the absence of SUV39H enzymes (Figure 4f) validating the regulation of MERVL by SUV39H HMTs.

RNAseq Fig 4c: it is inadequate to show a bigwig IGV snapshot showing two isolated genomic elements and claim that MERVL are upregulated in Suv39hdn ESC. The authors should carefully analyze the RNAseq dataset, with unique and multimapper strategeies and generate conventional figures such as volcano plots and heatmaps and show that only specific repeat types are affected.

We have carefully analysed the RNA Seq data set and have added a figure (Figure 4b) to the revised manuscript depicting the upregulation of MERVL in Suv39h dn mESCs.

4. Loss of SUV39H recruitment at MERVL and at pericentromeric regions.
The authors should perform an IF of FOXD3 that should be enriched at DAPI rich region corresponding to chromocenters.

We have confirmed the localisation of FOXD3 in mESCs by performing IF in control ES cells and FOXD3si ES cells. We do not see a pericentric localisation of FOXD3, rather a dispersed nuclear localisation (Figure 4g). This is consistent with reports that determine FOXD3 binding to genes as well as numerous intergenic enhancer elements (Krishnakumar *et al*, 2016; Respuela *et al*, 2016)

In the ChIP Fig4d and 4e the authors show loss of SUV39H1 and H3K9me3 at pericentromeric regions. The authors should confirm the loss of H3K9me3 at pericentromeric regions in Foxd3 KO by alternative methods such as ChIP slot-blot...this is much more accurate for quantification at highly repetitive regions such as pericentromeres and telomeres.

We have performed siRNA-based knockdown of Foxd3 in *Suv39h dn* mESCs expressing GFP-SUV39H1 followed by immunofluorescence for FOXD3, H3K9me3 and GFP detection representing SUV39H1. We clearly see a loss of both H3K9me3 and SUV39H1 from the pericentric regions in cells Foxd3i cells. The schematic and the results have been depicted in Figure 4g and EV5e in the revised manuscript.

Finally, this work is not discussed in the context of the published reports by the Jenuwein and Shinkai groups that propose an RNA-based model for SUV39H stable association with PCH in 2017. FOXD3 deletion promotes increase in MSR transcription associated with loss of SUV39H1 and H3K9me3. Unexpectedly, the authors did not comment.

We appreciate this observation. We have discussed our findings in the context of the MSR RNA mediated association of Suv39h to chromatin (Page 14, line 3-13) in the revised manuscript.

Minor comments:
-percentage of immunofluorescence signal is very confusing

The depiction of Percentage of IF signal has been changed to improve clarity
-Fig2c: MERVL gag staining should be cytoplasmic and not nuclear.

We appreciate this observation. Indeed, the MERVL-gag staining in our experiments is cytoplasmic as seen clearly in figure 3c. It should be noted that the final images in the figures are depicted as maximum intensity projections of z stacks and owing to the high nucleus: cytoplasm ratio of mESCs clear cytoplasmic staining may difficult to perceive. For clarity, we have included below, a different image for MERVL staining in Foxd3 KO cells that shows clear exclusion of nuclear localisation.

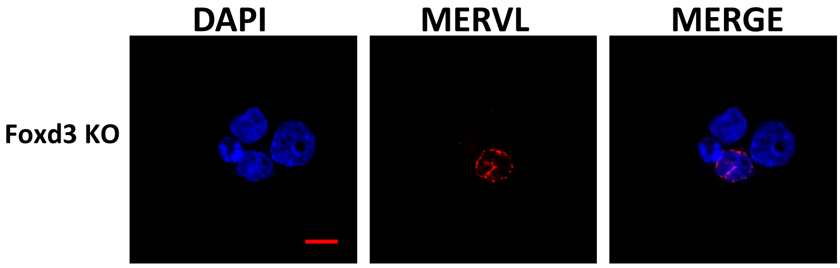

-

There is a lot of overuse of the word repeats when the authors mean a specific repeat (like MERVL).
-The authors use "etc." instead of accurately describing what they mean.

We have amended the revised manuscript to reduce the use of ambiguous phrases (Page 3 line 7, line 16, line 22, Page 4 line 12, Page 5 line 2, 19)

Referee #3:

Puri et al. have identified FOXD3 as a repressor of MERVL elements in mouse ESCs (enacted at least in part by recruitment of SUV39H1/2), with downstream effects on the expression of 2-cell genes.

The key conclusions of the manuscript are well supported, and as far as can tell, novel. The work adds another seemingly important factor in the complex network that regulate the 2-cell state, although the in vivo relevance remains open for future studies. I have a few questions/suggestions for the consideration of the authors:

1. It seemed strange to focus solely on L1A elements as a representative L1 family, when the mouse genome harbours another two active families (L1Tf and L1Gf). This is particularly relevant because findings on the role of L1s in early development predominantly pertain L1Tf. Also note that L1Gf comes up in the supplementary data as regulated by FOXD3. Lastly, I would expect L1Tf to reveal YY1 binding motifs (and likely others), which makes an interesting contrast to L1A, and would place YY1 as another common factor amongst the different repeat families tested.

We thank the reviewer for this comment. We have included L1Tf, L1Gf as well as L1F in our analysis and as mentioned by the reviewer YY1 comes up as a common TF for all the repeat elements analysed except L1MdA. We have included these findings in Figure 1a and Figure EV1a.

2. Seems like a subset of L1A elements (L1MdA_VI subfamily) are regulated by FOXD3, according to the supplementary data. It may be worth checking this specific consensus for FOXD3 binding motifs.

I should be clear that it is not my intention to detract the attention of the paper from MERVL, which I agree should be its main focus.

We have addressed the modest upregulation of a subset of LINE elements in Foxd3 KO cells (Page 9 line 13-15)

3. It would add to the ChIP data if publicly available ChIP-Seq data on FOXD3 was analysed, to gauge the binding profile along full-length elements, and to assess whether MERVL is particularly prominent in the amount of ChIP-Seq signal when compared with other repeats.

We have analysed published FOXD3 Chip-Seq data and have added three figures (Figure 1c and EV1c and d) to the revised manuscript to validate the enrichment of Foxd3 on MERVL. We have also performed ChIP-qPCR using additional primers for MERVL-int and LINE-1 and added a schematic to represent each repeat type, including the FOXD3 binding site and primers used for qPCR analysis (Figure 1d and EV1e and f) to confirm specific binding of FOXD3 to target sites and not to other regions within the repeat element.

4. The fact that mutant FOXD3 can still largely silence Zscan4 and Dppa3 suggests roles independent of FOXD3 DNA binding activity. This possibility is touched upon by the authors at one point, but it feels that this could be more directly tied to the results in Fig. 3d.

We have discussed the potential binding-independent regulation of FOXD3 in the revised manuscript (Page 8, Line 1-8; Page 11, Line 9-12) and have discussed this in the context of the results shown in Figure 3d

5. Given that FOXD3 may also recruit SETDB1, the authors could add a discussion point on the possibility that SETDB1 may add to the role of SUV39H1/2, especially as, judging from Fig. 4c, the effect of Suv39h1/2 KO is milder than that of Foxd3 KO.

We have discussed the role of SETDB1 in regulating MERVL (Page 13 line 19-24) in the revised manuscript. We were unable to detect an interaction between FOXD3 and SETDB1 in our immunoprecipitation experiments (Figure EV5b) and posit that the role of SETDB1 in MERVL regulation is not dependent on FOXD3.

6. The data on Fig. 4c does not look to be uniquely aligned, which gives a misleading view of of the data. Perhaps it is best to show an alignment to the consensus sequence.

We have carefully analysed the RNA Seq data set and have added a figure (Figure 4b) to the revised manuscript depicting the upregulation of MERVL in *Suv39h dn* mESCs.

7. I could not find Figures S2-4 in the files I downloaded. I did find them in the preprint version of the manuscript, so I am assuming that these match the submitted version.

We apologise for the inconvenience and confirm that the files were uploaded at the time of submission.

8. Page 4 - "Whether other transcription factors contribute to repeat regulation remains unknown". This is inaccurate, given the extent of research in the TE regulation field. Also feels unnecessary, so my suggestion is to remove this sentence.

We have removed this sentence from the revised manuscript.

9. There are some inconsistencies in nomenclature, e.g., L1Mda (L1MdA), Mervl (MERVL).

We have carefully proofread the revised manuscript and have amended the inconsistencies in nomenclature.

1st Revision - Editorial Decision 4th Aug 2021

Dear Dr. Puri,

Thank you for the submission of your revised manuscript to our editorial offices. I have now received the reports from the three referees that were asked to re-evaluate your study, you will find below. As you will see, the referees now support the publication of your study in EMBO reports. Nevertheless referees #2 and #3 have remaining concerns and/or suggestions to improve the manuscript that I ask you to address in a final revised version of the manuscript. Please also provide a detailed point-by-point-response addressing the remaining concerns of the referee.

Moreover, I have these editorial requests:

- Please provide a shorter and more comprehensive title with not more than 100 characters (including spaces).

- Please remove the referee token from the Data Availability Section of the final revised manuscript and add a link to directly access the deposited dataset. Please make sure the dataset is public latest upon publication of the paper.

- Please make sure that all the funding information is entered into the online submission system and is complete and similar to the one in the manuscript text file.

- Please name the 'declaration of interests' 'Conflict of-interest statement' order the manuscript sections like this (using these section names):
Title page - Abstract - Introduction - Results - Discussion - Materials and Methods -Data availability section - Acknowledgements - Author contributions - Conflict of interest statement - References - Figure legends - Expanded View Figure legends - Expanded view Table legends.

- Please upload the datasets 'Sheet 1' and 'Sheet 2' named 'Dataset EV1' and 'Dataset EV2' with a legend on the first TAB of the excel sheet. Then please update the callouts in the manuscript text

- Please upload the two EV tables individually naming them 'Table EV1' and 'Table EV2', also in the file itself. Please also add legends for these to the main manuscript (after the EV figure legends) and check that the tables are called out correctly in the text (using 'Table EV1' and 'Table EV2').

- For the microscopic images, please add scale bars of similar style and thickness to all the microscopic images, using clearly visible black or white bars (depending on the background). Presently the scale bars in red are not very well visible. Please place these in the lower right or left corner of the images. Please do not write on or near the bars in the image but define the size in the respective figure legend.

- As they are significantly cropped, could you provide the source data for the few Western blots shown in the manuscript (including the EV figures)? The source data will be published in separate source data files online along with the accepted manuscript and will be linked to the relevant figures. Please submit scans of entire gels or blots together with the revised manuscript. Please include size markers for scans of entire gels, label the scans with figure and panel number, and send one PDF file per figure.

- Please make sure that the number "n" for how many independent experiments were performed,

their nature (biological versus technical replicates), the bars and error bars (e.g. SEM, SD) and the test used to calculate p-values is indicated in the respective figure legends (also of the EV figures), and that statistical testing has been done where applicable. Please avoid the phrase 'independent experiment', but clearly state if these were biological or technical replicates. Most diagrams have only partially statistics. If statistical testing was done but there is no significant difference, please also mark this in the diagrams (n.s.). Could statistical testing be done for Figs. EV1F, EV2A, EV3D, EV4E and EV5E, F and G?

- For the diagrams in Figs. 3B, 3D and EV2D you indicate that here data from 2 biological replicates is shown. If indeed n is 2, then statistical testing does not make much sense (with two replicates). In that case, please show the two datasets separately, without statistics and error bars. This is much more transparent and illustrates better the data. Or add a third replicate to do proper statistics.

- Finally, please find attached a word file of the manuscript text (provided by our publisher) with changes we ask you to include in your final manuscript text, and some queries, we ask you to address. It seems you already addressed these, but please re-check. Please provide your final manuscript file with track changes, in order that we can see any modifications done.

In addition, I would need from you:
- a short, two-sentence summary of the manuscript (not more than 35 words).
- two to four bullet points highlighting the key findings of your study.
- a schematic summary figure (in jpeg or tiff format with the exact width of 550 pixels and a height of not more than 400 pixels) that can be used as a visual synopsis on our website.

I look forward to seeing the final revised version of your manuscript when it is ready. Please let me know if you have questions regarding the revision.

Best,

Achim Breiling
Editor
EMBO Reports

Referee #1:

The authors addressed most of my comments in a satisfactory manner in the revised version of their manuscript. The derivation of new MERVL reporter ES cell lines was not performed. However, the additional data and analyses presented by the authors convincingly support their claims now.

Referee #2:

With additional experiments, the authors have made a more convincing case that FOXD3 loss induces
MSR and MERVL transcription in ESC. The authors found convincing loss of SUV39H both at MSR and MERVL associated with a H3K9me3 to H3K27me3 heterochromatin switch in Foxd3 negative cells. This recapitulates the Suv39hdn ESC phenotype at PCH-MERVL and is consistent with several previous reports from different labs. In contrast to Suv39hdn cells, the authors found that

loss of FOXD3 also promotes the activation of genes including 2CLC genes consistent with FOXD3 function as TF. They propose the original model that the TF FOXD3 binds and recruits SUV39H HMTases for H3K9me3 deposition and silencing specifically at MS and MERVL repeats. FoxD3 rescue experiments are also very nicely done.
Despite this, I still have a major concern that requires attention
The Bioinformatic analysis at repeat sequences is inadequate (for both ChIP-seq and RNA-seq) The authors do not describe how they map their reads to repeat sequences (for example how do they handle unmapped reads?) There are many predesigned tools available to do this which is essential for young repeats like MERVL and MSR which often cannot be mapped to uniquely. It is justified to map with random assignment of reads as well as to map to consensus elements. The heatmaps shown at repeat elements are not consistent with typical TF binding patterns, where peaks should be sharp and centered over motifs (for example look at the studies from Trono, Macfarlan, and Hughes labs that map KZFP binding to repeat sequences) and I suspect this is an issue with mapping. Do the authors have an explanation for why FOXD3 appears to coat the entire MERVL-LTR and -Int sequences, but only at a few elements, with the vast majority of elements being unbound? Do these unbound elements lack Foxd3 motifs or harbor a distinct chromatin state? Do these FOXD3 unbound elements also devoid from SUV39H binding?
Finally, the STRING analysis is not clear. First, the authors do not describe how they generate this figure in STRING, neither the parameters used for the analysis (confidence stringency?). Citing a method paper is not sufficient. Second, experimental validation what does it mean in term of cell type, organisms, homologues?

Referee #3:

The authors have largely addressed my queries. However, I have a technical comment on the newly added ChIP-seq analyses, which do add value to the manuscript. Judging from the methods description and the figures, the ChIP-seq data includes non-unique reads. This is fine for "pile-up" plots such as the ones in Figure 4f, but it is misleading to include them in cases where individuals copies are represented (heatmaps such as the one in Fig. 1c, and browser snapshots such as the one in Fig. EV1d). In these cases, only unique reads should be used or, if there is insufficient mappability, simply stick to the pile-up versions.

Miguel Branco

Referee #1:

The authors addressed most of my comments in a satisfactory manner in the revised version of their manuscript. The derivation of new MERVL reporter ES cell lines was not performed. However, the additional data and analyses presented by the authors convincingly support their claims now.

Referee #2:

With additional experiments, the authors have made a more convincing case that FOXD3 loss induces MSR and MERVL transcription in ESC. The authors found convincing loss of SUV39H both at MSR and MERVL associated with a H3K9me3 to H3K27me3 heterochromatin switch in Foxd3 negative cells. This recapitulates the Suv39hdn ESC phenotype at PCH-MERVL and is consistent with several previous reports from different labs. In contrast to Suv39hdn cells, the authors found that loss of FOXD3 also promotes the activation of genes including 2CLC genes consistent with FOXD3 function as TF. They propose the original model that the TF FOXD3 binds and recruits SUV39H HMTases for H3K9me3 deposition and silencing specifically at MS and MERVL repeats. FoxD3 rescue experiments are also very nicely done.
Despite this, I still have a major concern that requires attention
The Bioinformatic analysis at repeat sequences is inadequate (for both ChIP-seq and RNA-seq) The authors do not describe how they map their reads to repeat sequences (for example how do they handle unmapped reads?) There are many predesigned tools available to do this which is essential for young repeats like MERVL and MSR which often cannot be mapped to uniquely. It is justified to map with random assignment of reads as well as to map to consensus elements.

We have elaborated on the bio-informatic analysis in the materials and methods section to clarify mapping and peak calling parameters

The heatmaps shown at repeat elements are not consistent with typical TF binding patterns, where peaks should be sharp and centered over motifs (for example look at the studies from Trono, Macfarlan, and Hughes labs that map KZFP binding to repeat sequences) and I suspect this is an issue with mapping. Do the authors have an explanation for why FOXD3 appears to coat the entire MERVL-LTR and -Int sequences, but only at a few elements, with the vast majority of elements being unbound? Do these unbound elements lack Foxd3 motifs or harbor a distinct chromatin state? Do these FOXD3 unbound elements also devoid from SUV39H binding?

We appreciate this comment. Upon further literature review, data analysis and based on the suggestion of the reviewers, we conclude that depicting this data as a heat map does not portray accurately, FOXD3 binding to individual repeat elements; as non-unique reads are randomly assigned and plotting only unique reads reduces the mapability. We cannot conclusively determine whether the elements that appear unbound in the heat map are due to lack of enrichment or because they are not mapped. Additionally, in the case of pericentric MSRs, many of the repetitive regions are unmapped in the reference genome. Hence, addressing the presence of Foxd3 binding sites, SUV39H enrichment and chromatin state in the unbound elements would require prior conclusive quantification of whether a majority of repeats are indeed unbound and this cannot be done with the available dataset. Thus we have removed the heatmaps in our revised manuscript and have depicted "pile-up" meta data plots to summarize the FOXD3

enrichment over repeat elements to avoid misrepresentation (Figure 1C, 4C, 4F). We do see sharp peaks around sub-repeat 3 in the MSR plot and around the LTRs and the putative FOXD3 binding sites in the MERVL-int which dip further along the MERVL-int, (though not as clearly as is shown in the data published by Trono and Macfarlan labs e.g., (Wolf *et al*, 2020)). The lack of sharp peaks around the motif can be attributed to our mapping and plotting protocol. We plot all the annotated MERVL repeats (full-length as well as truncated) instead of only intact repeats as plotted in (Wolf *et al*, 2020). This might affect the alignment of the peaks as the data is scaled to fit repeat start and end . We have also centred our plots to repeat start and end and not to peaks as is done in (Wolf *et al*, 2020). This is to better depict the profile along the repeat. We have added profile plots where the data is centred around the FOXD3 peaks for MSR, MERVL and MT2_Mm (Figure EV1C) and do observe a sharp peak for MERVL and MT2_Mm. A broader peak for MSR can be attributed to the tandem repeat property of this element. Additionally, while we have predicted FOXD3 binding sites based on stringent cut-offs in our TF prediction algorithm, we cannot exclude the possibility of other FOXD3 binding sites along the repeat sequence which were not predicted by our algorithm due to our cut-offs, which would still be enriched for FOXD3. Analysis of individual MERVL loci (Figure EV1D) indicates that FOXD3 is largely enriched over defined regions of MERVL which presumably correspond to the FOXD3 binding site.

Finally, the STRING analysis is not clear. First, the authors do not describe how they generate this figure in STRING, neither the parameters used for the analysis (confidence stringency?). Citing a method paper is not sufficient

We have added details of the STRING analysis in the methods section and mentioned the steps taken and the parameters for analysis in the revised manuscript.

Second, experimental validation what does it mean in term of cell type, organisms, homologues?

We have attached the basis for identification of putative interaction between FOXD3 and SUV39H1 and SETDB1 below along with the

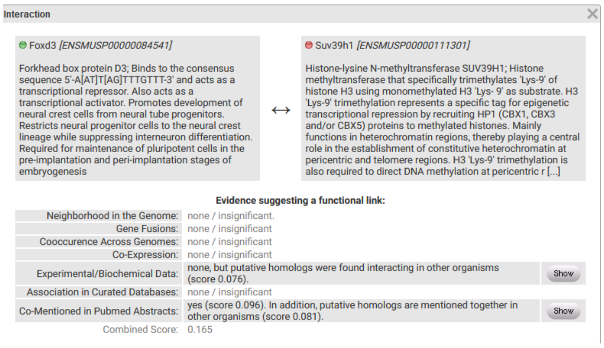

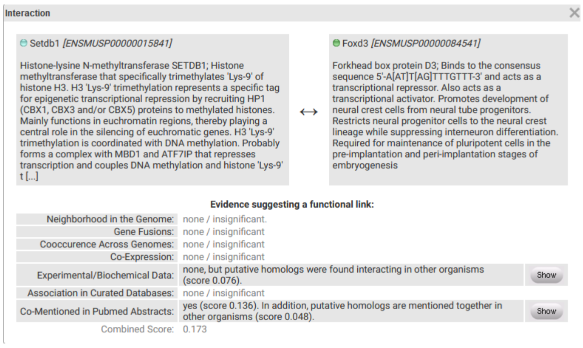

interaction score as calculated in STRING.

Referee #3:

The authors have largely addressed my queries. However, I have a technical comment on the newly added ChIP-seq analyses, which do add value to the manuscript. Judging from the methods description and the figures, the ChIP-seq data includes non-unique reads. This is fine for "pile-up" plots such as the ones in Figure 4f, but it is misleading to include them in cases where individual copies are represented (heatmaps such as the one in Fig. 1c, and browser snapshots such as the one

in Fig. EV1d). In these cases, only unique reads should be used or, if there is insufficient mappability, simply stick to the pile-up versions.

We thank the reviewer for this valuable observation. Upon further analysis, we agree with the reviewer that using heatmaps to represent these data would not accurately portray the enrichment at individual loci, as non-unique reads have been randomly assigned. Using only unique reads will not be feasible to map repeat elements due to reduced mapability. We have therefore, removed the heatmaps in figures 1C, 4C and EV1C and have displayed the data as 'Pile-up' metadata profiles.

Reference

Wolf G, de Iaco A, Sun M-A, Bruno M, Tinkham M, Hoang D, Mitra A, Ralls S, Trono D & Macfarlan TS (2020) KRAB-zinc finger protein gene expansion in response to active retrotransposons in the murine lineage. *eLife* 9: e56337

Dear Dr. Puri,

Thank you for the submission of your newly revised manuscript. I have taken over its handling as my colleague Achim is currently not in the office.

We have now received these comments from referee 2 on your revised study:

"Despite, a number of improvements (most notably the removal of heatmaps), the manuscript is still lacking rigor and clarity. Particularly, the ChIP-seq analysis is not clear, and the data presented is simply not consistent with sequence specific transcription factor binding for Foxd3. The authors have still not described accurately with enough clarity how reads were mapped for the metaplots presented in Figure 1 and how they were normalized. The authors now state in the methods that multi-mapped reads were randomly assigned but then do not describe how the metaplots were generated and what normalization (if any was performed). This is very important if the authors want to show flanking regions in these metaplots. Is the signal summarized across all family members and then divided by the number of family members in the genome (and if so this is very problematic for MSR, whose copy number could be quite variable and is therefore difficult to estimate). Furthermore the metaplot data presented (even if only considering the parts that map to the repeats and not the flank, which would frankly be more valuable), is still not consistent with sequence-specific transcription factor mediated binding, especially to MERVL repeats. The enrichment appears to be over the entire elements which casts serious doubts on the finding. (The paper would actually be improved by removing this dubious data at this stage because it does not support their conclusion that Foxd3 binds specifically to certain motifs within the MERVL family of elements.)"

These comments are important, and we cannot proceed with the handling of your manuscript until these issues are resolved to the satisfaction of experts in the field. We do not favour the removal of data at this point, but think that the data should rather be discussed in a transparent manner.

Referee 2 points out 1. an insufficient description of how the data analysis was performed and 2. that the current dataset does not support sequence specific binding of Foxd3 to the repeats.

Please let us know how you plan to address these outstanding issues.

Kind regards,
Esther

Esther Schnapp, PhD
Senior Editor
EMBO reports

Thank you for your email,

Reviewer 2's query has already been addressed in the last round of revisions in the point by point response. I am attaching the previous comments by reviewer 2 and our response to the same.

Comment: The heatmaps shown at repeat elements are not consistent with typical TF binding patterns, where peaks should be sharp and centered over motifs (for example look at the studies from Trono, Macfarlan, and Hughes labs that map KZFP binding to repeat sequences) and I suspect this is an issue with mapping. Do the authors have an explanation for why FOXD3 appears to coat the entire MERVL-LTR and -Int sequences, but only at a few elements, with the vast majority of elements being unbound? Do these unbound elements lack Foxd3 motifs or harbour a distinct chromatin state? Do these FOXD3 unbound elements also devoid from SUV39H binding?

Response: We appreciate this comment. Upon further literature review, data analysis and based on the suggestion of the reviewers, we conclude that depicting this data as a heat map does not portray accurately, FOXD3 binding to individual repeat elements; as non - unique reads are randomly assigned and plotting only unique reads reduces the mapability. We cannot conclusively determine whether the elements that appear unbound in the heat map are due to lack of enrichment or because they are not mapped. Additionally, in the case of pericentric MSRs, many of the repetitive regions are unmapped in the reference genome. Hence, addressing the presence of Foxd3 binding sites, SUV39H enrichment and chromatin state in the unbound elements would require prior conclusive quantification of whether a majority of repeats are indeed unbound and this cannot be done with the available dataset. Thus we have removed the heatmaps in our revised manuscript and have depicted "pile-up" meta data plots to summarize the FOXD3 enrichment over repeat elements to avoid misrepresentation (Figure 1C, 4C, EV1C). We do see sharp peaks around sub-repeat 3 in the MSR plot and around the LTRs and the putative FOXD3 binding sites in the MERVL-int which dip further along the MERVL-int, (though not as clearly as is shown in the data published by Trono and Macfarlan labs e.g., (Wolf *et al*, 2020)**). The lack of sharp peaks around the motif can be attributed to our mapping and plotting protocol. We plot all the annotated MERVL repeats (full-length as well as truncated) instead of only intact repeats as plotted in** (Wolf *et al*, 2020). **This might affect the alignment of the peaks as the data is scaled to fit repeat start and end . We have also centred our plots to repeat start and end and not to peaks as is done in** (Wolf *et al*, 2020). **This is to better depict the profile along the repeat. Additionally, while we have predicted FOXD3 binding sites based on stringent cut-offs in our TF prediction algorithm, we cannot exclude the possibility of other FOXD3 binding sites along the repeat sequence which were not predicted by our algorithm due to our cut-offs, which would still be enriched for FOXD3. Analysis of individual MERVL loci (Figure EV1D) indicates that FOXD3 is largely enriched over defined regions of MERVL which presumably correspond to the FOXD3 binding site.**

Hence it is not clear whether reviewer 2 has had a chance to look at our new figure (EV1C) which shows a classical TF binding peak and our point by point response that explains the reason behind the appearance of the peaks when the data is scaled to repat start and end as we have done.

Regarding the 1st point of concern, I have asked my bio-informatician to explain our methods in further detail to improve clarity. I shall make those changes in the main manuscript, although we have used ready-made published R packages to analyse and plot our data and I will be essentially repeating the protocol as published elsewhere.
However, regarding point 2, it is not clear why the same point is being raised. I would like to clarify again that TF binding profiles on single-copy genes may look different than that of repeats.

- **The lack of sharp peaks around the motif can be attributed to our mapping and plotting protocol. We plot all the annotated MERVL repeats (full-length as well as truncated) instead of only intact repeats as plotted in** (Wolf *et al*, 2020)**. This might affect the alignment of the peaks as the data is scaled to fit repeat start and end, irrespective of the repeat's size. It should be noted that we have centred our plots to repeat start and end and not to peaks as is done in** (Wolf *et al*, 2020)**.**
- **We have added figure EV1C , as suggested by reviewer 2 last time, in line with publications mentioned by the reviewer where the data is centred around Foxd3 peaks in repeat elements, and we do see a sharp peak for MERVL and MT2a. The broader peak for MSR may be a result of the tandem repeat nature of the element.**
- **Reports from other labs (including those mentioned by reviewer 2) depicting TF binding over repeat elements also exhibit enrichment not just over the motif, but as a broader peak.**
  Given below are representative examples from published data sets compared with our data.

  1. (Shi *et al*, 2019)

Published data **Our data**

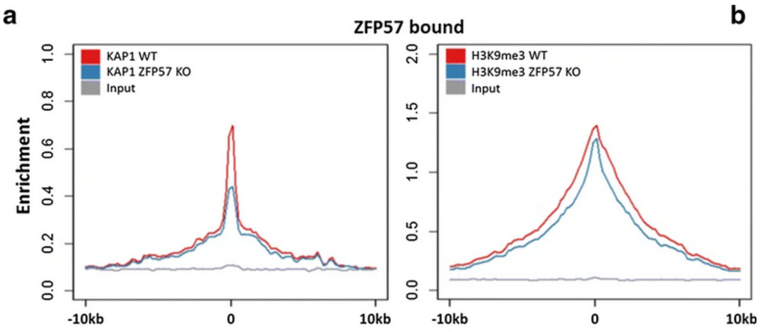

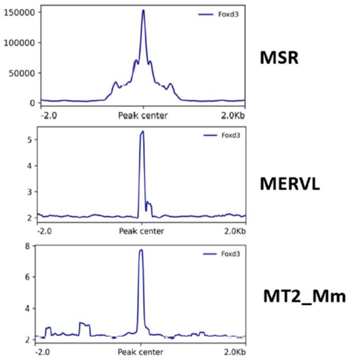

In this published figure, the data is centred around KAP1 peaks and KAP1 enrichment over transposable elements is plotted. Similar to our data (Figure EV1C), the Foxd3 peaks are sharp at the centre and taper in the flanks

Published data

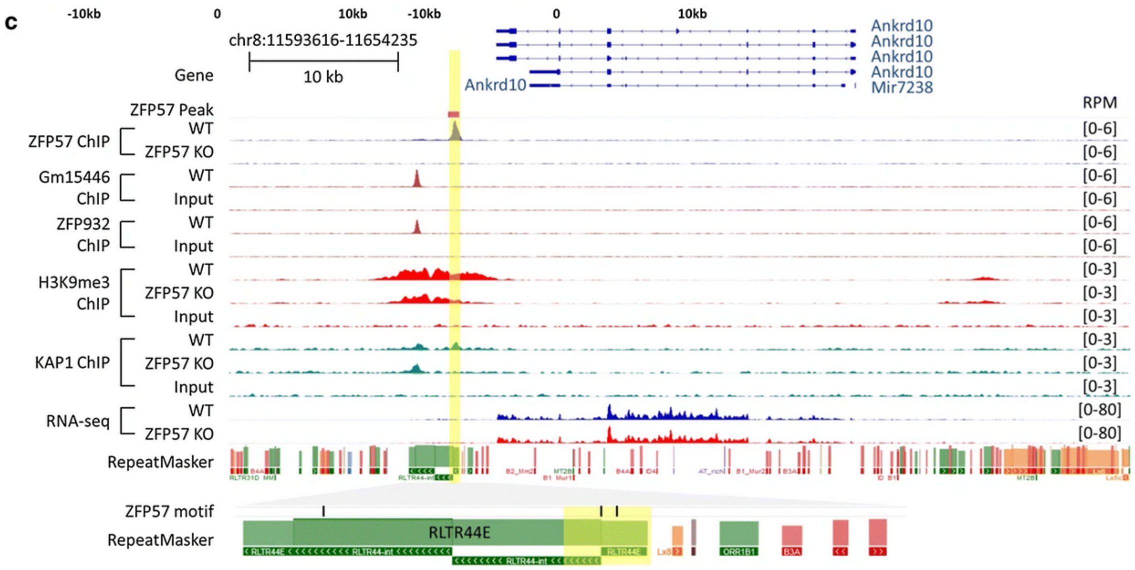

ZFP57 plays a largely redundant role in transposon silencing. **a** Reduction of KAP1 ChIP signals in ZFP57 KO under TE-bound ZFP57 peaks, which leads to very modest reduction in H3K9me3 levels (top), no such reduction was observed at other KAP-bound TEs in absence of ZFP57 binding (bottom). **b** Venn diagram showing partial overlapping ZFP57, ZFP932 and Gm15446 peaks. 33 ERVs were found to be triply co-bound. **c** Genome browser screenshot of a triply co-bound RLTR44E retroelement near the Ankrd10 gene. Notably, there is a partial loss of H3K9me3 under the ZFP57 peak in ZFP57 KO but not adjacent ZFP932/Gm15446 peaks, thus maintaining overall silencing of the repeat and unchanged levels of the proximal gene

In this figure, a browser snapshot shows the enrichment of ZFP57 around the cognate motif (highlighted in yellow) and not exactly at the motif on the RLTR repeat element

2. (Zhang *et al*, 2019)

Published data **Our data**

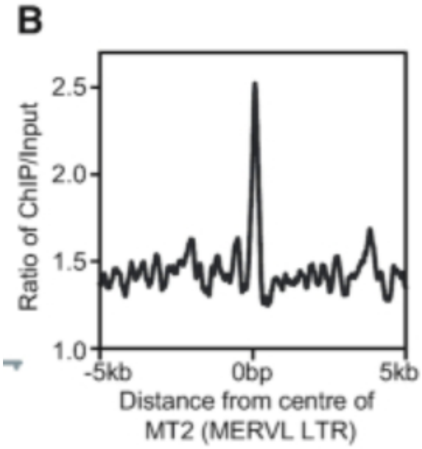

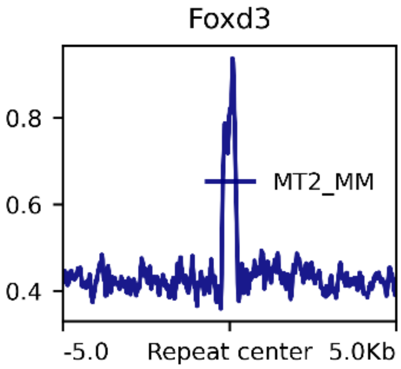

In this figure the enrichment of the TF ZSCAN4 is plotted with the data centred around the centre of the repeat element MT2 (MERVL LTR). This looks similar when we plot the Foxd3 enrichment over MT2 centred around the repeat. As is evident in this figure, FOXD3 enrichment forms a sharp peak which tapers as one moves away from the repeat centre.

Published data

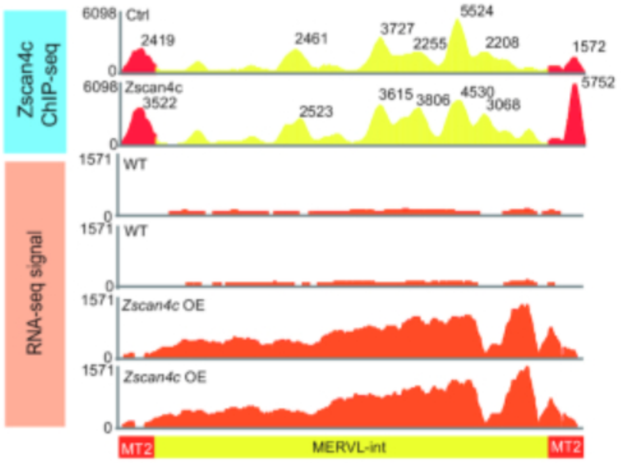

In this figure, the enrichment of ZSCAN4C is plotted along MERVL and it is clear that the enrichment appears to be broader along the repeat element and not a sharp peak as one would perhaps observe over a single copy gene.

3. (Wolf *et al*, 2020)
Figure 1 B

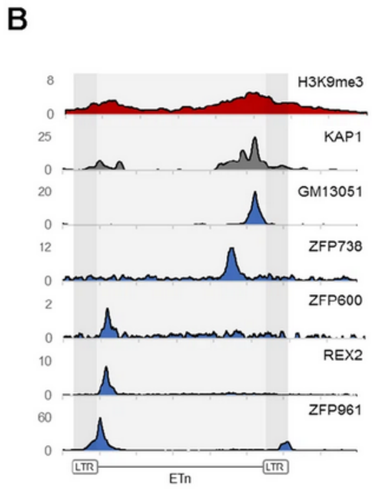

In this figure, the meta data profiles show the enrichment of various TFs over intact ERVs, while the peaks are narrower than seen in our data, some TFs seem to be enriched over broader regions especially over LTRs. ZFP600 and KAP1 particularly, are over the entire repeat element.
These examples and more suggest that the appearance of TF peaks over repeat elements are a function of mapping (Intact vs all annotated repeats) and protocols used for plotting the data (centred around repeat start and end, peak centre or repeat centre). The appearance of the peak not matching a classical TF peak over genes by no means diminishes the veracity of the data, as our further experiments prove the binding of FOXD3 to repeat elements.

Shi H, Strogantsev R, Takahashi N, Kazachenka A, Lorincz MC, Hemberger M & Ferguson-Smith AC (2019) ZFP57 regulation of transposable elements and gene expression within and beyond imprinted domains. *Epigenetics Chromatin* 12: 49
Wolf G, de Iaco A, Sun M-A, Bruno M, Tinkham M, Hoang D, Mitra A, Ralls S, Trono D & Macfarlan TS (2020) KRAB-zinc finger protein gene expansion in response to active retrotransposons in the murine lineage. *eLife* 9: e56337

Zhang W, Chen F, Chen R, Xie D, Yang J, Zhao X, Guo R, Zhang Y, Shen Y, Göke J, *et al* (2019) Zscan4c activates endogenous retrovirus MERVL and cleavage embryo genes. *Nucleic Acids Res* 47: 8485–8501

- **Despite the appearance of the peaks in the metadata, our further Foxd3 ChIP experiments using primers specific to different regions of MERVL indicate that the binding is limited to some areas of the repeat and not the whole repeat (Figure 1D, Figure EV1 E and F).**

- **Additionally, while we have predicted FOXD3 binding sites based on stringent cut-offs in our TF prediction algorithm, we do not exclude the possibility of other FOXD3 binding sites along the repeat sequence which were not predicted by our algorithm due to our cut-offs, which would still be enriched for FOXD3. Analysis of individual MERVL loci (Figure EV1D) indicates that FOXD3 is largely enriched over defined regions of MERVL which presumably correspond to the FOXD3 binding site.**

I can include a discussion on the appearance of the peak over the entire repeat in the text of the main manuscript as further clarification if advised.

In summary, I believe that our data satisfactorily proves, by multiple experimental approaches, the binding of FOXD3 to MERVL and MSR. We have addressed all the reviewers' comments in the course of two revisions.

Additionally, by the examples given in this email, we show that TF binding peaks appear as broader peaks in repeat elements. This appearance does not cast in doubt ,the study's findings or the rigour with which they were performed.

With regards to point 1, I will add more details in the Methods section to expand on our ChIP-Seq data analysis.

I request you to please advise whether I will be asked to submit the manuscript again and whether the manuscript will be re-sent to peer review. Please also let me know whether I will need to submit a point by point response to the reviewer’s comments (I have already done so in the last revision).

I look forward to hearing back from you.

Deepika Puri

Dear Deepika,

Thank you for the explanation. Referee 2 should have seen your revised files, and in fact, s/he comments on the removal of the heatmaps. However, I cannot be certain that s/he has seen all files, including Fig EV1, but I would assume so.

In any case, we have now received cross-comments from referee 3 as follows:

"I wouldn't go as far as saying that the ChIP-seq data generally contradict the conclusions. The metaphors show enrichment (but see more on this below) over MSR/MERVL, which is in line with all other data. But the referee was expecting a more localised enrichment, which I generally agree with. However, there could be resolution issues that prevent this, and even if it's true, then I think it's just a point for discussion (how localised is FOXD3 binding, and potential explanations) rather than something that dramatically questions their conclusions.

But I do agree that the normalisation of the metaplots is unclear. The authors do describe the tool they used to generate the plots (deepTools), but as far as I can tell this is just the ChIP signal, and that it was not normalised to an input. So the authors should either: a) add the input signal to the metaplots, or b) normalise the ChIP signal to the input. The authors say the plots display enrichment, but it's not clear if this means the data were normalised to input. The fact that the y axes on these plots have no label really doesn't help."

So in summary, it is fine if you discuss the Foxd3 binding to repeat elements in more detail in your manuscript, as you also suggest in your latest response. Importantly, it needs to be clarified whether normalization was done for the ChIP experiment, and if not, data need to be added along the lines referee 3 suggests here. All axes must also be labeled.

You can either send us the new files by email, or, if you prefer, I can make another decision that will allow you to upload new files. You can also bring forward the old manuscript files (now Version 3) during the process, so the old files do not need to be uploaded again. Please let me know what is easier for you.

Best regards,
Esther

Esther Schnapp, PhD
Editor | EMBO reports
eschnapp@wiley.com

Dear Esther,

Thank you very much for your email.

As I had mentioned earlier, we had normalised the ChIP signal to the Input signal at the time of peak calling with MACS before the plotting the data. However, after the comments from reviewers 2 and 3, to provide more clarity, we have made our data more stringent by adding RPGC normalization which normalizes the coverage of the reads in Input and ChIP. We have applied this normalization to all our ChIP -Seq analyses (Foxd3 Suv39h1 Suv39h2 and H3K9me3). In addition, we have also added a track for the Input for visual differentiation between the ChIP and Input signals for Foxd3, rather than just relative ChIP signal. As mentioned by reviewer 3 we have tried to increase the resolution of the peak by reducing the flanks to +/- 1KB.
I am also adding a negative control (L1MdA) to verify that the binding is specific to MSR and MERVL. So I will be making the following changes.

o Main manuscript- Changes marked in red: Methods, Discussion on FOXD3 peaks, Figure legends
o Figure 1: Figure 1C (Normalisation by RPGC and addition of input track)
o Figure 4: Figure 4C and F (Normalisation by RPGC)

These changes do not alter the results of the study but in fact with the input shown separately, help in better understanding the binding. I will also discuss the localisation of Foxd3 in the main manuscript as suggested by reviewer 3.
I will ensure that all the axes in the figures are labelled.

Because there will be 3 files that need to be replaced, maybe it is better to upload them into the online system as you suggested. I request you to please open the option of uploading for me.

Thank you for the clarification about more revisions.

Best regards,

Deepika Puri, PhD.
INSPIRE Faculty,
Lab7 NB, NCCS, Pune 411008

Revision Received **9th Sep 2021**

The authors have addressed all further editorial requests.

2nd Revision - Editorial Decision 14th Sep 2021

Dr. Deepika Puri
National Centre for Cell Science
Ganeshkhind road,
SP Pune University
Pune, Maharashtra 411007
India

Dear Dr. Puri,

I am very pleased to accept your manuscript for publication in the next available issue of EMBO reports. Thank you for your contribution to our journal.

At the end of this email I include important information about how to proceed. Please ensure that you take the time to read the information and complete and return the necessary forms to allow us to publish your manuscript as quickly as possible.

As part of the EMBO publication's Transparent Editorial Process, EMBO reports publishes online a Review Process File to accompany accepted manuscripts. As you are aware, this File will be published in conjunction with your paper and will include the referee reports, your point-by-point response and all pertinent correspondence relating to the manuscript.

If you do NOT want this File to be published, please inform the editorial office within 2 days, if you have not done so already, otherwise the File will be published by default [contact: emboreports@embo.org]. If you do opt out, the Review Process File link will point to the following statement: "No Review Process File is available with this article, as the authors have chosen not to make the review process public in this case."

Should you be planning a Press Release on your article, please get in contact with emboreports@wiley.com as early as possible, in order to coordinate publication and release dates.

Thank you again for your contribution to EMBO reports and congratulations on a successful publication. Please consider us again in the future for your most exciting work.

Yours sincerely,

Achim Breiling
Editor
EMBO Reports

****

THINGS TO DO NOW:

You will receive proofs by e-mail approximately 2-3 weeks after all relevant files have been sent to

our Production Office; you should return your corrections within 2 days of receiving the proofs.

Please inform us if there is likely to be any difficulty in reaching you at the above address at that time. Failure to meet our deadlines may result in a delay of publication, or publication without your corrections.

All further communications concerning your paper should quote reference number EMBOR-2021-53180V3 and be addressed to emboreports@wiley.com.

Should you be planning a Press Release on your article, please get in contact with emboreports@wiley.com as early as possible, in order to coordinate publication and release dates.

EMBO PRESS

YOU MUST COMPLETE ALL CELLS WITH A PINK BACKGROUND ➔

PLEASE NOTE THAT THIS CHECKLIST WILL BE PUBLISHED ALONGSIDE YOUR PAPER

Corresponding Author Name: Deepika Puri	
Journal Submitted to: EMBO Reports	
Manuscript Number: EMBOR-2021-53180V1	

Reporting Checklist For Life Sciences Articles (Rev. June 2017)

This checklist is used to ensure good reporting standards and to improve the reproducibility of published results. These guidelines are consistent with the Principles and Guidelines for Reporting Preclinical Research issued by the NIH in 2014. Please follow the journal's authorship guidelines in preparing your manuscript.

A- Figures
1. Data
The data shown in figures should satisfy the following conditions:

- ➔ the data were obtained and processed according to the field's best practice and are presented to reflect the results of the experiments in an accurate and unbiased manner.
- ➔ figure panels include only data points, measurements or observations that can be compared to each other in a scientifically meaningful way.
- ➔ graphs include clearly labeled error bars for independent experiments and sample sizes. Unless justified, error bars should not be shown for technical replicates.
- ➔ if n< 5, the individual data points from each experiment should be plotted and any statistical test employed should be justified
- ➔ Source Data should be included to report the data underlying graphs. Please follow the guidelines set out in the author ship guidelines on Data Presentation.

2. Captions
Each figure caption should contain the following information, for each panel where they are relevant:

- ➔ a specification of the experimental system investigated (eg cell line, species name).
- ➔ the assay(s) and method(s) used to carry out the reported observations and measurements
- ➔ an explicit mention of the biological and chemical entity(ies) that are being measured.
- ➔ an explicit mention of the biological and chemical entity(ies) that are altered/varied/perturbed in a controlled manner.

- ➔ the exact sample size (n) for each experimental group/condition, given as a number, not a range;
- ➔ a description of the sample collection allowing the reader to understand whether the samples represent technical or biological replicates (including how many animals, litters, cultures, etc.).
- ➔ a statement of how many times the experiment shown was independently replicated in the laboratory.
- ➔ definitions of statistical methods and measures:
  - common tests, such as t-test (please specify whether paired vs. unpaired), simple χ2 tests, Wilcoxon and Mann-Whitney tests, can be unambiguously identified by name only, but more complex techniques should be described in the methods section;
  - are tests one-sided or two-sided?
  - are there adjustments for multiple comparisons?
  - exact statistical test results, e.g., P values = x but not P values < x;
  - definition of 'center values' as median or average;
  - definition of error bars as s.d. or s.e.m.

Any descriptions too long for the figure legend should be included in the methods section and/or with the source data.

In the pink boxes below, please ensure that the answers to the following questions are reported in the manuscript itself. Every question should be answered. If the question is not relevant to your research, please write NA (non applicable). We encourage you to include a specific subsection in the methods section for statistics, reagents, animal models and human subjects.

USEFUL LINKS FOR COMPLETING THIS FORM

http://www.antibodypedia.com
http://1degreebio.org
http://www.equator-network.org/reporting-guidelines/improving-bioscience-research-repor
http://grants.nih.gov/grants/olaw/olaw.htm
http://www.mrc.ac.uk/Ourresearch/Ethicsresearchguidance/Useofanimals/index.htm
http://ClinicalTrials.gov
http://www.consort-statement.org
http://www.consort-statement.org/checklists/view/32-consort/66-title
http://www.equator-network.org/reporting-guidelines/reporting-recommendations-for-tumo
http://datadryad.org
http://figshare.com
http://www.ncbi.nlm.nih.gov/gap
http://www.ebi.ac.uk/ega
http://biomodels.net/
http://biomodels.net/miriam/
http://jjj.biochem.sun.ac.za
http://oba.od.nih.gov/biosecurity/biosecurity_documents.html
http://www.selectagents.gov/

B- Statistics and general methods

Please fill out these boxes ➔ (Do not worry if you cannot see all your text once you press return)

1.a. How was the sample size chosen to ensure adequate power to detect a pre-specified effect size?	All the data presented in the manuscript is obtained from three independent biological replicates unless specifically mentioned otherwise. For immunofluorescences, atleast 150 cells were counted per replicate to determine the percentage of cells depicted in the data.
1.b. For animal studies, include a statement about sample size estimate even if no statistical methods were used.	NA
2. Describe inclusion/exclusion criteria if samples or animals were excluded from the analysis. Were the criteria pre-established?	NA
3. Were any steps taken to minimize the effects of subjective bias when allocating animals/samples to treatment (e.g. randomization procedure)? If yes, please describe.	NA
For animal studies, include a statement about randomization even if no randomization was used.	NA
4.a. Were any steps taken to minimize the effects of subjective bias during group allocation or/and when assessing results (e.g. blinding of the investigator)? If yes please describe.	NA
4.b. For animal studies, include a statement about blinding even if no blinding was done	NA
5. For every figure, are statistical tests justified as appropriate?	Yes
Do the data meet the assumptions of the tests (e.g., normal distribution)? Describe any methods used to assess it.	NA
Is there an estimate of variation within each group of data?	NA

Is the variance similar between the groups that are being statistically compared?	NA

C- Reagents

6. To show that antibodies were profiled for use in the system under study (assay and species), provide a citation, catalog number and/or clone number, supplementary information or reference to an antibody validation profile. e.g., Antibodypedia (see link list at top right), 1DegreeBio (see link list at top right).	Foxd3: Merck Millipore, Catalog number AB5687. H3K9me3: crude serum, Antibody no 4861 (Jenuwein lab). H3K4me3: Diagenode, Catalog number C15410003-50. H3K27me3: Antibody no 6523 (Jenuwein lab), Suv39h1: Sigma Aldrich (Merck), Catalog number 05-615.ZSCAN4, Millipore, Catalog number AB4340. MERVL-gag, Hangzhou HuaAn Biotechnology, Catalog number R1501-2,. DPPA3, Abcam, Catalog number ab19878,GFP: Invitrogen, Catalog number A11122. Setdb1: ThermoFisher, Catalog number MA5-15722
7. Identify the source of cell lines and report if they were recently authenticated (e.g., by STR profiling) and tested for mycoplasma contamination.	The Foxd3 cKO cell lines were obtained from Patricia Labosky lab at the Vanderbildt University. The Suv39h dn ES cells were obtained from Thomas Jenuwein lab at the Max Planck Institute for immunobiology and Epigenetics

* for all hyperlinks, please see the table at the top right of the document

D- Animal Models

8. Report species, strain, gender, age of animals and genetic modification status where applicable. Please detail housing and husbandry conditions and the source of animals.	NA
9. For experiments involving live vertebrates, include a statement of compliance with ethical regulations and identify the committee(s) approving the experiments.	NA
10. We recommend consulting the ARRIVE guidelines (see link list at top right) (PLoS Biol. 8(6), e1000412, 2010) to ensure that other relevant aspects of animal studies are adequately reported. See author guidelines, under 'Reporting Guidelines'. See also: NIH (see link list at top right) and MRC (see link list at top right) recommendations. Please confirm compliance.	NA

E- Human Subjects

11. Identify the committee(s) approving the study protocol.	NA
12. Include a statement confirming that informed consent was obtained from all subjects and that the experiments conformed to the principles set out in the WMA Declaration of Helsinki and the Department of Health and Human Services Belmont Report.	NA
13. For publication of patient photos, include a statement confirming that consent to publish was obtained.	NA
14. Report any restrictions on the availability (and/or on the use) of human data or samples.	NA
15. Report the clinical trial registration number (at ClinicalTrials.gov or equivalent), where applicable.	NA
16. For phase II and III randomized controlled trials, please refer to the CONSORT flow diagram (see link list at top right) and submit the CONSORT checklist (see link list at top right) with your submission. See author guidelines, under 'Reporting Guidelines'. Please confirm you have submitted this list.	NA
17. For tumor marker prognostic studies, we recommend that you follow the REMARK reporting guidelines (see link list at top right). See author guidelines, under 'Reporting Guidelines'. Please confirm you have followed these guidelines.	NA

F- Data Accessibility

18: Provide a "Data Availability" section at the end of the Materials & Methods, listing the accession codes for data generated in this study and deposited in a public database (e.g. RNA-Seq data: Gene Expression Omnibus GSE39462, Proteomics data: PRIDE PXD000208 etc.) Please refer to our author guidelines for 'Data Deposition'.<br><br>Data deposition in a public repository is mandatory for:<br>a. Protein, DNA and RNA sequences<br>b. Macromolecular structures<br>c. Crystallographic data for small molecules<br>d. Functional genomics data<br>e. Proteomics and molecular interactions	The sequencing data generated and reported in this paper can be accessed in GEO using the accession number GSE173602. Link: https://www.ncbi.nlm.nih.gov/geo/query/acc.cgi?acc=GSE173602
19. Deposition is strongly recommended for any datasets that are central and integral to the study; please consider the journal's data policy. If no structured public repository exists for a given data type, we encourage the provision of datasets in the manuscript as a Supplementary Document (see author guidelines under 'Expanded View' or in unstructured repositories such as Dryad (see link list at top right) or Figshare (see link list at top right).	NA
20. Access to human clinical and genomic datasets should be provided with as few restrictions as possible while respecting ethical obligations to the patients and relevant medical and legal issues. If practically possible and compatible with the individual consent agreement used in the study, such data should be deposited in one of the major public access-controlled repositories such as dbGAP (see link list at top right) or EGA (see link list at top right).	NA
21. Computational models that are central and integral to a study should be shared without restrictions and provided in a machine-readable form. The relevant accession numbers or links should be provided. When possible, standardized format (SBML, CellML) should be used instead of scripts (e.g. MATLAB). Authors are strongly encouraged to follow the MIRIAM guidelines (see link list at top right) and deposit their model in a public database such as Biomodels (see link list at top right) or JWS Online (see link list at top right). If computer source code is provided with the paper, it should be deposited in a public repository or included in supplementary information.	NA

G- Dual use research of concern

22. Could your study fall under dual use research restrictions? Please check biosecurity documents (see link list at top right) and list of select agents and toxins (APHIS/CDC) (see link list at top right). According to our biosecurity guidelines, provide a statement only if it could.	NA
